# Visible-Light-Induced, Graphene Oxide-Promoted C3-Chalcogenylation of Indoles Strategy under Transition-Metal-Free Conditions

**DOI:** 10.3390/molecules27030772

**Published:** 2022-01-25

**Authors:** Qing Huang, Xiangjun Peng, Hong Li, Haiping He, Liangxian Liu

**Affiliations:** 1 Department of Chemistry and Chemical Engineering, Gannan Normal University, Ganzhou 341000, China; jxhq6200@163.com (Q.H.); Lihong9600428033@163.com (H.L.); 2 Key Laboratory of Prevention and Treatment of Cardiovascular and Cerebrovascular Diseases of Ministry of Education, School of Pharmaceutical Science of Gannan Medical University, Ganzhou 341000, China; hehaiping223@163.com

**Keywords:** graphene oxide, visible-light, indole, sulfenylindole, selenylindole

## Abstract

An efficient and general method for the synthesis of 3-sulfenylindoles and 3-selenylindoles employing visible-light irradiation with graphene oxide as a promoter at room temperature has been achieved. The reaction features are high yields, simple operation, metal-free and iodine-free conditions, an easy-to-handle oxidant, and gram-scalable synthesis. This simple protocol allows one to access a wide range of 3-arylthioindoles, 3-arylselenylindoles, and even 3-thiocyanatoindoles with good to excellent yields.

## 1. Introduction

**Figure 1:**
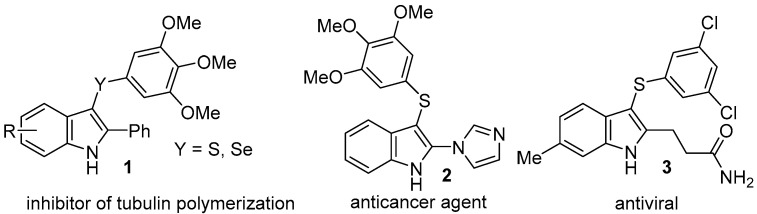
Selected examples of biologically active 3-selanyl- and 3-sulfanylindole compounds.

Organosulfur and organoselenium compounds, which possess broad biological and pharmaceutical activities, have been widely employed as important scaffolds for medicinal chemistry (Figure 1) [1,2,3,4,5,6]. Among them, 3-sulfenylindoles and 3-selenylindoles represent important classes of sulfur and selenium-containing compounds having more greater therapeutic values in the treatment of cancer [7,8,9,10,11,12], HIV [13,14,15], tubulin assembly inhibition [16,17], and bacterial diseases [18,19,20,21,22]. In this regard, numerous methods for the straightforward construction of C-S and C-Se bonds have been developed for the synthesis of 3-sulfenylindoles and 3-selenylindoles. Among these various approaches, the most commonly used methods involved the direct sulfenylation and selenation of the indole moieties with various electrophilic sulfur and selenium reagents [23,24,25,26,27,28,29,30,31,32,33,34,35].

However, these strategies suffer from limitations, such as the need for stoichiometric or super stoichiometric amounts of catalysts, strong acidic or oxidizing reagents, harsh reaction conditions, the complex synthetic process of activated sulfur or selenium reagents, and limited substrate scopes [36,37,38,39,40,41]. Most importantly, these reactions employ arylsulfur or arylselenium reagents such as benzenesulfonyl chlorides [42,43,44,45], *N*-(thiophenyl)succinimide [46,47], *S*-phenyl benzenesulfonothioate [48,49], disulfides [50,51,52], benzene-sulfonhydrazide [53,54,55,56], *N*-phenylselenophthalimide [57], *N*-phenylselenosuccinimide [58], and diselenides [59,60,61,62,63], generation of stoichiometric byproducts still cannot be avoided under the conditions used. Therefore, the development of green and sustainable synthetic methods is highly desirable under mild conditions so as to avoid the use of external oxidants, transition metal catalysts, or harsh reaction conditions.

In recent years, graphene oxide (GO) [64,65,66,67], which is a readily available and inexpensive material, has historically functioned primarily as a precursor to reduced graphene oxide (rGO) or chemically modified graphene (CMG) materials [68,69], and has generated tremendous excitement due to its potential applications in plastic electronics, solar cells, optical materials, and biosensors [70,71]. In addition, photo-induced organic transformations have emerged as an attractive and suitable approach in recent years [72,73,74,75,76,77,78,79,80,81]. Although GO has been reported as a photocatalyst for hydrogen production from water under UV irradiation [82], the potential application of GO in synthetic photochemistry is still rare [83].

**Scheme 1:**
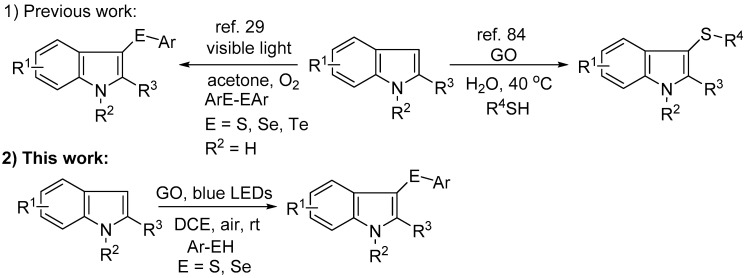
C3 chalcogenylation of indoles.

More recently, Wu et al developed a procedure of GO-mediated thiolation of indoles with thiols in water (Scheme 1) [84]. This methodology provided an atom economical and transition-metal and iodine free procedure for the direct synthesis of 3-sulfenylindoles. Subsequently, Kumar and Rathore reported a benign oxidant, photocatalyst and transition-metal-free visible light induced methodology for the construction of carbon-chalcogen (S, Se, Te) bond that enables the 3-chalcogenyl indole (Scheme 1) [29]. However, most of these methods suffer from some drawbacks such as low atom efficiency and limited substrate scope. Recently, we reported a new and efficient method for the C3-chalcogenylation of indolines employing visible-light irradiation and graphene oxide as a promoter at room temperature [85]. However, the reaction substrates are expensive and difficult to obtain for this synthesis method. In continuation of our work on indole chemistry [86,87,88,89,90,91,92] and GO-promoted C-H functionalisation of indoles [93], herein, we wish to report the combination of GO and blue LEDs, which works in synergy to efficiently promote the organo chalcogenylation (S and Se) of indoles in DCE under air atmosphere by using commercially available substrates. The highlight of this work is that GO not only acts as an oxidant, but as a photocatalyst as well.

## 2. Results and Discussion

The GO material used in this investigation was prepared by Hummers oxidation of graphite and subsequent exfoliation, as reported [94,95]. The obtained GO material was characterized by X-ray powder diffraction (XRD), transmission electron microscopy (TEM), visible Raman spectroscopy, and atomic force microscopy (AFM) [96] (see the Supplementary Materials).

To commence our investigation, the reaction of indole **4a** with 4-methylbenzenethiol **5a** was performed using 40 wt % GO as a promoter under irradiation with sunlight in open air (Table 1). The reaction proceeded and produced the desired coupling product **6a** with a 28% yield (entry 1). Different light sources, such as CWF bulb (22 W, λ_max_ = 365 ± 10 nm), green LED (1.0 W, λ_max_ = 530 ± 10 nm), and blue LED (3.0 W, λ_max_ = 425 ± 15 nm), were tested. Blue LED was more effective than other light sources, indicating the higher activity of GO in the presence of high-intensity blue light (entries 2–4). The reaction in the absence of a light source either failed to take place at room temperature (entry 5), or only a trace amount of the target product was formed (entry 17). The solvent also plays an important role in this transformation. DCE (1,2-dichloroethane) was more effective than the other tested solvents, such as THF, DMSO, toluene, DMF, and 1,4-dioxane (entries 6–11). Subsequent efforts were directed toward optimizing the GO loadings (entries 12–16). Whereas 50 wt % GO afforded 87% of the target product, decreasing the loading to 20 wt % GO was found to be sufficient to drive the cross-coupling reaction to quantitative conversion. No product was detected without GO. On the basis of our screening experiments, the best reaction condition is using 50 wt % GO in DCE and irradiation with blue LED in open air at 25 °C for 12 h, which afforded the desired product **6a** in high yield (87%, entry 14).

With the best experimental conditions for the synthesis of **6a** in hand, we first evaluated the efficiency of different substituted indoles **4** while keeping 4-methylbenzenethiol **5a** constant. Under the optimized conditions, the desired products **6aa**-**6ma** could be efficiently obtained in good to excellent yields (Table 2). Various substituted indoles **4**, i.e., electrondonating (EDG, R = Me, OMe, OBn) and electron-withdrawing (EWG, R = Cl, I, CN, CO_2_CH_3_) groups successfully afforded the corresponding 3-sulfenylindoles and had no significant effect on the reactivity and the regioselectivity of reactions. In general, the EDG were better than the EWG. Furthermore, the introduction of various groups at the N-1, C-2, -3, -4, -5, -6, or -7 position of the indoles all proceeded with **5a** under standard reaction conditions. Exceptions to this are 4-methylindole and methyl-4-indolecarboxylate, showing moderate sulfenylation yields (**6ha** and **6ia**), probably due to the steric hindrance effect (entries 8 and 9). Interestingly, introducing a methyl group at the C-3-position of the indole afforded the 2-sulfenylindole product **6ma** in 84% yield.

Next, a diverse array of arylthiols were employed as substrates to explore the scope of this reaction (Table 3). These substrates also showed high reactivity in this transformation. All reactions proceeded smoothly when the thiophenol was bearing, regardless of electron-donating groups (Me and OMe) or electron-withdrawing groups (Cl, Br, and NO_2_) on the phenyl ring; the 3-sulfenylindoles were obtained in good to excellent yields.

**Scheme 2:**
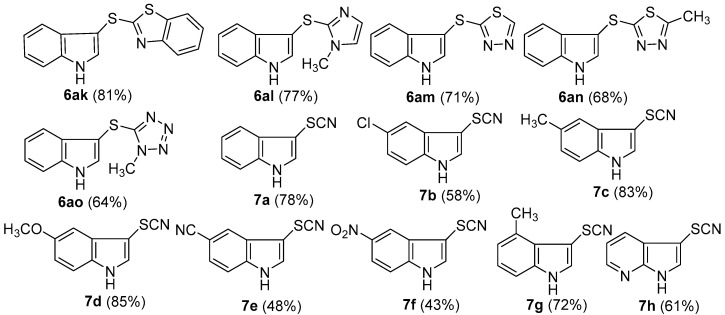
Heterocyclic thiols and potassium thiocyanate used as C3 sulfenylation of indoles.

The success in using aryl thiols encouraged us to examine the reaction of indole **4a** with various heterocyclic thiols including benzo[*d*]thiazole-2-thiol, 1-methyl-1*H*-imidazole-2-thiol, 1,3,4-thiadiazole-2-thiol, 5-methyl-1,3,4-thiadiazole-2-thiol, 1-methyl-1*H*-tetrazole-5-thiol, and the results are summarized in Scheme 2. In general, the desired products were formed in moderate to excellent yields under the standard reaction conditions.

Organothiocyanates are valuable synthetic intermediates which can be easily transformed into an array of organosulfur molecules [97,98,99]. Under the optimized conditions, we sustained our studies by treating indoles or 1*H*-pyrrolo[2,3-*b*]pyridine with KSCN under the standard reaction conditions, and the corresponding thiocyanated product **7a**–**f** were obtained with 43–85% yields (Scheme 2). The results have shown that electronegativities of substituents play a major role in governing the reactivity of the substrates. Electron-donating substitutents show better results than electron-withdrawing substitutents in this transformation.

**Scheme 3:**
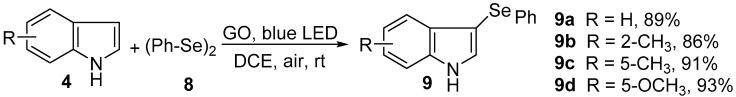
Synthesis of 3-selenylindoles. Reaction conditions: **4** (0.3 mmol), **8** (0.36 mmol), GO (50 wt %), and DCE (1 mL), for 8 h at rt under open air. Isolated yield.

The developed protocol can also be applied for the preparation of 3-selenyl-indoles using various indole derivatives **4** and diphenyl diselenide **8**. In general, the desired products **9** were formed in good to excellent yields in 8 h (Scheme 3), which was more efficient than the generation of 3-sulfenylindoles with regard to the yields and reaction times.

**Scheme 4:**
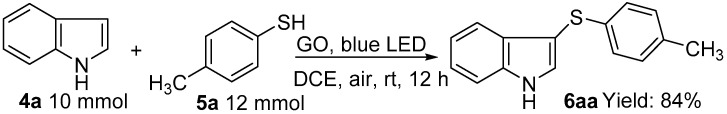
Scale-up reaction between **4a** and **5a**.

In order to demonstrate the effectiveness of this new strategy, a gram scale reaction was performed under the standard conditions. 10 mmol indole **4a** and 12 mmol 4-methylbenzenethiol **5a** were subjected to the reaction in the presence of GO (468 mg, 40 wt %) in 50 mL DCE at room temperature. After 12 h, the desired product **6a** was obtained in 84% yield, which demonstrated the practical application of this protocol to prepare 3-sulfenylindoles on a gram-scale (Scheme 4). To our delight, when the amount of GO was reduced to 40 wt %, the yield was not affected to any observable extent.

**Scheme 5:**
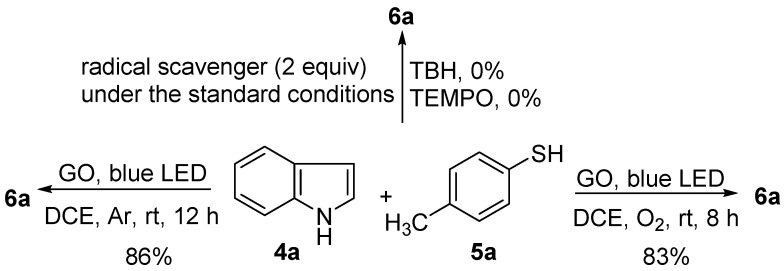
Control experiments.

To gain some insight into the mechanism of this reaction, some control experiments were conducted as shown in Scheme 5. Because the visible-light-induced, GO-promoted cross-coupling reaction was performed under open air, the role of O_2_ in this reaction was explored. Initially, When the optimal reaction was performed under an oxygen atmosphere instead of open air, there was no effect on the yield, but a faster conversion of the starting material to the reaction product was observed, indicating that O_2_ could be involved in the reaction pathway. Similarly, when the reaction was carried out under an argon atmosphere, no major effect was observed, indicating that the reaction follows a different route in an argon environment.

Then, radical trapping experiments were conducted by adding butylated hydroxytoluene (BHT) or 2,2,6,6-tetramethyl-1-piperidinyloxy (TEMPO) into the standard conditions of **4a** and **5a**. Experimental results show that these reaction were completely inhibited, indicating the involvement of radical species in the transformation.

**Scheme 6:**
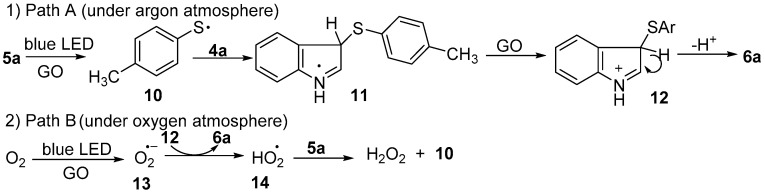
The proposed mechanism for the reaction.

On the basis of our control experiments and several other reports from the literature [29,85,100,101,102], we proposed two plausible mechanisms for this reaction in argon and in oxygen environments as shown in Scheme 6. Graphene oxide might act as a radical initiator [29]. Under an argon atmosphere (path A), promoted by the functional groups on the surface of GO, 5-methylbenzenethiol transformed into phenylthiophenol radical **10**. Next, the thioyl radical **10** interacted with **4a** to produce the radical intermediate **11**. After that, **11** was oxidized to the intermediate **12**. Finally, deprotonation of intermediate **12** led to the formation of product **6a**. GO probably plays a crucial role during the process of oxidation and deprotonation.

In 2012, Loh et al suggested that the edge sites with unpaired electrons in GO constitute the active catalytic sites and afford enhanced kinetics for the trapping and activation of molecular oxygen by a sequence of electron transport and reduction to superoxide radical [103,104]. Thus, in the case of an oxygen atmosphere (path B), the anion radical of O^2^ (O^2•−^), which is produced through a SET from unpaired electrons in GO, would abstract a proton from **12**, which would generate the desired product **6a** and perhydroxyl radical (HO_2_^•^). The transfer of H^•^ from **5a** to HO_2_^•^ would generate **10** and H_2_O_2_.

## 3. Materials and Methods

### 3.1. General Information

Unless otherwise specified, commercial reagents and solvents were used without further purification. Commercially available chemicals were purchased from Leyan (Shanghai, China) and used without any further purification. ^1^H and ^13^C NMR spectra were recorded on a Bruker spectrometer at 400 and 100 MHz, respectively. The chemical shifts were given in parts per million relative to CDCl_3_ (7.26 ppm for ^1^H) and CDCl_3_ (77.0 ppm for ^13^C. Peak multiplicities were reported as follows: s, singlet; d, doublet; t, triplet; m, multiplet; br. s, broad singlet and *J*, coupling constant (Hz). Mass spectra were recorded with Bruker Dalton Esquire 3000 plus LC-MS apparatus. Elemental analyses are expressed as percentage values. HRFABMS spectra were recorded on a FTMS apparatus. Silica gel (300–400 mesh) was used for flash column chromatography, eluting (unless otherwise stated) with an ethyl acetate/petroleum ether (PE) (60–90 °C) mixture.

### 3.2. General Procedure of the Products ***6***

In a 10 mL Schlenk tube, indole (0.3 mmol), GO (17.6 mg), and thiol (0.36 mmol) were stirred in DCE (1 mL) for 12 h at room temperature under an air atmosphere irradiated by blue LEDs. The reaction mixture was concentrated under reduced pressure. The residue was purified by flash chromatography on silica gel (eluent: EtOAc/PE = 1:10) to yield the corresponding product **6**.

3-(*p*-Tolylthio)-1*H*-indole (**6aa**). Yellow amorphous solid. ^1^H NMR (400 MHz, CDCl_3_): *δ* 8.37 (s, 1H), 7.63 (d, *J* = 7.9 Hz, 1H), 7.46 (d, *J* = 2.6 Hz, 1H), 7.43 (d, *J* = 8.1 Hz, 1H), 7.26 (dt, *J* = 1.0, 8.1 Hz, 1H, Ar-H), 7.18 (t, *J* = 7.1 Hz, 1H), 7.05 (d, *J* = 8.3 Hz, 2H), 6.99 (d, *J* = 8.3 Hz, 2H), 2.26 (s, 3H). ^13^C NMR (101 MHz, CDCl_3_): *δ* 136.5, 135.5, 134.7, 130.4, 129.5, 129.1, 126.3, 123.0, 120.8, 119.7, 111.5, 103.6, 20.8. MS (ESI): 240 (M + H^+^, 100). These assignments matched with those previously published [27].

5-Iodo-3-(*p*-tolylthio)-1*H*-indole (**6ba**). Brown amorphous solid. ^1^H NMR (400 MHz, CDCl_3_): *δ* 8.51 (s, 1H, NH), 7.99 (d, *J* = 1.5 Hz, 1H, Ar-H), 7.53 (dd, *J* = 8.5, 1.5 Hz, 1H, Ar-H), 7.43 (d, *J* = 1.5 Hz, 1H, Ar-H), 7.21 (d, *J* = 8.5 Hz, 1H, Ar-H), 7.06–7.01 (m, 4H, Ar-H), 2.29 (s, 3H, CH_3_). ^13^C NMR (101 MHz, CDCl_3_): *δ* 135.6, 135.1, 134.9, 131.7, 131.4, 131.3, 129.6, 128.4, 126.3, 113.6, 102.8, 84.6, 20.9. MS (ESI): 366 (M + H^+^, 100). These assignments matched with those previously published [105].

5-Methyl-3-(*p*-tolylthio)-1*H*-indole (**6ca**). Yellow amorphous solid. ^1^H NMR (400 MHz, CDCl_3_): *δ* 8.19 (s, 1H, NH), 7.55 (d, *J* = 0.7 Hz, 1H, Ar-H), 7.40 (d, *J* = 2.6 Hz, 1H, Ar-H), 7.34 (d, *J* = 8.2 Hz, 1H, Ar-H), 7.21–7.13 (m, 3H, Ar-H), 7.08 (d, *J* = 8.2 Hz, 2H, Ar-H), 2.52 (s, 3H, CH_3_), 2.36 (s, 3H, CH_3_). ^13^C NMR (101 MHz, CDCl_3_): *δ* 135.9, 134.9, 134.7, 130.94, 130.4, 129.7, 129.5, 126.2, 124.7, 119.2, 111.5, 102.4, 21.6, 21.0. MS (ESI): 254 (M + H^+^, 100). These assignments matched with those previously published [105].

3-(*p*-Tolylthio)-1*H*-indole-5-carbonitrile (**6da**). Yellow amorphous solid. ^1^H NMR (400 MHz, DMSO-*d_6_*): *δ* 12.20 (s, 1H, NH), 7.99 (d, *J* = 1.6 Hz, 1H, Ar-H), 7.81 (s, 1H, Ar-H), 7.66 (d, *J* = 8.4 Hz, 1H, Ar-H), 7.53 (d, *J* = 8.4 Hz, 1H, Ar-H), 7.04 (d, *J* = 8.0 Hz, 2H, Ar-H), 6.99 (d, *J* = 8.0 Hz, 2H, Ar-H), 2.20 (s, 3H, CH_3_). ^13^C NMR (101 MHz, DMSO-*d_6_*): *δ* 139.1, 135.3, 135.2, 134.9, 130.1, 129.0, 126.9, 125.4, 124.1, 120.7, 114.3, 102.8, 102.5, 20.9. MS (ESI): 265 (M + H^+^, 100). These assignments matched with those previously published [106].

6-Methoxy-3-(*p*-tolylthio)-1*H*-indole (**6ea**). Reddish brown amorphous solid. ^1^H NMR (400 MHz, CDCl_3_): *δ* 8.33 (s, 1H, NH), 7.51 (d, *J* = 8.6 Hz, 1H, Ar-H), 7.34 (d, *J* = 2.2 Hz, 1H, Ar-H), 7.08 (d, *J* = 8.2 Hz, 2H, Ar-H), 7.02 (d, *J* = 8.2 Hz, 2H, Ar-H), 6.90 (d, *J* = 2.2 Hz, 1H, Ar-H), 6.86 (dd, *J* = 8.6, 2.2 Hz, 1H, Ar-H), 3.87 (s, 3H, OCH_3_), 2.29 (s, 3H, CH_3_). ^13^C NMR (101 MHz, CDCl_3_): *δ* 157.2, 137.3, 135.6, 134.7, 129.5, 129.3, 126.3, 123.3, 120.3, 110.8, 103.4, 95.2, 55.7, 20.9. MS (ESI): 270 (M + H^+^, 100). These assignments matched with those previously published [106].

7-(Benzyloxy)-3-(*p*-tolylthio)-1*H*-indole (**6ga**). Reddish brown amorphous solid. ^1^H NMR (400 MHz, CDCl_3_): *δ* 8.71 (s, 1H, NH), 7.52 (d, *J* = 7.1 Hz, 2H, Ar-H), 7.49–7.40 (m, 4H, Ar-H), 7.27 (d, *J* = 8.4 Hz, 1H, Ar-H), 7.09 (t, *J* = 7.8 Hz, 1H, Ar-H), 7.07 (d, *J* = 8.0 Hz, 2H, Ar-H), 7.01 (d, *J* = 8.0 Hz, 2H, Ar-H), 6.81 (d, *J* = 7.8 Hz, 1H, Ar-H), 5.24 (s, 2H, OCH_2_), 2.28 (s, 3H, CH_3_). ^13^C NMR (101 MHz, CDCl_3_): *δ* 145.6, 136.9, 135.7, 134.6, 130.8, 130.1, 129.5, 128.7, 128.3, 128.0, 127.2, 126.3, 121.2, 112.6, 104.0, 103.7, 70.4, 20.9. MS (ESI): 346 (M + H^+^, 100). Anal calcd for C_22_H_19_NOS: C, 76.49; H, 5.54; N, 4.05; S, 9.28. Found C, 76.35; H, 5.47; N, 4.33; S, 8.95.

4-Methyl-3-(*p*-tolylthio)-1*H*-indole (**6ha**). Reddish brown amorphous solid. ^1^H NMR (400 MHz, CDCl_3_) *δ* 8.41 (s, 1H, NH), 7.43 (d, *J* = 2.6 Hz, 1H, Ar-H), 7.29 (d, *J* = 8.1 Hz, 1H, Ar-H), 7.17 (t, *J* = 8.1 Hz, 1H, Ar-H), 7.06–6.98 (m, 4H, Ar-H), 6.92 (d, *J* = 7.1 Hz, 1H), 2.70 (s, 3H, CH_3_), 2.29 (s, 3H, CH_3_). ^13^C NMR (101 MHz, CDCl_3_): *δ* 137.9, 137.0, 134.3, 132.2, 131.7, 129.6, 127.0, 125.5, 123.1, 122.4, 109.4, 102.9, 20.9, 18.7. MS (ESI): 254 (M + H^+^, 100). These assignments matched with those previously published [106].

Methyl 3-(*p*-tolylthio)-1*H*-indole-4-carboxylate (**6ia**). Brown amorphous solid. ^1^H NMR (400 MHz, CDCl_3_): *δ* 9.19 (s, 1H, NH), 7.51 (d, *J* = 2.6 Hz, 1H, Ar-H), 7.49 (d, *J* = 1.0 Hz, 1H, Ar-H), 7.38 (d, *J* = 2.6 Hz, 1H, Ar-H), 7.24 (t, *J* = 7.8 Hz, 1H, Ar-H), 6.98 (s, 4H, Ar-H), 3.68 (s, 3H), 2.30 (d, *J* = 37.7 Hz, 3H). ^13^C NMR (101 MHz, CDCl_3_): *δ* 169.6, 137.6, 136.4, 134.6, 133.4, 129.4, 126.2, 125.4, 125.3, 122.1, 122.0, 115.1, 103.1, 51.9, 20.8. MS (ESI): 298 (M + H^+^, 100). Anal calcd for C_17_H_15_NO_2_S: C, 68.66; H, 5.08; N, 4.71; S, 10.78. Found C, 68.80; H, 5.26; N, 4.64; S, 10.57.

2-Methyl-3-(*p*-tolylthio)-1*H*-indole (**6ja**). Reddish brown amorphous solid. ^1^H NMR (400 MHz, CDCl_3_): *δ* 8.26 (s, 1H, NH), 7.58 (d, *J* = 7.8 Hz, 1H, Ar-H), 7.36 (dt, *J* = 1.0, 7.8 Hz, 1H, Ar-H), 7.22 (dt, *J* = 1,0, 7.8 Hz, 1H, Ar-H), 7.15 (dt, *J* = 1.0, 7.8 Hz, 1H, Ar-H), 6.99 (s, 4H, Ar-H), 2.54 (s, 3H, CH_3_), 2.27 (s, 3H, CH_3_). ^13^C NMR (101 MHz, CDCl_3_): *δ* 141.0, 135.7, 135.5, 134.4, 130.4, 129.5, 125.8, 122.1, 120.7, 119.0, 110.7, 99.9, 20.9, 12.2. MS (ESI): 254 (M + H^+^, 100). These assignments matched with those previously published [106].

1-Methyl-3-(*p*-tolylthio)-1*H*-indole (**6ka**). Reddish brown amorphous solid. ^1^H NMR (400 MHz, CDCl_3_): *δ* 7.75 (d, *J* = 8.2 Hz, 1H, Ar-H), 7.45 (d, *J* = 8.2 Hz, 1H, Ar-H), 7.39 (dt, *J* = 1.0, 7.0 Hz, 1H, Ar-H), 7.37 (s, 1H, Ar-H), 7.28 (dt, *J* = 1.0, 7.0 Hz, 1H, Ar-H), 7.15 (d, *J* = 8.2 Hz, 2H, Ar-H), 7.07 (d, *J* = 8.2 Hz, 1H, Ar-H), 3.86 (s, 3H, NCH_3_), 2.35 (s, 3H, CH_3_). ^13^C NMR (101 MHz, CDCl_3_): *δ* 137.6, 136.1, 134.9, 134.6, 123.0, 129.6, 126.3, 126.2, 122.6, 120.5, 119.8, 109.8, 101.3, 33.1, 21.0. MS (ESI): 254 (M + H^+^, 100). These assignments matched with those previously published [105].

2,5-Dimethyl-3-(*p*-tolylthio)-1*H*-indole (**6la**). Reddish brown amorphous solid. ^1^H NMR (400 MHz, CDCl_3_): *δ* 8.03 (s, 1H, NH), 7.46 (s, 1H, Ar-H), 7.25 (d, *J* = 8.2 Hz, 1H, Ar-H), 7.10 (dd, *J* = 8.2, 1.2 Hz, 1H, Ar-H), 7.46 (s, 4H, Ar-H), 2.51 (s, 3H, CH_3_), 2.49 (s, 3H, CH_3_), 2.34 (s, 3H, CH_3_). ^13^C NMR (101 MHz, CDCl_3_): *δ* 141.3, 136.0, 134.3, 133.8, 130.7, 130.1, 129.6, 125.7, 123.7, 118.7, 110.5, 99.0, 21.5, 20.9, 12.1. MS (ESI): 268 (M + H^+^, 100). These assignments matched with those previously published [106].

3-Methyl-2-(*p*-tolylthio)-1*H*-indole (**6ma**). Reddish brown amorphous solid. ^1^H NMR (400 MHz, CDCl_3_): *δ* 7.96 (s, 1H, NH), 7.71 (d, *J* = 7.9 Hz, 1H, Ar-H), 7.33 (d, *J* = 3.6 Hz, 2H, Ar-H), 7.26 (m, 1H, Ar-H), 7.12 (d, *J* = 8.3 Hz, 2H, Ar-H), 7.09 (d, *J* = 8.3 Hz, 2H, Ar-H), 2.51 (s, 3H, CH_3_), 2.38 (s, 3H, CH_3_). ^13^C NMR (101 MHz, CDCl_3_): *δ* 136.9, 135.9, 133.5, 130.0, 128.6, 127.1, 123.4, 122.3, 119.7, 119.5, 119.4, 111.0, 21.0, 9.6. MS (ESI): 254 (M + H^+^, 100). These assignments matched with those previously published [106].

3-((4-Chlorophenyl)thio)-1*H*-indole (**6ab**). Light yellow amorphous solid. ^1^H NMR (400 MHz, CDCl_3_): *δ* 8.47 (s, 1H, NH), 7.58 (d, *J* = 8.0 Hz, 1H, Ar-H), 7.49 (d, *J* = 2.6 Hz, 1H, Ar-H), 7.45 (d, *J* = 8.2 Hz, 1H, Ar-H), 7.29 (dt, *J* = 1.0, 8.0 Hz, 1H, Ar-H), 7.18 (dt, *J* = 1.0, 8.0 Hz, 1H, Ar-H), 7.12 (d, *J* = 8.7 Hz, 2H, Ar-H), 7.02 (d, *J* = 8.7 Hz, 2H, Ar-H). ^13^C NMR (101 MHz, CDCl_3_): *δ* 137.8, 136.5, 130.6, 130.5, 128.7, 128.6, 127.1, 123.1, 121.0, 119.4, 111.6, 102.4. MS (ESI): 260 (M + H^+^, 30), 262 (M + H^+^, 100). These assignments matched with those previously published [105].

3-((4-Bromophenyl)thio)-1*H*-indole (**6ac**). Brown amorphous solid. ^1^H NMR (400 MHz, DMSO-*d_6_*): *δ* 11.76 (s, 1H, NH), 7.80 (d, *J* = 2.7 Hz, 1H, Ar-H), 7.51 (d, *J* = 8.1 Hz, 1H, Ar-H), 7.41–7.35 (m, 3H, Ar-H), 7.20 (dt, *J* = 1.1, 8.1 Hz, 1H, Ar-H), 7.08 (dt, *J* = 1.1, 8.1 Hz, 1H, Ar-H), 6.96 (dt, *J* = 2.7, 8.6 Hz, 2H, Ar-H). ^13^C NMR (101 MHz, DMSO-*d_6_*): *δ* 139.5, 137.3, 133.1, 132.1, 128.9, 127.7, 122.7, 120.7, 118.6, 118.0, 112.9, 99.1. MS (ESI): 304 (M + H^+^, 100), 306 (M + H^+^, 100). These assignments matched with those previously published [105].

3-((4-Methoxyphenyl)thio)-1*H*-indole (**6ad**). Brown amorphous solid. ^1^H NMR (400 MHz, CDCl_3_): *δ* 8.38 (s, 1H, NH), 7.63 (d, *J* = 8.0 Hz, 1H, Ar-H), 7.46 (d, *J* = 2.6 Hz, 1H, Ar-H), 7.41 (d, *J* = 8.0 Hz, 1H, Ar-H), 7.25 (dt, *J* = 1.0, 8.0 Hz, 1H, Ar-H), 7.17 (dt, *J* = 1.0, 8.0 Hz, 1H, Ar-H), 7.13 (d, *J* = 8.9 Hz, 2H, Ar-H), 6.74 (d, *J* = 8.9 Hz, 2H, Ar-H), 3.73 (s, 3H, OCH_3_). ^13^C NMR (101 MHz, CDCl_3_): *δ* 157.8, 136.5, 123.0, 129.5, 129.0, 128.6, 122.9, 120.8, 119.7, 114.5, 111.5, 104.7, 55.3. MS (ESI): 256 (M + H^+^, 100). These assignments matched with those previously published [27].

3-((4-Ethylphenyl)thio)-1*H*-indole (**6ae**). Reddish brown amorphous solid. ^1^H NMR (400 MHz, CDCl_3_): *δ* 8.34 (s, 1H, NH), 7.71 (d, *J* = 8.0 Hz, 1H, Ar-H), 7.45 (d, *J* = 2.3 Hz, 1H, Ar-H), 7.44 (d, *J* = 8.0 Hz, 1H, Ar-H), 7.32 (dt, *J* = 1.0, 8.0 Hz, 1H, Ar-H), 7.23 (t, *J* = 7.5 Hz, 1H, Ar-H), 7.13 (d, *J* = 8.2 Hz, 2H, Ar-H), 7.06 (d, *J* = 8.2 Hz, 2H, Ar-H), 2.61 (q, *J* = 7.6 Hz, 2H, CH_2_), 1.23 (t, *J* = 7.6 Hz, 3H, CH_3_). ^13^C NMR (101 MHz, CDCl_3_): *δ* 141.3, 136.5, 135.9, 130.7, 129.2, 128.5, 126.3, 123.1, 120.9, 119.7, 111.8, 103.2, 28.4, 15.7. MS (ESI): 254 (M + H^+^, 100). Anal calcd for C_16_H_15_NS: C, 75.85; H, 5.97; N, 5.53; S, 12.65. Found C, 75.59; H, 5.63; N, 5.71; S, 12.32.

3-((2,4-Dimethylphenyl)thio)-1*H*-indole (**6af**). Tawny amorphous solid. ^1^H NMR (400 MHz, CDCl_3_): *δ* 8.28 (s, 1H, NH), 7.71 (d, *J* = 8.0 Hz, 1H, Ar-H), 7.45 (d, *J* = 8.0 Hz, 1H, Ar-H), 7.41 (d, *J* = 2.6 Hz, 1H, Ar-H), 7.36 (t, *J* = 7.7 Hz, 1H, Ar-H), 7.27 (t, *J* = 7.7 Hz, 1H, Ar-H), 7.09 (s, 1H, Ar-H), 6.83 (d, *J* = 8.0 Hz, 1H, Ar-H), 6.78 (d, *J* = 8.0 Hz, 1H, Ar-H), 2.59 (s, 3H, CH_3_), 2.34 (s, 3H, CH_3_). ^13^C NMR (101 MHz, CDCl_3_): *δ* 136.6, 134.7, 134.6, 134.4, 130.9, 130.5, 129.3, 127.1, 126.1, 123.0, 120.8, 119.7, 111.6, 103.0, 20.7, 19.9. MS (ESI): 254 (M + H^+^, 100). Anal calcd for C_16_H_15_NS: C, 75.85; H, 5.97; N, 5.53; S, 12.65. Found C, 76.04; H, 5.83; N, 5.66; S, 12.35.

3-((4-Nitrophenyl)thio)-1*H*-indole (**6ag**). Reddish brown amorphous solid. ^1^H NMR (400 MHz, CDCl_3_): *δ* 8.86 (s, 1H, NH), 8.02 (d, *J* = 9.0 Hz, 2H, Ar-H), 7.55 (t, *J* = 7.7 Hz, 1H, Ar-H), 7.54 (d, *J* = 2.6 Hz, 1H, Ar-H), 7.53 (d, *J* = 8.1 Hz, 1H, Ar-H), 7.34 (t, *J* = 8.1 Hz, 1H, Ar-H), 7.22 (t, *J* = 7.7 Hz, 1H, Ar-H), 7.15 (d, *J* = 9.0 Hz, 2H, Ar-H). ^13^C NMR (101 MHz, CDCl_3_): *δ* 150.0, 144.9, 136.7, 131.4, 128.5, 125.2, 123.9, 123.5, 121.4, 119.2, 112.1, 100.1. MS (ESI): 271 (M + H^+^, 100). These assignments matched with those previously published [107].

3-(Naphthalen-2-ylthio)-1*H*-indole (**6ah**). Reddish brown amorphous solid. ^1^H NMR (400 MHz, DMSO-*d_6_*): *δ* 11.74 (s, 1H, NH), 7.83 (d, *J* = 2.5 Hz, 1H, Ar-H), 7.77 (d, *J* = 8.6 Hz, 1H, Ar-H), 7.73 (d, *J* = 8.7 Hz, 1H, Ar-H), 7.61 (d, *J* = 7.5 Hz, 1H, Ar-H), 7.50 (d, *J* = 2.6 Hz, 1H, Ar-H), 7.48 (s, 1H, Ar-H), 7.40–7.33 (m, 3H, Ar-H), 7.20 (dd, *J* = 2.6 Hz, 1H, Ar-H), 7.17 (t, *J* = 2.6 Hz, 1H, Ar-H), 7.03 (t, *J* = 7.5 Hz, 1H, Ar-H). ^13^C NMR (101 MHz, DMSO-*d_6_*): *δ* 137.3, 137.2, 133.7, 133.0, 131.3, 129.1, 128.8, 128.1, 127.1, 127.0, 125.7, 124.9, 123.3, 122.6, 120.6, 118.8, 112.9, 99.8. MS (ESI): 276 (M + H^+^, 100). These assignments matched with those previously published [106].

5-Methoxy-3-((4-methoxyphenyl)thio)-1*H*-indole (**6ai**). Red amorphous solid. ^1^H NMR (400 MHz, CDCl_3_): *δ* 8.42 (s, 1H, NH), 7.40 (d, *J* = 2.6 Hz, 1H, Ar-H), 7.28 (d, *J* = 8.8 Hz, 1H, Ar-H), 7.17 (dt, *J* = 2.6, 8.8 Hz, 2H, Ar-H), 7.13 (d, *J* = 2.4 Hz, 1H, Ar-H), 6.95 (dd, *J* = 8.8, 2.4 Hz, 1H, Ar-H), 6.80 (dt, *J* = 2.6, 8.8 Hz, 2H, Ar-H), 3.84 (s, 3H, OCH_3_), 3.76 (s, 3H, OCH_3_). ^13^C NMR (101 MHz, CDCl_3_): *δ* 157.8, 155.0, 131.5, 131.0, 129.9, 129.8, 128.3, 114.6, 113.4, 112.5, 103.7, 104.0, 55.9, 55.4. MS (ESI): 286 (M + H^+^, 100). These assignments matched with those previously published [108].

3-(*p*-Tolylthio)-1*H*-pyrrolo[3,2-*b*]pyridine (**6aj**). Light yellow amorphous solid. ^1^H NMR (400 MHz, DMSO-*d_6_*): *δ* 11.86 (s, 1H, NH), 8.36 (d, *J* = 4.0 Hz, 1H, Ar-H), 8.00 (d, *J* = 2.6 Hz, 1H, Ar-H), 7.86 (d, *J* = 8.1 Hz, 1H, Ar-H), 7.19 (dd, *J* = 8.1, 4.5 Hz, 1H, Ar-H), 7.00 (d, *J* = 7.2 Hz, 2H, Ar-H), 6.96 (d, *J* = 7.2 Hz, 2H, Ar-H), 2.19 (s, 3H, CH_3_). ^13^C NMR (101 MHz, DMSO-*d_6_*): *δ* 146.0, 143.8, 136.2, 135.9, 134.5, 129.8, 129.7, 126.5, 120.0, 117.7, 101.6, 20.9. MS (ESI): 241 (M + H^+^, 100). Anal calcd for C_14_H_12_N_2_S: C, 69.97; H, 5.03; N, 11.66; S, 13.34. Found C, 70.21; H, 5.37; N, 11.31; S, 13.15.

2-((1*H*-indol-3-yl)thio)benzo[*d*]thiazole (**6ak**). Brown amorphous solid. ^1^H NMR (400 MHz, DMSO-*d_6_*): *δ* 12.03 (s, 1H, NH), 8.04 (d, *J* = 2.8 Hz, 1H, Ar-H), 7.82 (dd, *J* = 2.8, 1.8 Hz, 1H, Ar-H), 7.80 (dd, *J* = 2.1, 1.0 Hz, 1H, Ar-H), 7.57 (d, *J* = 7.8 Hz, 1H, Ar-H), 7.56 (d, *J* = 7.8 Hz, 1H, Ar-H), 7.41 (dt, *J* = 1.2, 8.4 Hz, 1H, Ar-H), 7.30–7.23 (m, 2H, Ar-H), 7.15 (dt, *J* = 1.0, 7.1 Hz, 1H, Ar-H). ^13^C NMR (101 MHz, DMSO-*d_6_*): *δ* 173.8, 154.6, 137.2, 135.4, 134.4, 128.4, 126.6, 124.4, 123.1, 122.1, 121.6, 121.3, 118.5, 113.1, 97.7. MS (ESI): 283 (M + H^+^, 100). These assignments matched with those previously published [101].

3-((1-Methyl-1*H*-imidazol-2-yl)thio)-1*H*-indole (**6al**). Yellow amorphous solid. ^1^H NMR (400 MHz, DMSO-*d_6_*): *δ* 11.51 (s, 1H, NH), 7.69 (s, 1H, Ar-H), 7.60 (d, *J* = 7.6 Hz, 1H, Ar-H), 7.39 (d, *J* = 7.6 Hz, 1H, Ar-H), 7.16 (s, 1H, Ar-H), 7.10 (t, *J* = 7.1 Hz, 1H, Ar-H), 7.04 (t, *J* = 7.1 Hz, 1H, Ar-H), 6.85 (s, 1H, Ar-H), 3.66 (s, 3H, CH_3_). ^13^C NMR (101 MHz, DMSO-*d_6_*): *δ* 140.0, 136.7, 131.0, 128.9, 128.5, 124.0, 122.4, 120.3, 119.1, 112.5, 100.6, 34.0. MS (ESI): 230 (M + H^+^, 100). Anal calcd for C_12_H_11_N_3_S: C, 62.86; H, 4.84; N, 18.33; S, 13.98. Found C, 63.10; H, 5.07; N, 18.05; S, 13.61.

2-((1*H*-indol-3-yl)thio)-1,3,4-thiadiazole (**6am**). Yellow amorphous solid. ^1^H NMR (400 MHz, DMSO-*d_6_*): *δ* 11.98 (s, 1H, NH), 9.29 (s, 1H, Ar-H), 8.00 (d, *J* = 2.8 Hz, 1H, Ar-H), 7.56 (d, *J* = 8.0 Hz, 1H, Ar-H), 7.53 (d, *J* = 7.9 Hz, 1H, Ar-H), 7.25 (dt, *J* = 1.0, 8.0 Hz, 1H, Ar-H), 7.16 (d, *J* = 7.9 Hz, 1H, Ar-H). ^13^C NMR (101 MHz, DMSO-*d_6_*): *δ* 173.0, 154.3, 137.2, 133.6, 127.8, 123.2, 121.3, 118.4, 113.2, 98.6. MS (ESI): 234 (M + H^+^, 100). Anal calcd for C_10_H_7_N_3_S_2_: C, 51.48; H, 3.02; N, 18.01; S, 27.48. Found C, 51.83; H, 3.39; N, 17.85; S, 27.17.

2-((1*H*-indol-3-yl)thio)-5-methyl-1,3,4-thiadiazole (**6an**). Yellow amorphous solid. ^1^H NMR (400 MHz, DMSO-*d_6_*): *δ* 11.95 (s, 1H, NH), 7.96 (d, *J* = 2.7 Hz, 1H, Ar-H), 7.55 (d, *J* = 2.7 Hz, 1H, Ar-H), 7.53 (d, *J* = 2.7 Hz, 1H, Ar-H), 7.24 (d, *J* = 7.5 Hz, 1H, Ar-H), 7.16 (d, *J* = 7.5 Hz, 1H, Ar-H), 2.50 (s, 3H, CH_3_). ^13^C NMR (101 MHz, CDCl_3_): *δ* 172.4, 165.7, 137.1, 133.5, 128.0, 123.1, 121.3, 118.4, 113.1, 98.8, 15.6. MS (ESI): 248 (M + H^+^, 100). Anal calcd for C_11_H_9_N_3_S_2_: C, 53.42; H, 3.67; N, 16.99; S, 25.92. Found C, 53.76; H, 3.92; N, 16.84; S, 25.59.

3-((1-Methyl-1*H*-tetrazol-5-yl)thio)-1*H-*indole (**6ao**). Pink amorphous solid. ^1^H NMR (400 MHz, DMSO-*d_6_*): *δ* 11.85 (s, 1H, NH), 7.94 (d, *J* = 2.7 Hz, 1H, Ar-H), 7.54 (d, *J* = 8.7 Hz, 1H, Ar-H), 7.51 (d, *J* = 8.7 Hz, 1H, Ar-H), 7.22 (d, *J* = 7.2 Hz, 1H, Ar-H), 7.13 (d, *J* = 7.2 Hz, 1H, Ar-H), 4.03 (s, 3H, NCH_3_). ^13^C NMR (101 MHz, DMSO-*d_6_*): *δ* 153.8, 136.9, 133.5, 128.8, 122.9, 121.0, 118.6, 112.9, 94.5, 34.5. MS (ESI): 232 (M + H^+^, 100). Anal calcd for C_10_H_9_N_5_S: C, 51.93; H, 3.92; N, 30.28; S, 13.86. Found C, 52.20; H, 4.28; N, 29.91; S, 13.93.

3-Thiocyanato-1*H-*indole (**7a**). White amorphous solid. ^1^H NMR (400 MHz, CDCl_3_): *δ* 8.76 (s, 1H, NH), 7.83 (dd, *J* = 5.9, 3.1 Hz, 1H, Ar-H), 7.52 (d, *J* = 2.8 Hz, 1H, Ar-H), 7.45 (dt, *J* = 5.9, 3.1 Hz, 1H, Ar-H), 7.35 (t, *J* = 3.1 Hz, 1H, Ar-H), 7.33 (t, *J* = 3.1 Hz, 1H, Ar-H). ^13^C NMR (101 MHz, CDCl_3_): *δ* 136.0, 131.0, 127.7, 123.9, 121.9, 118.8, 112.1, 111.9, 92.3. MS (ESI): 175 (M + H^+^, 100). These assignments matched with those previously published [109].

5-Chloro-3-thiocyanato-1-*H*-indole (**7b**). Yellow amorphous solid. ^1^H NMR (400 MHz, DMSO-*d_6_*): *δ* 12.20 (s, 1H, NH), 8.05 (s, 1H, Ar-H), 7.66 (s, 1H, Ar-H), 7.55 (d, *J* = 7.7 Hz, 1H, Ar-H), 7.26 (d, *J* = 7.7 Hz, 1H, Ar-H). ^13^C NMR (101 MHz, DMSO-*d_6_*): *δ* 135.3, 135.2, 129.1, 126.4, 123.5, 117.4, 115.0, 112.5, 89.9. MS (ESI): 209 (M + H^+^, 100), 211 (M + H^+^, 32). These assignments matched with those previously published [110].

5-Methyl-3-thiocyanato-1-*H*-indole (**7c**). White amorphous solid. ^1^H NMR (400 MHz, DMSO-*d_6_*): *δ* 12.00 (s, 1H, NH), 7.94 (d, *J* = 2.9 Hz, 1H, Ar-H), 7.35 (d, *J* = 8.2 Hz, 1H, Ar-H), 7.12 (t, *J* = 8.2 Hz, 1H, Ar-H), 6.93 (d, *J* = 7.1 Hz, 1H, Ar-H), 2.85 (s, 3H, CH_3_). ^13^C NMR (101 MHz, DMSO-*d_6_*): *δ* 137.2, 134.6, 129.9, 125.7, 123.4, 123.0, 114.3, 111.3, 89.8, 19.2. MS (ESI): 189 (M + H^+^, 100). These assignments matched with those previously published [111].

5-Methoxy-3-thiocyanato-1*H-*indole (**7d**). White amorphous solid. ^1^H NMR (400 MHz, CDCl_3_): *δ* 8.55 (s, 1H, NH), 7.67 (d, *J* = 8.7 Hz, 1H, Ar-H), 7.40 (d, *J* = 2.7 Hz, 1H, Ar-H), 6.97 (dd, *J* = 8.7, 2.1 Hz, 1H, Ar-H), 6.90 (d, *J* = 2.1 Hz, 1H, Ar-H), 3.86 (s, 3H, OCH_3_). ^13^C NMR (101 MHz, CDCl_3_): *δ* 157.7, 136.9, 129.8, 121.8, 119.5, 112.1, 111.9, 95.2, 92.3, 55.7. MS (ESI): 205 (M + H^+^, 100). These assignments matched with those previously published [109].

3-Thiocyanato-1-*H*-indole-5-carbonitrile (**7e**). Yellow amorphous solid. ^1^H NMR (400 MHz, DMSO-*d_6_*): *δ* 12.52 (s, 1H, NH), 8.23 (d, *J* = 2.6 Hz, 1H, Ar-H), 8.21 (s, 1H, Ar-H), 7.71 (d, *J* = 8.5 Hz, 1H, Ar-H), 7.64 (dd, *J* = 8.6, 2.6 Hz, 1H, Ar-H). ^13^C NMR (101 MHz, DMSO-*d_6_*): *δ* 143.4, 141.1, 132.5, 130.9, 128.6, 125.1, 119.5, 117.2, 108.7, 96.8. MS (ESI): 200 (M + H^+^, 100). These assignments matched with those previously published [110].

5-Nitro-3-thiocyanato-1-*H*-indole (**7f**). Yellow amorphous solid. ^1^H NMR (400 MHz, DMSO-*d_6_*): *δ* 12.63 (s, 1H, NH), 8.51 (d, *J* = 1.8 Hz, 1H, Ar-H), 8.27 (s, 1H, Ar-H), 8.12 (dd, *J* = 9.0, 1.8 Hz, 1H, Ar-H), 7.70 (d, *J* = 9.0 Hz, 1H, Ar-H). ^13^C NMR (101 MHz, DMSO-*d_6_*): *δ* 142.6, 140.0, 137.5, 127.3, 118.6, 114.8, 114.1, 112.4, 93.6. MS (ESI): 220 (M + H^+^, 100). These assignments matched with those previously published [110].

4-Methyl-3-thiocyanato-1-*H*-indole (**7g**). White amorphous solid. ^1^H NMR (400 MHz, DMSO-*d_6_*): *δ* 11.91 (s, 1H, NH), 7.92 (d, *J* = 2.9 Hz, 1H, Ar-H), 7.46 (s, 1H, Ar-H), 7.43 (d, *J* = 8.3 Hz, 1H, Ar-H), 7.10 (dd, *J* = 8.3, 1.1 Hz, 1H, Ar-H), 2.45 (s, 3H, CH_3_). ^13^C NMR (101 MHz, DMSO-*d_6_*): *δ* 135.1, 133.5, 130.5, 128.2, 125.0, 117.7, 113.0, 112.8, 89.0, 21.6. MS (ESI): 189 (M + H^+^, 100). These assignments matched with those previously published [110].

3-Thiocyanato-1*H-*pyrrolo[2,3-*b*]pyridine (**7h**). White amorphous solid. ^1^H NMR (400 MHz, DMSO): *δ* 12.62 (s, 1H, NH), 8.40 (d, *J* = 4.5 Hz, 1H, Ar-H), 8.18 (s, 1H, Ar-H), 8.13 (d, *J* = 7.8 Hz, 1H, Ar-H), 7.31 (dd, *J* = 7.8, 4.7 Hz, 1H, Ar-H). ^13^C NMR (101 MHz, DMSO): *δ* 148.8, 145.0, 134.4, 127.0, 120.3, 117.8, 112.6, 89.5. MS (ESI): 176 (M + H^+^, 100). These assignments matched with those previously published [112].

3-(Phenylselanyl)-1*H*-indole (**9a**). Yellow amorphous solid. ^1^H NMR (400 MHz, CDCl_3_): *δ* 8.43 (s, 1H, NH), 7.69 (d, *J* = 7.9 Hz, 1H, Ar-H), 7.49 (d, *J* = 2.5 Hz, 1H, Ar-H), 7.46 (d, *J* = 8.2 Hz, 1H, Ar-H), 7.33–7.27 (m, 3H, Ar-H), 7.24–7.12 (m, 4H, Ar-H). ^13^C NMR (101 MHz, CDCl_3_): *δ* 136.4, 133.9, 131.3, 130.0, 129.0, 128.7, 125.6, 123.0, 120.9, 120.4, 111.4, 98.2. MS (ESI): 274 (M + H^+^, 100). These assignments matched with those previously published [30].

2-Methyl-3-(phenylselanyl)-1*H*-indole (**9b**). Yellow amorphous solid. ^1^H NMR (400 MHz, CDCl_3_): *δ* 8.20 (s, 1H, NH), 7.64 (d, *J* = 7.7 Hz, 1H, Ar-H), 7.36 (d, *J* = 7.9 Hz, 1H, Ar-H), 7.26 (dd, *J* = 3.5, 1.4 Hz, 1H, Ar-H), 7.23 (dd, *J* = 3.5, 1.4 Hz, 2H, Ar-H), 7.20 (d, *J* = 4.1 Hz, 1H, Ar-H), 7.19–7.12 (m, 3H, Ar-H), 2.56 (s, 3H, CH_3_). ^13^C NMR (101 MHz, CDCl_3_): *δ* 141.0, 135.8, 134.0, 131.3, 129.0, 128.4, 125.5, 122.2, 120.7, 119.8, 110.6, 96.2, 13.2. MS (ESI): 288 (M + H^+^, 100). These assignments matched with those previously published [113].

5-Methyl-3-(phenylselanyl)-1*H*-indole (**9c**). Yellow amorphous solid. ^1^H NMR (400 MHz, CDCl_3_): *δ* 8.30 (s, 1H, NH), 7.51 (d, *J* = 0.5 Hz, 1H, Ar-H), 7.44 (d, *J* = 2.5 Hz, 1H, Ar-H), 7.35 (d, *J* = 8.3 Hz, 1H, Ar-H), 7.31–7.28 (m, 2H, Ar-H), 7.22–7.14 (m, 4H, Ar-H), 2.49 (s, 3H, CH_3_). ^13^C NMR (101 MHz, CDCl_3_): *δ* 134.7, 134.1, 131.5, 130.4, 130.3, 129.0, 128.6, 125.6, 124.7, 119.9, 111.1, 97.4, 21.5. MS (ESI): 288 (M + H^+^, 100). These assignments matched with those previously published [32].

5-Methoxy-3-(phenylselanyl)-1*H*-indole (**9d**). Yellow amorphous solid. ^1^H NMR (400 MHz, CDCl_3_): *δ* 8.42 (s, 1H, NH), 7.44 (d, *J* = 2.5 Hz, 1H, Ar-H), 7.33 (d, *J* = 8.8 Hz, 1H, Ar-H), 7.30 (dd, *J* = 8.2, 1.5 Hz, 1H, Ar-H), 7.29 (s, 1H, Ar-H), 7.21–7.13 (m, 4H, Ar-H), 6.97 (dd, *J* = 8.8, 2.5 Hz, 1H, Ar-H), 3.85 (s, 3H, OCH_3_). ^13^C NMR (101 MHz, CDCl_3_): *δ* 155.1, 134.0, 132.0, 131.4, 130.8, 129.1, 128.5, 125.6, 113.5, 112.4, 101.6, 97.5, 55.9. MS (ESI): 304 (M + H^+^, 100). These assignments matched with those previously published [113].

## 4. Conclusions

In summary, we have developed a practical GO-promoted and transition metal-free light induced methodology for the construction of a carbon-chalcogen (S and Se) bond that provides 3-chalcogenyl indoles in good to excellent yields under open air. The key features of this simple and robust protocol are: (1) metal-free and iodine-free conditions; (2) easy-to-handle oxidant; (3) open to the air; (4) atom-economic; (5) performed on a gram-scale; (6) regioselective; and (7) applicable to different sources of organochalcogenides with substituted indoles for this transformation. Moreover, very few methods report the combination of GO and light which works in synergy to efficiently promote the organic reactions [83].

## Figures and Tables

**Table 1:**
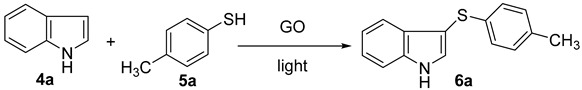
Optimization of the reaction conditions *^a^*.

Entry	GO (wt %) *^b^*	Light Source	Solvent	Yield (%) *^c^*
1	40	Sunlight	CH_3_CN	28
2	40	22 W CWF bulb	CH_3_CN	22
3	40	1W Green LED	CH_3_CN	7
4	40	3W blue LED	CH_3_CN	61
5	40	No light	CH_3_CN	0
6	40	3W blue LED	THF	5
7	40	3W blue LED	DMSO	7
8	40	3W blue LED	Toluene	34
9	40	3W blue LED	DCE	78
10	40	3W blue LED	DMF	0
11	40	3W blue LED	1,4-Dioxane	0
12	20	3W blue LED	DCE	67
13	30	3W blue LED	DCE	72
14	50	3W blue LED	DCE	87
15	60	3W blue LED	DCE	85
16	0	3W blue LED	DCE	0
17	50	No light	DCE	<5

*^a^* Reaction conditions: **4a** (0.3 mmol), **5a** (0.36 mmol), and solvent (1 mL), for 12 h at rt under open air. *^b^* With respect to the substrate **4a**. *^c^* Isolated yield.

**Table 2:**
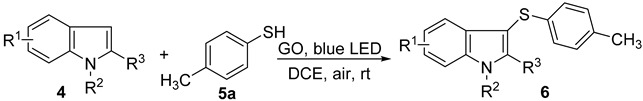
Scope of indoles *^a^*.

Entry	R^1^	R^2^	R^3^	Product	Yield (%) *^b^*
1	H	H	H	**6aa**	87
2	5-I	H	H	**6ba**	83
3	5-CH_3_	H	H	**6ca**	89
4	5-CN	H	H	**6da**	78
5	6-OCH_3_	H	H	**6ea**	90
6	7-Cl	H	H	**6fa**	80
7	7-OBn	H	H	**6ga**	83
8	4-CH_3_	H	H	**6ha**	71
9	4-CO_2_CH_3_	H	H	**6ia**	67
10	H	H	2-CH_3_	**6ja**	86
11	H	CH_3_	H	**6ka**	86
12	5-CH_3_	H	2-CH_3_	**6la**	82
13 *^c^*	H	H	3-CH_3_	**6ma**	84

*^a^* Reaction conditions: **4** (0.3 mmol), **5a** (0.36 mmol), GO (50 wt %) with respect to the substrate **4a**, and DCE (1 mL), for 12 h at rt under open air. *^b^* Isolated yield. *^c^* 3-Methyl-2-(*p*-tolylthio)-1*H*- indole (**6ma**) was obtained.

**Table 3:**
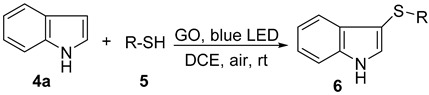
Scope of thiols *^a^*.

Entry	R	Product	Yield (%) *^b^*
1	4-ClPh	**6ab**	86
2	4-BrPh	**6ac**	88
3	4-OCH_3_Ph	**6ad**	83
4	4-C_2_H_5_Ph	**6ae**	80
5	2,4-dimethylphenyl	**6af**	82
6	4-NO_2_Ph	**6ag**	91
7	Naphthalen-2-yl	**6ah**	78
8 *^c^*	4-OCH_3_Ph	**6ai**	78
9 *^d^*	4-CH_3_	**6aj**	76

*^a^* Reaction conditions: **4a** (0.3 mmol), **5** (0.36 mmol), GO (50 wt %) with respect to the substrate **4a**, and DCE (1 mL), for 12 h at rt under open air. *^b^* Isolated yield. *^c^* 5-Methoxy-3-((4-methoxyphenyl)thio)-1*H*-indole (**6ai**) was obtained. *^d^* 3-(*p*-Tolylthio)-1*H*-pyrrolo[3,2-*b*]pyridine (**6aj**) was obtained.

## Data Availability

Data is contained within the article and Supplementary Material.

## Supplementary Materials

The following are available online, Figure S1: (A) SEM image of graphite. (B) SEM image of GO, Figure S2: TEM image of graphite, Figure S3: TEM image of GO, Figure S4: AFM image of GO, Figure S5: Raman image of GO I D /I G ratio = 0.87, Figure S6: XRD image of GO.

## Sample Availability

Samples of the compounds **4**, **5**, and **8** are available from the authors.

## Electronic Supplementary Information (ESI) Available

Experimental procedures and characterization data, characterization of GO, and ^1^H and ^13^C NMR spectra of compounds **6**, **7** and **9**.
