# Supplementary material for: Visible-Light-Induced, Graphene Oxide-Promoted C3-Chalcogenylation of Indoles Strategy under Transition-Metal-Free Conditions
Source: Molecules. 2022 Jan 25;27(3):772. doi: 10.3390/molecules27030772 (PMC8839487; doi:10.3390/molecules27030772)
Supplement: Supplementary file 1 [file molecules-27-00772-s001.zip › molecules-1530903-sup-revised-╥╤╫¬╗╗.pdf]

# Supporting Information for

# Visible-Light-Induced, Graphene Oxide-Promoted C3-chalcogenylation of Indoles Strategy under Transition-Metal-Free Conditions

*Qing Huang,[a] Xiangjun Peng,*[,b] Hong Li,[a] Haiping He,[b] Liangxian Liu*[,a]*

*[a] Department of Chemistry and Chemical Engineering, Gannan Normal University, Ganzhou, Jiangxi 341000, P. R. China*

*[b] School of Pharmaceutical Science, Gannan Medical University, Ganzhou, Jiangxi 341000, P. R. China*

*E-mail: liuliangxian1962@163.com*

**CONTENTS**

1. General Methods .......................................................................................................... 2

2. Characterization of **GO**..........................................................................................................2

3. General Procedure and Spectroscopic Data of the Products **6**, **7** and **9**..................................... 4

4. Copies of $^{1}$H and $^{13}$C Spectra. ..........................................................................................15

5. References..........................................................................................................................54

## 1. General Methods

$^1$H and $^{13}$C NMR spectra were recorded on a Bruker spectrometers at 400 and 101 MHz, respectively. Mass spectra were recorded with Bruker Dalton Esquire 3000 plus LC-MS apparatus. Elemental analysis were carried out on a Perkin-Elmer 240B instrument. Silica gel (300-400 mesh) was used for flash column chromatography, eluting (unless otherwise stated) with an ethyl acetate/petroleum ether (PE) (60-90 °C) mixture.

Raman spectra were collected with a Horiba Jobin Y von-Labram HR UV-Visible-NIR Raman Microscope Spectrometer, using a 632 nm laser. The spectra were the average of 10 scans at a resolution of 2 cm$^{-1}$ between 1000-2000 cm$^{-1}$ Raman Shift.

## 2. Characterization of GO

GO was prepared by graphite oxidation using the Hummers and Offeman method and subsequent exfoliation. Further details and GO characterization have been previously reported.

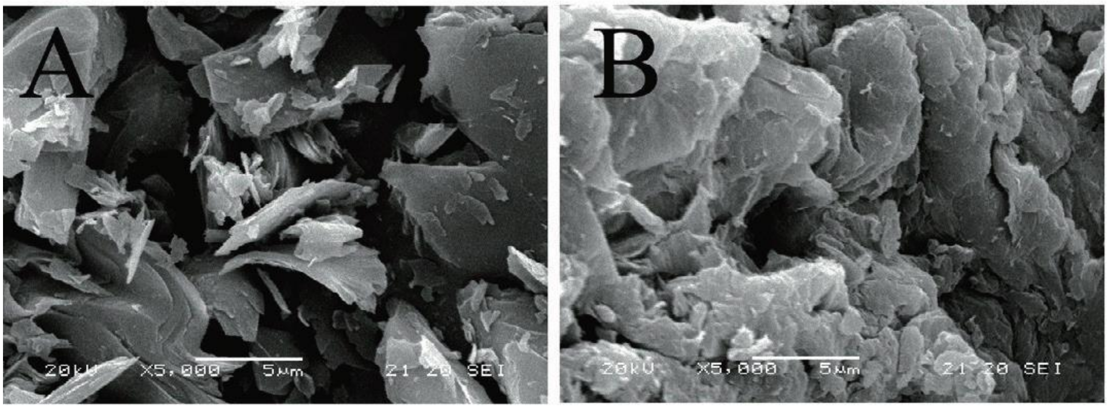


**Figure S1**. (A) SEM image of graphite. (B) SEM image of GO.

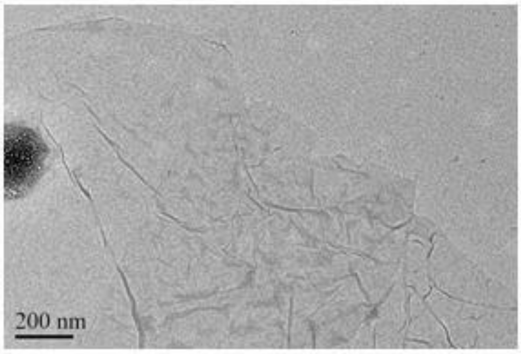


**Figure S2**. TEM image of graphite.

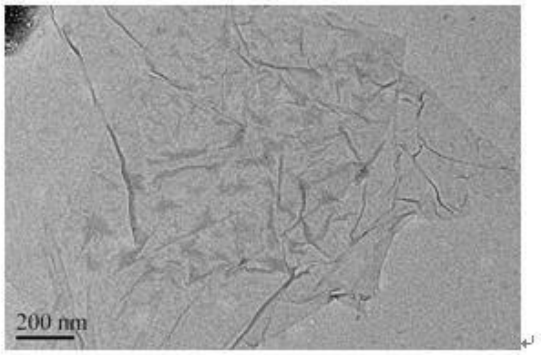


**Figure S3**. TEM image of GO.

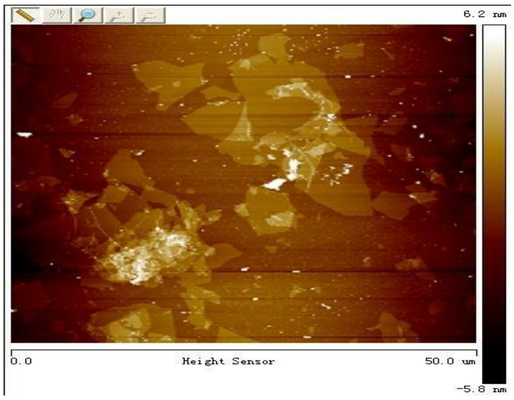


**Figure S4**. AFM image of GO.

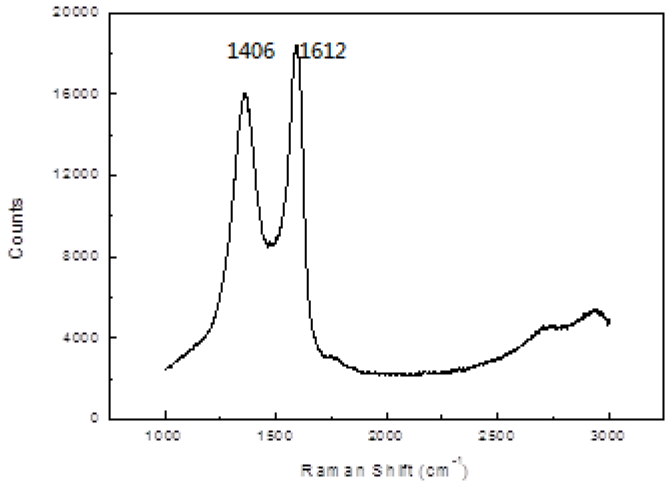


**Figure S5**. Raman image of GO ($I_D/I_G$ ratio = 0.87).

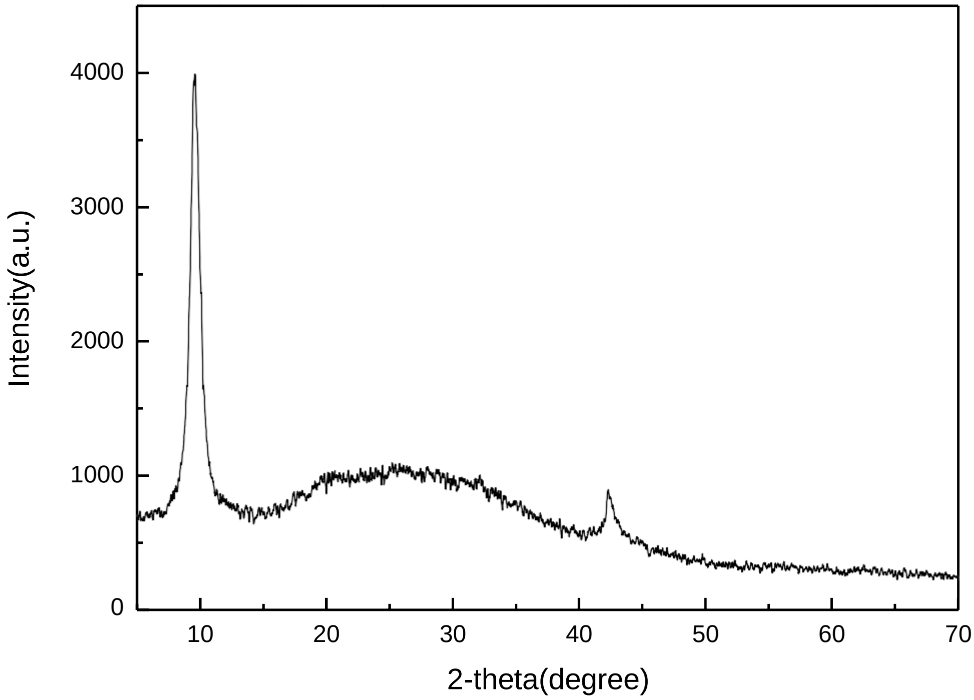


**Figure S6**. XRD image of GO.

**3. General Procedure and Spectroscopic Data of the Products 6**

In a 10 mL Schlenk tube, indole (0.3 mmol), GO (17.6 mg), and thiol (0.36 mmol) were stirred in DCE (1 mL) for 12 h at room temperature under an air atmosphere irradiated by blue LEDs. The reaction mixture was concentrated under reduced pressure. The residue was purified by flash chromatography on silica gel (eluent: EtOAc/PE = 1:10) to yield the corresponding product **6**.

3-(*p*-Tolylthio)-1*H*-indole (**6aa**)

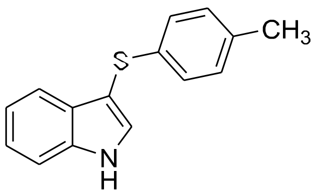


Yellow amorphous solid. $^1$H NMR (400 MHz, $CDCl_3$): $\delta$ 8.37 (s, 1H), 7.63 (d, $J$ = 7.9 Hz, 1H), 7.46 (d, $J$ = 2.6 Hz, 1H), 7.43 (d, $J$ = 8.1 Hz, 1H), 7.26 (dt, $J$ = 1.0, 8.1 Hz, 1H, Ar-H), 7.18 (t, $J$ = 7.1 Hz, 1H), 7.05 (d, $J$ = 8.3 Hz, 2H), 6.99 (d, $J$ = 8.3 Hz, 2H), 2.26 (s, 3H). $^{13}$C NMR (101 MHz, $CDCl_3$): $\delta$ 136.5, 135.5, 134.7, 130.4, 129.5, 129.1, 126.3, 123.0, 120.8, 119.7, 111.5, 103.6, 20.8. MS (ESI): 240 (M+H$^+$, 100). These assignments matched with those previously published.[1]

5-Iodo-3-(*p*-tolylthio)-1*H*-indole (**6ba**)

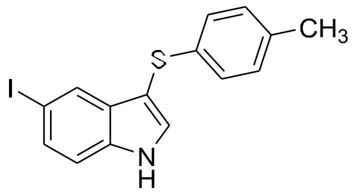


Brown amorphous solid. $^1$H NMR (400 MHz, $CDCl_3$): $\delta$ 8.51 (s, 1H, NH), 7.99 (d, $J$ = 1.5 Hz, 1H, Ar-H), 7.53 (dd, $J$ = 8.5, 1.5 Hz, 1H, Ar-H), 7.43 (d, $J$ = 1.5 Hz, 1H, Ar-H), 7.21 (d, $J$ = 8.5 Hz, 1H, Ar-H), 7.06-7.01 (m, 4H, Ar-H), 2.29 (s, 3H, $CH_3$). $^{13}$C NMR (101 MHz, $CDCl_3$): $\delta$ 135.6, 135.1, 134.9, 131.7, 131.4, 131.3, 129.6, 128.4, 126.3, 113.6, 102.8, 84.6, 20.9. MS (ESI): 366 (M+H$^+$, 100). These assignments matched with those previously published.[2]

5-Methyl-3-(*p*-tolylthio)-1*H*-indole (**6ca**)

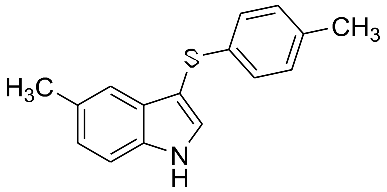


Yellow amorphous solid. $^1$H NMR (400 MHz, $CDCl_3$): $\delta$ 8.19 (s, 1H, NH), 7.55 (d, $J$ = 0.7 Hz, 1H, Ar-H), 7.40 (d, $J$ = 2.6 Hz, 1H, Ar-H), 7.34 (d, $J$ = 8.2 Hz, 1H, Ar-H), 7.21-7.13 (m, 3H, Ar-H), 7.08 (d, $J$ = 8.2 Hz, 2H, Ar-H), 2.52 (s, 3H, $CH_3$), 2.36 (s, 3H, $CH_3$). $^{13}$C NMR (101 MHz, $CDCl_3$): $\delta$ 135.9, 134.9, 134.7, 130.94, 130.4, 129.7, 129.5, 126.2, 124.7, 119.2, 111.5, 102.4, 21.6, 21.0. MS (ESI): 254 (M+H$^+$, 100). These assignments matched with those previously published.[3]

3-(*p*-Tolylthio)-1*H*-indole-5-carbonitrile (**6da**)

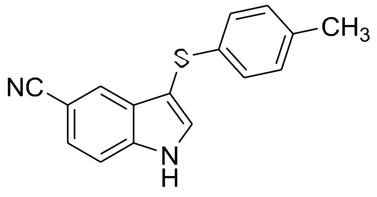

Yellow amorphous solid. $^1$H NMR (400 MHz, DMSO-$d_6$): $\delta$ 12.20 (s, 1H, NH), 7.99 (d, $J$ = 1.6 Hz, 1H, Ar-H), 7.81 (s, 1H, Ar-H), 7.66 (d, $J$ = 8.4 Hz, 1H, Ar-H), 7.53 (d, $J$ = 8.4 Hz, 1H, Ar-H), 7.04 (d, $J$ = 8.0 Hz, 2H, Ar-H), 6.99 (d, $J$ = 8.0 Hz, 2H, Ar-H), 2.20 (s, 3H, $CH_3$). $^{13}$C NMR (101 MHz, DMSO-$d_6$): $\delta$ 139.1, 135.3, 135.2, 134.9, 130.1, 129.0, 126.9, 125.4, 124.1, 120.7, 114.3, 102.8, 102.5, 20.9. MS (ESI): 265 (M+H$^+$, 100). These assignments matched with those previously published.[3]

6-Methoxy-3-(*p*-tolylthio)-1*H*-indole (**6ea**)

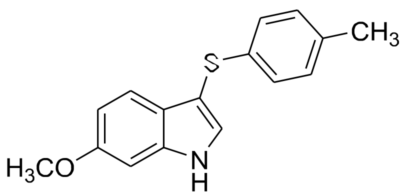


Reddish brown amorphous solid. $^1$H NMR (400 MHz, $CDCl_3$): $\delta$ 8.33 (s, 1H, NH), 7.51 (d, $J$ = 8.6 Hz, 1H, Ar-H), 7.34 (d, $J$ = 2.2 Hz, 1H, Ar-H), 7.08 (d, $J$ = 8.2 Hz, 2H, Ar-H), 7.02 (d, $J$ = 8.2 Hz, 2H, Ar-H), 6.90 (d, $J$ = 2.2 Hz, 1H, Ar-H), 6.86 (dd, $J$ = 8.6, 2.2 Hz, 1H, Ar-H), 3.87 (s, 3H, $OCH_3$), 2.29 (s, 3H, $CH_3$). $^{13}$C NMR (101 MHz, $CDCl_3$): $\delta$ 157.2, 137.3, 135.6, 134.7, 129.5, 129.3, 126.3, 123.3, 120.3, 110.8, 103.4, 95.2, 55.7, 20.9. MS (ESI): 270 (M+H$^+$, 100). These assignments matched with those previously published.[4]

7-Chloro-3-(*p*-tolylthio)-1*H*-indole (**6fa**)

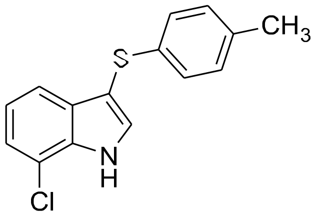


Red amorphous solid. $^1$H NMR (400 MHz, $CDCl_3$): $\delta$ 8.65 (s, 1H, NH), 7.55 (dt, $J$ = 1.0, 7.8 Hz, 1H, Ar-H), 7.54 (d, $J$ = 2.6 Hz, 1H, Ar-H), 7.29 (dd, $J$ = 7.8, 1.0 Hz, 1H, Ar-H), 7.12 (t, $J$ = 7.8 Hz, 1H, Ar-H), 7.08 (d, $J$ = 8.2 Hz, 2H, Ar-H), 7.03 (d, $J$ = 8.2 Hz, 2H, Ar-H), 2.30 (s, 3H, $CH_3$). $^{13}$C NMR (101 MHz, $CDCl_3$): $\delta$ 135.0, 134.9, 133.8, 130.9, 130.6, 129.6 , 126.6, 122.4, 121.6, 118.4, 117.0, 105.2, 20.9. MS (ESI): 274 (M+H$^+$, 30), 276 (M+H$^+$, 100). Anal calcd for $C_{15}H_{12}ClNS$: C, 65.81; H, 4.42; N, 5.12; S, 11.71. Found C, 65.46; H, 4.81; N, 4.83; S, 11.62.

7-(Benzyloxy)-3-(*p*-tolylthio)-1*H*-indole (**6ga**)

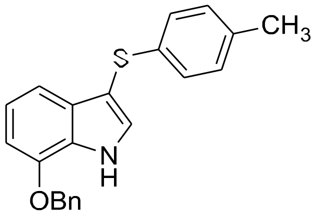


Reddish brown amorphous solid. $^1$H NMR (400 MHz, $CDCl_3$): $\delta$ 8.71 (s, 1H, NH), 7.52 (d, $J$ = 7.1 Hz, 2H, Ar-H), 7.49-7.40 (m, 4H, Ar-H), 7.27 (d, $J$ = 8.4 Hz, 1H, Ar-H), 7.09 (t, $J$ = 7.8 Hz, 1H, Ar-H), 7.07 (d, $J$ = 8.0 Hz, 2H, Ar-H), 7.01 (d, $J$ = 8.0 Hz, 2H, Ar-H), 6.81 (d, $J$ = 7.8 Hz, 1H, Ar-H), 5.24 (s, 2H, $OCH_2$), 2.28 (s, 3H, $CH_3$). $^{13}$C NMR (101 MHz, $CDCl_3$): $\delta$ 145.6, 136.9, 135.7, 134.6, 130.8, 130.1, 129.5, 128.7, 128.3, 128.0, 127.2, 126.3, 121.2, 112.6, 104.0 , 103.7, 70.4,

20.9. MS (ESI): 346 (M+H$^+$, 100). Anal calcd for $C_{22}H_{19}NOS$: C, 76.49; H, 5.54; N, 4.05; S, 9.28. Found C, 76.35; H, 5.47; N, 4.33; S, 8.95.

4-Methyl-3-(*p*-tolylthio)-1*H*-indole (**6ha**)

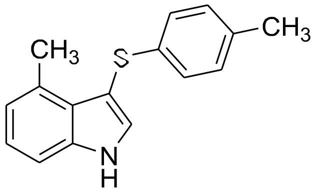


Reddish brown amorphous solid. $^1$H NMR (400 MHz, $CDCl_3$) $\delta$ 8.41 (s, 1H, NH), 7.43 (d, *J* = 2.6 Hz, 1H, Ar-H), 7.29 (d, *J* = 8.1 Hz, 1H, Ar-H), 7.17 (t, *J* = 8.1 Hz, 1H, Ar-H), 7.06-6.98 (m, 4H, Ar-H), 6.92 (d, *J* = 7.1 Hz, 1H), 2.70 (s, 3H, $CH_3$), 2.29 (s, 3H, $CH_3$). $^{13}$C NMR (101 MHz, $CDCl_3$): $\delta$ 137.9, 137.0, 134.3, 132.2, 131.7, 129.6, 127.0, 125.5, 123.1, 122.4, 109.4, 102.9, 20.9, 18.7. MS (ESI): 254 (M+H$^+$, 100). These assignments matched with those previously published.[3]

Methyl 3-(*p*-tolylthio)-1*H*-indole-4-carboxylate (**6ia**)

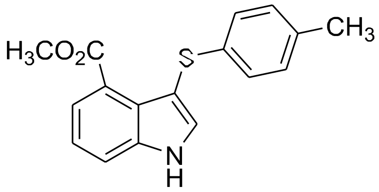


Brown amorphous solid. $^1$H NMR (400 MHz, $CDCl_3$): $\delta$ 9.19 (s, 1H, NH), 7.51 (d, *J* = 2.6 Hz, 1H, Ar-H), 7.49 (d, *J* = 1.0 Hz, 1H, Ar-H), 7.38 (d, *J* = 2.6 Hz, 1H, Ar-H), 7.24 (t, *J* = 7.8 Hz, 1H, Ar-H), 6.98 (s, 4H, Ar-H), 3.68 (s, 3H), 2.30 (d, *J* = 37.7 Hz, 3H). $^{13}$C NMR (101 MHz, $CDCl_3$): $\delta$ 169.6, 137.6, 136.4, 134.6, 133.4, 129.4, 126.2, 125.4, 125.3, 122.1, 122.0, 115.1, 103.1, 51.9, 20.8. MS (ESI): 298 (M+H$^+$, 100). Anal calcd for $C_{17}H_{15}NO_2S$: C, 68.66; H, 5.08; N, 4.71; S, 10.78. Found C, 68.80; H, 5.26; N, 4.64; S, 10.57.

2-Methyl-3-(*p*-tolylthio)-1*H*-indole (**6ja**)

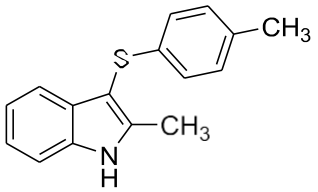


Reddish brown amorphous solid. $^1$H NMR (400 MHz, $CDCl_3$): $\delta$ 8.26 (s, 1H, NH), 7.58 (d, *J* = 7.8 Hz, 1H, Ar-H), 7.36 (dt, *J* = 1.0, 7.8 Hz, 1H, Ar-H), 7.22 (dt, *J* = 1,0, 7.8 Hz, 1H, Ar-H), 7.15 (dt, *J* = 1.0, 7.8 Hz, 1H, Ar-H), 6.99 (s, 4H, Ar-H), 2.54 (s, 3H, $CH_3$), 2.27 (s, 3H, $CH_3$). $^{13}$C NMR (101 MHz, $CDCl_3$): $\delta$ 141.0, 135.7, 135.5, 134.4, 130.4, 129.5, 125.8 , 122.1, 120.7, 119.0, 110.7, 99.9, 20.9, 12.2. MS (ESI): 254 (M+H$^+$, 100). These assignments matched with those previously published.[3]

1-Methyl-3-(*p*-tolylthio)-1*H*-indole (**6ka**)

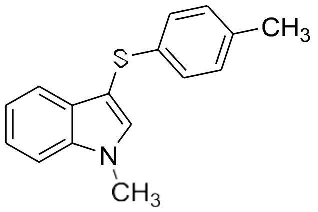

Reddish brown amorphous solid. $^1$H NMR (400 MHz, $CDCl_3$): $\delta$ 7.75 (d, $J$ = 8.2 Hz, 1H, Ar-H), 7.45 (d, $J$ = 8.2 Hz, 1H, Ar-H), 7.39 (dt, $J$ = 1.0, 7.0 Hz, 1H, Ar-H), 7.37 (s, 1H, Ar-H), 7.28 (dt, $J$ = 1.0, 7.0 Hz, 1H, Ar-H), 7.15 (d, $J$ = 8.2 Hz, 2H, Ar-H), 7.07 (d, $J$ = 8.2 Hz, 1H, Ar-H), 3.86 (s, 3H, $NCH_3$), 2.35 (s, 3H, $CH_3$). $^{13}$C NMR (101 MHz, $CDCl_3$): $\delta$ 137.6, 136.1, 134.9, 134.6, 123.0, 129.6, 126.3, 126.2, 122.6, 120.5, 119.8, 109.8, 101.3, 33.1, 21.0. MS (ESI): 254 ($M+H^+$, 100). These assignments matched with those previously published.[2]

2,5-Dimethyl-3-(*p*-tolylthio)-1*H*-indole (**6la**)

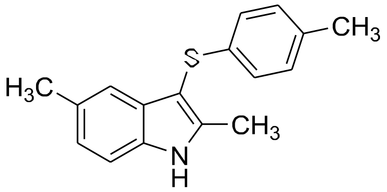


Reddish brown amorphous solid. $^1$H NMR (400 MHz, $CDCl_3$): $\delta$ 8.03 (s, 1H, NH), 7.46 (s, 1H, Ar-H), 7.25 (d, $J$ = 8.2 Hz, 1H, Ar-H), 7.10 (dd, $J$ = 8.2, 1.2 Hz, 1H, Ar-H), 7.46 (s, 4H, Ar-H), 2.51 (s, 3H, $CH_3$), 2.49 (s, 3H, $CH_3$), 2.34 (s, 3H, $CH_3$). $^{13}$C NMR (101 MHz, $CDCl_3$): $\delta$ 141.3, 136.0, 134.3, 133.8, 130.7, 130.1, 129.6, 125.7, 123.7, 118.7, 110.5, 99.0, 21.5, 20.9, 12.1. MS (ESI): 268 ($M+H^+$, 100). These assignments matched with those previously published.[3]

3-Methyl-2-(*p*-tolylthio)-1*H*-indole (**6ma**)

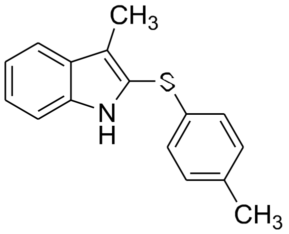


Reddish brown amorphous solid. $^1$H NMR (400 MHz, $CDCl_3$): $\delta$ 7.96 (s, 1H, NH), 7.71 (d, $J$ = 7.9 Hz, 1H, Ar-H), 7.33 (d, $J$ = 3.6 Hz, 2H, Ar-H), 7.26 (m, 1H, Ar-H), 7.12 (d, $J$ = 8.3 Hz, 2H, Ar-H), 7.09 (d, $J$ = 8.3 Hz, 2H, Ar-H), 2.51 (s, 3H, $CH_3$), 2.38 (s, 3H, $CH_3$). $^{13}$C NMR (101 MHz, $CDCl_3$): $\delta$ 136.9, 135.9, 133.5, 130.0, 128.6, 127.1, 123.4, 122.3, 119.7, 119.5, 119.4, 111.0, 21.0, 9.6. MS (ESI): 254 ($M+H^+$, 100). These assignments matched with those previously published.[3]

3-((4-Chlorophenyl)thio)-1*H*-indole (**6ab)**

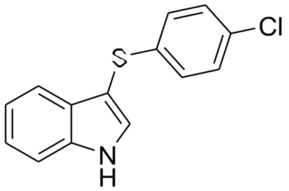


Light yellow amorphous solid. $^1$H NMR (400 MHz, $CDCl_3$): $\delta$ 8.47 (s, 1H, NH), 7.58 (d, $J$ = 8.0 Hz, 1H, Ar-H), 7.49 (d, $J$ = 2.6 Hz, 1H, Ar-H), 7.45 (d, $J$ = 8.2 Hz, 1H, Ar-H), 7.29 (dt, $J$ = 1.0, 8.0 Hz, 1H, Ar-H), 7.18 (dt, $J$ = 1.0, 8.0 Hz, 1H, Ar-H), 7.12 (d, $J$ = 8.7 Hz, 2H, Ar-H), 7.02 (d, $J$ = 8.7 Hz, 2H, Ar-H). $^{13}$C NMR (101 MHz, $CDCl_3$): $\delta$ 137.8, 136.5, 130.6, 130.5, 128.7, 128.6, 127.1, 123.1, 121.0, 119.4, 111.6, 102.4. MS (ESI): 260 ($M+H^+$, 30), 262 ($M+H^+$, 100). These assignments matched with those previously published.[2]

3-((4-Bromophenyl)thio)-1*H*-indole (**6ac**)

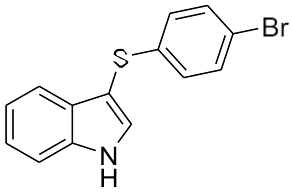


Brown amorphous solid. $^1$H NMR (400 MHz, DMSO-*d*$_6$): *δ* 11.76 (s, 1H, NH), 7.80 (d, *J* = 2.7 Hz, 1H, Ar-H), 7.51 (d, *J* = 8.1 Hz, 1H, Ar-H), 7.41 – 7.35 (m, 3H, Ar-H), 7.20 (dt, *J* = 1.1, 8.1 Hz, 1H, Ar-H), 7.08 (dt, *J* = 1.1, 8.1 Hz, 1H, Ar-H), 6.96 (dt, *J* = 2.7, 8.6 Hz, 2H, Ar-H). $^{13}$C NMR (101 MHz, DMSO-*d*$_6$): *δ* 139.5, 137.3, 133.1, 132.1, 128.9, 127.7, 122.7, 120.7, 118.6, 118.0, 112.9, 99.1. MS (ESI): 304 (M+H$^+$, 100), 306 (M+H$^+$, 100). These assignments matched with those previously published.[2]

3-((4-Methoxyphenyl)thio)-1*H*-indole (**6ad**)

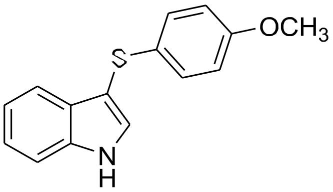


Brown amorphous solid. $^1$H NMR (400 MHz, $CDCl_3$): *δ* 8.38 (s, 1H, NH), 7.63 (d, *J* = 8.0 Hz, 1H, Ar-H), 7.46 (d, *J* = 2.6 Hz, 1H, Ar-H), 7.41 (d, *J* = 8.0 Hz, 1H, Ar-H), 7.25 (dt, *J* = 1.0, 8.0 Hz, 1H, Ar-H), 7.17 (dt, *J* = 1.0, 8.0 Hz, 1H, Ar-H), 7.13 (d, *J* = 8.9 Hz, 2H, Ar-H), 6.74 (d, *J* = 8.9 Hz, 2H, Ar-H), 3.73 (s, 3H, $OCH_3$). $^{13}$C NMR (101 MHz, $CDCl_3$): *δ* 157.8, 136.5, 123.0, 129.5, 129.0, 128.6, 122.9, 120.8, 119.7, 114.5, 111.5, 104.7, 55.3. MS (ESI): 256 (M+H$^+$, 100). These assignments matched with those previously published.[1]

3-((4-Ethylphenyl)thio)-1*H*-indole (**6ae**)

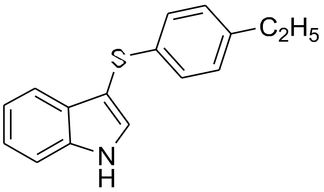


Reddish brown amorphous solid. $^1$H NMR (400 MHz, $CDCl_3$): *δ* 8.34 (s, 1H, NH), 7.71 (d, *J* = 8.0 Hz, 1H, Ar-H), 7.45 (d, *J* = 2.3 Hz, 1H, Ar-H), 7.44 (d, *J* = 8.0 Hz, 1H, Ar-H), 7.32 (dt, *J* = 1.0, 8.0 Hz, 1H, Ar-H), 7.23 (t, *J* = 7.5 Hz, 1H, Ar-H), 7.13 (d, *J* = 8.2 Hz, 2H, Ar-H), 7.06 (d, *J* = 8.2 Hz, 2H, Ar-H), 2.61 (q, *J* = 7.6 Hz, 2H, $CH_2$), 1.23 (t, *J* = 7.6 Hz, 3H, $CH_3$). $^{13}$C NMR (101 MHz, $CDCl_3$): *δ* 141.3,136.5, 135.9, 130.7, 129.2, 128.5, 126.3, 123.1, 120.9, 119.7, 111.8, 103.2, 28.4, 15.7. MS (ESI): 254 (M+H$^+$, 100). Anal calcd for $C_{16}H_{15}NS$: C, 75.85; H, 5.97; N, 5.53; S, 12.65. Found C, 75.59; H, 5.63; N, 5.71; S, 12.32.

3-((2,4-Dimethylphenyl)thio)-1*H*-indole (**6af**)

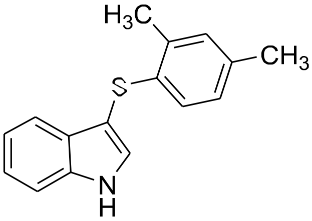

Tawny amorphous solid. $^{1}H$ NMR (400 MHz, $CDCl_3$): $\delta$ 8.28 (s, 1H, NH), 7.71 (d, $J$ = 8.0 Hz, 1H, Ar-H), 7.45 (d, $J$ = 8.0 Hz, 1H, Ar-H), 7.41 (d, $J$ = 2.6 Hz, 1H, Ar-H), 7.36 (t, $J$ = 7.7 Hz, 1H, Ar-H), 7.27 (t, $J$ = 7.7 Hz, 1H, Ar-H), 7.09 (s, 1H, Ar-H), 6.83 (d, $J$ = 8.0 Hz, 1H, Ar-H), 6.78 (d, $J$ = 8.0 Hz, 1H, Ar-H), 2.59 (s, 3H, $CH_3$), 2.34 (s, 3H, $CH_3$). $^{13}C$ NMR (101 MHz, $CDCl_3$): $\delta$ 136.6, 134.7, 134.6, 134.4, 130.9, 130.5, 129.3, 127.1, 126.1, 123.0, 120.8, 119.7, 111.6, 103.0, 20.7, 19.9. MS (ESI): 254 ($M+H^+$, 100). Anal calcd for $C_{16}H_{15}NS$: C, 75.85; H, 5.97; N, 5.53; S, 12.65. Found C, 76.04; H, 5.83; N, 5.66; S, 12.35.

3-((4-Nitrophenyl)thio)-1*H*-indole (**6ag**)

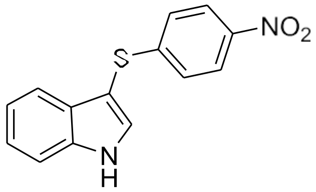


Reddish brown amorphous solid. $^{1}H$ NMR (400 MHz, $CDCl_3$): $\delta$ 8.86 (s, 1H, NH), 8.02 (d, $J$ = 9.0 Hz, 2H, Ar-H), 7.55 (t, $J$ = 7.7 Hz, 1H, Ar-H), 7.54 (d, $J$ = 2.6 Hz, 1H, Ar-H), 7.53 (d, $J$ = 8.1 Hz, 1H, Ar-H), 7.34 (t, $J$ = 8.1 Hz, 1H, Ar-H), 7.22 (t, $J$ = 7.7 Hz, 1H, Ar-H), 7.15 (d, $J$ = 9.0 Hz, 2H, Ar-H). $^{13}C$ NMR (101 MHz, $CDCl_3$): $\delta$ 150.0, 144.9, 136.7, 131.4, 128.5, 125.2, 123.9, 123.5, 121.4, 119.2, 112.1, 100.1. MS (ESI): 271 ($M+H^+$, 100). These assignments matched with those previously published.[5]

3-(Naphthalen-2-ylthio)-1*H*-indole (**6ah**)

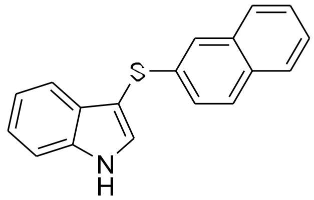


Reddish brown amorphous solid. $^{1}H$ NMR (400 MHz, DMSO-$d_6$): $\delta$ 11.74 (s, 1H, NH), 7.83 (d, $J$ = 2.5 Hz, 1H, Ar-H), 7.77 (d, $J$ = 8.6 Hz, 1H, Ar-H), 7.73 (d, $J$ = 8.7 Hz, 1H, Ar-H), 7.61 (d, $J$ = 7.5 Hz, 1H, Ar-H), 7.50 (d, $J$ = 2.6 Hz, 1H, Ar-H), 7.48 (s, 1H, Ar-H), 7.40 – 7.33 (m, 3H, Ar-H), 7.20 (dd, $J$ = 2.6 Hz, 1H, Ar-H), 7.17 (t, $J$ = 2.6 Hz, 1H, Ar-H), 7.03 (t, $J$ = 7.5 Hz, 1H, Ar-H). $^{13}C$ NMR (101 MHz, DMSO-$d_6$): $\delta$ 137.3, 137.2, 133.7, 133.0, 131.3, 129.1, 128.8, 128.1, 127.1, 127.0, 125.7, 124.9, 123.3, 122.6, 120.6, 118.8, 112.9, 99.8. MS (ESI): 276 ($M+H^+$, 100). These assignments matched with those previously published.[3]

5-Methoxy-3-((4-methoxyphenyl)thio)-1*H*-indole (**6ai**).

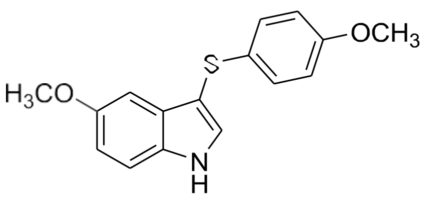


Red amorphous solid. $^{1}H$ NMR (400 MHz, $CDCl_3$): $\delta$ 8.42 (s, 1H), 7.40 (d, $J$ = 2.6 Hz, 1H), 7.28 (d, $J$ = 8.8 Hz, 1H), 7.17 (dt, $J$ = 2.6, 8.8 Hz, 2H), 7.13 (d, $J$ = 2.4 Hz, 1H), 6.95 (dd, $J$ = 8.8, 2.4 Hz, 1H), 6.80 (dt, $J$ = 2.6, 8.8 Hz, 2H), 3.84 (s, 3H), 3.76 (s, 3H). $^{13}C$ NMR (101 MHz, $CDCl_3$): $\delta$ 157.8, 155.0, 131.5, 131.0, 129.9, 129.8, 128.3, 114.6, 113.4, 112.5, 103.7, 104.0, 55.9, 55.4. These assignments matched with those previously published.[6]

3-(*p*-Tolylthio)-1*H*-pyrrolo[3,2-*b*]pyridine (**6aj**).

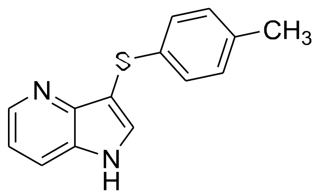

Light yellow amorphous solid. $^1$H NMR (400 MHz, DMSO-$d_6$): $\delta$ 11.86 (s, 1H, NH), 8.36 (d, $J$ = 4.0 Hz, 1H, Ar-H), 8.00 (d, $J$ = 2.6 Hz, 1H, Ar-H), 7.86 (d, $J$ = 8.1 Hz, 1H, Ar-H), 7.19 (dd, $J$ = 8.1, 4.5 Hz, 1H, Ar-H), 7.00 (d, $J$ = 7.2 Hz, 2H, Ar-H), 6.96 (d, $J$ = 7.2 Hz, 2H, Ar-H), 2.19 (s, 3H, $CH_3$). $^{13}$C NMR (101 MHz, DMSO-$d_6$): $\delta$ 146.0, 143.8, 136.2, 135.9, 134.5, 129.8, 129.7, 126.5, 120.0, 117.7, 101.6, 20.9. MS (ESI): 241 ($M+H^+$, 100). Anal calcd for $C_{14}H_{12}N_2S$: C, 69.97; H, 5.03; N, 11.66; S, 13.34. Found C, 70.21; H, 5.37; N, 11.31; S, 13.15.

2-((1*H*-indol-3-yl)thio)benzo[*d*]thiazole (**6ak**)

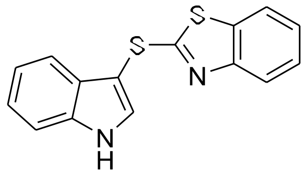

Brown amorphous solid. $^1$H NMR (400 MHz, DMSO-$d_6$): $\delta$ 12.03 (s, 1H, NH), 8.04 (d, $J$ = 2.8 Hz, 1H, Ar-H), 7.82 (dd, $J$ = 2.8, 1.8 Hz, 1H, Ar-H), 7.80 (dd, $J$ = 2.1, 1.0 Hz, 1H, Ar-H), 7.57 (d, $J$ = 7.8 Hz, 1H, Ar-H), 7.56 (d, $J$ = 7.8 Hz, 1H, Ar-H), 7.41 (dt, $J$ = 1.2, 8.4 Hz, 1H, Ar-H), 7.30-7.23 (m, 2H, Ar-H), 7.15 (dt, $J$ = 1.0, 7.1 Hz, 1H, Ar-H). $^{13}$C NMR (101 MHz, DMSO-$d_6$): $\delta$ 173.8, 154.6, 137.2, 135.4, 134.4, 128.4, 126.6, 124.4, 123.1, 122.1, 121.6, 121.3, 118.5, 113.1, 97.7. MS (ESI): 283 ($M+H^+$, 100). These assignments matched with those previously published.[7]

3-((1-Methyl-1*H*-imidazol-2-yl)thio)-1*H*-indole (**6al**)

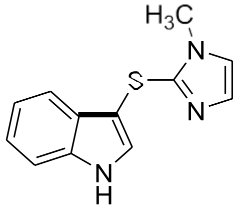

Yellow amorphous solid. $^1$H NMR (400 MHz, DMSO-$d_6$): $\delta$ 11.51 (s, 1H, NH), 7.69 (s, 1H, Ar-H), 7.60 (d, $J$ = 7.6 Hz, 1H, Ar-H), 7.39 (d, $J$ = 7.6 Hz, 1H, Ar-H), 7.16 (s, 1H, Ar-H), 7.10 (t, $J$ = 7.1 Hz, 1H, Ar-H), 7.04 (t, $J$ = 7.1 Hz, 1H, Ar-H), 6.85 (s, 1H, Ar-H), 3.66 (s, 3H, $CH_3$). $^{13}$C NMR (101 MHz, DMSO-$d_6$): $\delta$ 140.0, 136.7, 131.0, 128.9, 128.5, 124.0, 122.4, 120.3, 119.1, 112.5, 100.6, 34.0. MS (ESI): 230 ($M+H^+$, 100). Anal calcd for $C_{12}H_{11}N_3S$: C, 62.86; H, 4.84; N, 18.33; S, 13.98. Found C, 63.10; H, 5.07; N, 18.05; S, 13.61.

2-((1*H*-indol-3-yl)thio)-1,3,4-thiadiazole (**6am**)

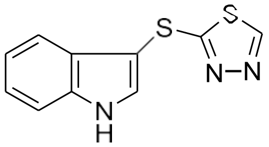

Yellow amorphous solid. $^1$H NMR (400 MHz, DMSO-$d_6$): $\delta$ 11.98 (s, 1H, NH), 9.29 (s, 1H, Ar-H), 8.00 (d, $J$ = 2.8 Hz, 1H, Ar-H), 7.56 (d, $J$ = 8.0 Hz, 1H, Ar-H), 7.53 (d, $J$ = 7.9 Hz, 1H, Ar-H), 7.25 (dt, $J$ = 1.0, 8.0 Hz, 1H, Ar-H), 7.16 (d, $J$ = 7.9 Hz, 1H, Ar-H). $^{13}$C NMR (101 MHz,

DMSO-$d_6$): $\delta$ 173.0, 154.3, 137.2, 133.6, 127.8, 123.2, 121.3, 118.4, 113.2, 98.6. MS (ESI): 234 ($M+H^+$, 100). Anal calcd for $C_{10}H_7N_3S_2$: C, 51.48; H, 3.02; N, 18.01; S, 27.48. Found C, 51.83; H, 3.39; N, 17.85; S, 27.17.

2-((1*H*-indol-3-yl)thio)-5-methyl-1,3,4-thiadiazole (**6an**)

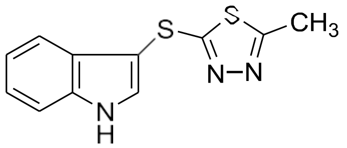

Yellow amorphous solid. $^1H$ NMR (400 MHz, DMSO-$d_6$): $\delta$ 11.95 (s, 1H, NH), 7.96 (d, $J$ = 2.7 Hz, 1H, Ar-H), 7.55 (d, $J$ = 2.7 Hz, 1H, Ar-H), 7.53 (d, $J$ = 2.7 Hz, 1H, Ar-H), 7.24 (d, $J$ = 7.5 Hz, 1H, Ar-H), 7.16 (d, $J$ = 7.5 Hz, 1H, Ar-H), 2.50 (s, 3H, $CH_3$). $^{13}C$ NMR (101 MHz, $CDCl_3$): $\delta$ 172.4, 165.7, 137.1, 133.5, 128.0, 123.1, 121.3, 118.4, 113.1, 98.8, 15.6. MS (ESI): 248 ($M+H^+$, 100). Anal calcd for $C_{11}H_9N_3S_2$: C, 53.42; H, 3.67; N, 16.99; S, 25.92. Found C, 53.76; H, 3.92; N, 16.84; S, 25.59.

3-((1-Methyl-1*H*-tetrazol-5-yl)thio)-1*H*-indole (**6ao**)

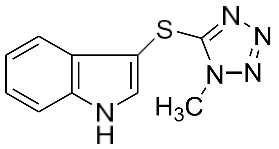

Pink amorphous solid. $^1H$ NMR (400 MHz, DMSO-$d_6$): $\delta$ 11.85 (s, 1H, NH), 7.94 (d, $J$ = 2.7 Hz, 1H, Ar-H), 7.54 (d, $J$ = 8.7 Hz, 1H, Ar-H), 7.51 (d, $J$ = 8.7 Hz, 1H, Ar-H), 7.22 (d, $J$ = 7.2 Hz, 1H, Ar-H), 7.13 (d, $J$ = 7.2 Hz, 1H, Ar-H), 4.03 (s, 3H, $NCH_3$). $^{13}C$ NMR (101 MHz, DMSO-$d_6$): $\delta$ 153.8, 136.9, 133.5, 128.8, 122.9, 121.0, 118.6, 112.9, 94.5, 34.5. MS (ESI): 232 ($M+H^+$, 100). Anal calcd for $C_{10}H_9N_5S$: C, 51.93; H, 3.92; N, 30.28; S, 13.86. Found C, 52.20; H, 4.28; N, 29.91; S, 13.93.

3-Thiocyanato-1*H*-indole (**7a**)

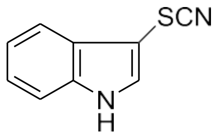

White amorphous solid. $^1H$ NMR (400 MHz, $CDCl_3$): $\delta$ 8.76 (s, 1H, NH), 7.83 (dd, $J$ = 5.9, 3.1 Hz, 1H, Ar-H), 7.52 (d, $J$ = 2.8 Hz, 1H, Ar-H), 7.45 (dt, $J$ = 5.9, 3.1 Hz, 1H, Ar-H), 7.35 (t, $J$ = 3.1 Hz, 1H, Ar-H), 7.33 (t, $J$ = 3.1 Hz, 1H, Ar-H). $^{13}C$ NMR (101 MHz, $CDCl_3$): $\delta$ 136.0, 131.0, 127.7, 123.9, 121.9, 118.8, 112.1, 111.9, 92.3. MS (ESI): 175 ($M+H^+$, 100). These assignments matched with those previously published.[8]

5-Chloro-3-thiocyanato-1-*H*-indole (**7b**).

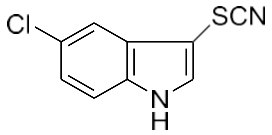

Yellow amorphous solid. $^1H$ NMR (400 MHz, DMSO-$d_6$): $\delta$ 12.20 (s, 1H, NH), 8.05 (s, 1H, Ar-H), 7.66 (s, 1H, Ar-H), 7.55 (d, $J$ = 7.7 Hz, 1H, Ar-H), 7.26 (d, $J$ = 7.7 Hz, 1H, Ar-H). $^{13}C$ NMR (101 MHz, DMSO-$d_6$): $\delta$ 135.3, 135.2, 129.1, 126.4, 123.5, 117.4, 115.0, 112.5, 89.9. MS (ESI): 209 ($M+H^+$, 100), 211 ($M+H^+$, 32). These assignments matched with those previously published.[9]

5-Methyl-3-thiocyanato-1-*H*-indole (**7c**).

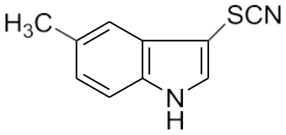


White amorphous solid. $^1$H NMR (400 MHz, DMSO-$d_6$): $\delta$ 12.00 (s, 1H, NH), 7.94 (d, $J$ = 2.9 Hz, 1H, Ar-H), 7.35 (d, $J$ = 8.2 Hz, 1H, Ar-H), 7.12 (t, $J$ = 8.2 Hz, 1H, Ar-H), 6.93 (d, $J$ = 7.1 Hz, 1H, Ar-H), 2.85 (s, 3H, $CH_3$). $^{13}$C NMR (101 MHz, DMSO-$d_6$): $\delta$ 137.2, 134.6, 129.9, 125.7, 123.4, 123.0, 114.3, 111.3, 89.8, 19.2. MS (ESI): 189 (M+H$^+$, 100). These assignments matched with those previously published.[10]

5-Methoxy-3-thiocyanato-1*H*-indole (**7d**)

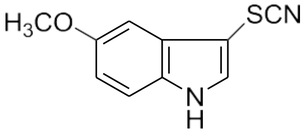


White amorphous solid. $^1$H NMR (400 MHz, $CDCl_3$): $\delta$ 8.55 (s, 1H, NH), 7.67 (d, $J$ = 8.7 Hz, 1H, Ar-H), 7.40 (d, $J$ = 2.7 Hz, 1H, Ar-H), 6.97 (dd, $J$ = 8.7, 2.1 Hz, 1H, Ar-H), 6.90 (d, $J$ = 2.1 Hz, 1H, Ar-H), 3.86 (s, 3H, $OCH_3$). $^{13}$C NMR (101 MHz, $CDCl_3$): $\delta$ 157.7, 136.9, 129.8, 121.8, 119.5, 112.1, 111.9, 95.2, 92.3, 55.7. MS (ESI): 205 (M+H$^+$, 100). These assignments matched with those previously published.[8]

3-Thiocyanato-1-*H*-indole-5-carbonitrile (**7e**).

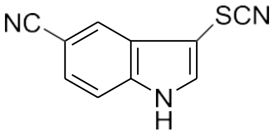


Yellow amorphous solid. $^1$H NMR (400 MHz, DMSO-$d_6$): $\delta$ 12.52 (s, 1H, NH), 8.23 (d, $J$ = 2.6 Hz, 1H, Ar-H), 8.21 (s, 1H, Ar-H), 7.71 (d, $J$ = 8.5 Hz, 1H, Ar-H), 7.64 (dd, $J$ = 8.6, 2.6 Hz, 1H, Ar-H). $^{13}$C NMR (101 MHz, DMSO-$d_6$): $\delta$ 143.4, 141.1, 132.5, 130.9, 128.6, 125.1, 119.5, 117.2, 108.7, 96.8. MS (ESI): 200 (M+H$^+$, 100). These assignments matched with those previously published.[9]

5-Nitro-3-thiocyanato-1-*H*-indole (**7f**).

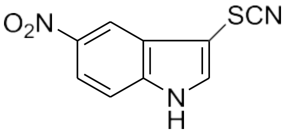


Yellow amorphous solid. $^1$H NMR (400 MHz, DMSO-$d_6$): $\delta$ 12.63 (s, 1H, NH), 8.51 (d, $J$ = 1.8 Hz, 1H, Ar-H), 8.27 (s, 1H, Ar-H), 8.12 (dd, $J$ = 9.0, 1.8 Hz, 1H, Ar-H), 7.70 (d, $J$ = 9.0 Hz, 1H, Ar-H). $^{13}$C NMR (101 MHz, DMSO-$d_6$): $\delta$ 142.6, 140.0, 137.5, 127.3, 118.6, 114.8, 114.1, 112.4, 93.6. MS (ESI): 220 (M+H$^+$, 100). These assignments matched with those previously published.[9]

4-Methyl-3-thiocyanato-1-*H*-indole (**7g**).

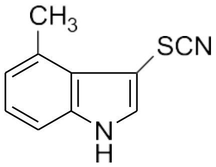

White amorphous solid. $^1$H NMR (400 MHz, DMSO-$d_6$): $\delta$ 11.91 (s, 1H, NH), 7.92 (d, $J$ = 2.9 Hz, 1H, Ar-H), 7.46 (s, 1H, Ar-H), 7.43 (d, $J$ = 8.3 Hz, 1H, Ar-H), 7.10 (dd, $J$ = 8.3, 1.1 Hz, 1H, Ar-H), 2.45 (s, 3H, $CH_3$). $^{13}$C NMR (101 MHz, DMSO-$d_6$): $\delta$ 135.1, 133.5, 130.5, 128.2, 125.0, 117.7, 113.0, 112.8, 89.0, 21.6. MS (ESI): 189 (M+H$^+$, 100). These assignments matched with those previously published.[9]

3-Thiocyanato-1*H*-pyrrolo[2,3-*b*]pyridine (**7h**)

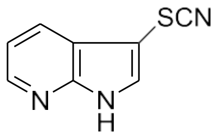


White amorphous solid. $^1$H NMR (400 MHz, DMSO): $\delta$ 12.62 (s, 1H, NH), 8.40 (d, $J$ = 4.5 Hz, 1H, Ar-H), 8.18 (s, 1H, Ar-H), 8.13 (d, $J$ = 7.8 Hz, 1H, Ar-H), 7.31 (dd, $J$ = 7.8, 4.7 Hz, 1H, Ar-H). $^{13}$C NMR (101 MHz, DMSO): $\delta$ 148.8, 145.0, 134.4, 127.0, 120.3, 117.8, 112.6, 89.5. MS (ESI): 176 (M+H$^+$, 100). These assignments matched with those previously published.[11]

3-(Phenylselanyl)-1*H*-indole (**9a**)

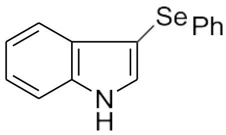


Yellow amorphous solid. $^1$H NMR (400 MHz, $CDCl_3$): $\delta$ 8.43 (s, 1H, NH), 7.69 (d, $J$ = 7.9 Hz, 1H, Ar-H), 7.49 (d, $J$ = 2.5 Hz, 1H, Ar-H), 7.46 (d, $J$ = 8.2 Hz, 1H, Ar-H), 7.33-7.27 (m, 3H, Ar-H), 7.24-7.12 (m, 4H, Ar-H). $^{13}$C NMR (101 MHz, $CDCl_3$): $\delta$ 136.4, 133.9, 131.3, 130.0, 129.0, 128.7, 125.6, 123.0, 120.9, 120.4, 111.4, 98.2. MS (ESI): 274 (M+H$^+$, 100). These assignments matched with those previously published.[12]

2-Methyl-3-(phenylselanyl)-1*H*-indole (**9b**)

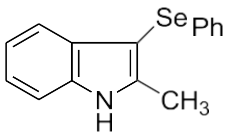


Yellow amorphous solid. $^1$H NMR (400 MHz, $CDCl_3$): $\delta$ 8.20 (s, 1H, NH), 7.64 (d, $J$ = 7.7 Hz, 1H, Ar-H), 7.36 (d, $J$ = 7.9 Hz, 1H, Ar-H), 7.26 (dd, $J$ = 3.5, 1.4 Hz, 1H, Ar-H), 7.23 (dd, $J$ = 3.5, 1.4 Hz, 2H, Ar-H), 7.20 (d, $J$ = 4.1 Hz, 1H, Ar-H), 7.19-7.12 (m, 3H, Ar-H), 2.56 (s, 3H, $CH_3$). $^{13}$C NMR (101 MHz, $CDCl_3$): $\delta$ 141.0, 135.8, 134.0, 131.3, 129.0, 128.4, 125.5, 122.2, 120.7, 119.8, 110.6, 96.2, 13.2. MS (ESI): 288 (M+H$^+$, 100). These assignments matched with those previously published.[13]

5-Methyl-3-(phenylselanyl)-1*H*-indole (**9c**)

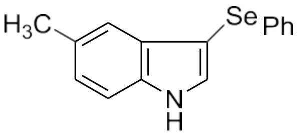


Yellow amorphous solid. $^1$H NMR (400 MHz, $CDCl_3$): $\delta$ 8.30 (s, 1H, NH), 7.51 (d, $J$ = 0.5 Hz, 1H, Ar-H), 7.44 (d, $J$ = 2.5 Hz, 1H, Ar-H), 7.35 (d, $J$ = 8.3 Hz, 1H, Ar-H), 7.31-7.28 (m, 2H, Ar-H), 7.22-7.14 (m, 4H, Ar-H), 2.49 (s, 3H, $CH_3$). $^{13}$C NMR (101 MHz, $CDCl_3$): $\delta$ 134.7, 134.1, 131.5,

130.4, 130.3, 129.0, 128.6, 125.6, 124.7, 119.9, 111.1, 97.4, 21.5. MS (ESI): 288 ($M+H^+$, 100). These assignments matched with those previously published.[14]

5-Methoxy-3-(phenylselanyl)-1*H*-indole (**9d**)

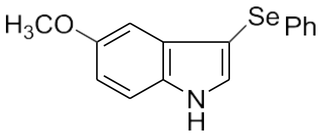


Yellow amorphous solid. $^1$H NMR (400 MHz, $CDCl_3$): $\delta$ 8.42 (s, 1H, NH), 7.44 (d, $J$ = 2.5 Hz, 1H, Ar-H), 7.33 (d, $J$ = 8.8 Hz, 1H, Ar-H), 7.30 (dd, $J$ = 8.2, 1.5 Hz, 1H, Ar-H), 7.29 (s, 1H, Ar-H), 7.21-7.13 (m, 4H, Ar-H), 6.97 (dd, $J$ = 8.8, 2.5 Hz, 1H, Ar-H), 3.85 (s, 3H, $OCH_3$). $^{13}$C NMR (101 MHz, $CDCl_3$): $\delta$ 155.1, 134.0, 132.0, 131.4, 130.8, 129.1, 128.5, 125.6, 113.5, 112.4, 101.6, 97.5, 55.9. MS (ESI): 304 ($M+H^+$, 100). These assignments matched with those previously published.[13]

## 4. Copies of $^{1}H$ and $^{13}C$ Spectra

$^{1}H$ and $^{13}C$ NMR Spectra for **6aa**

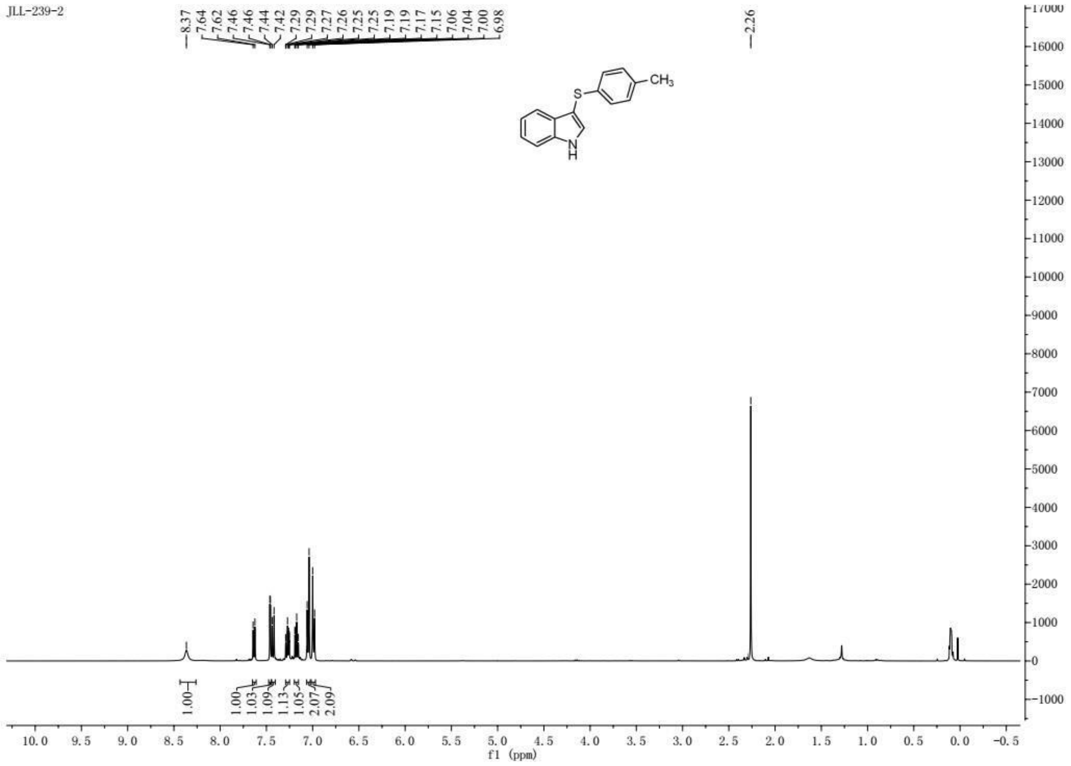


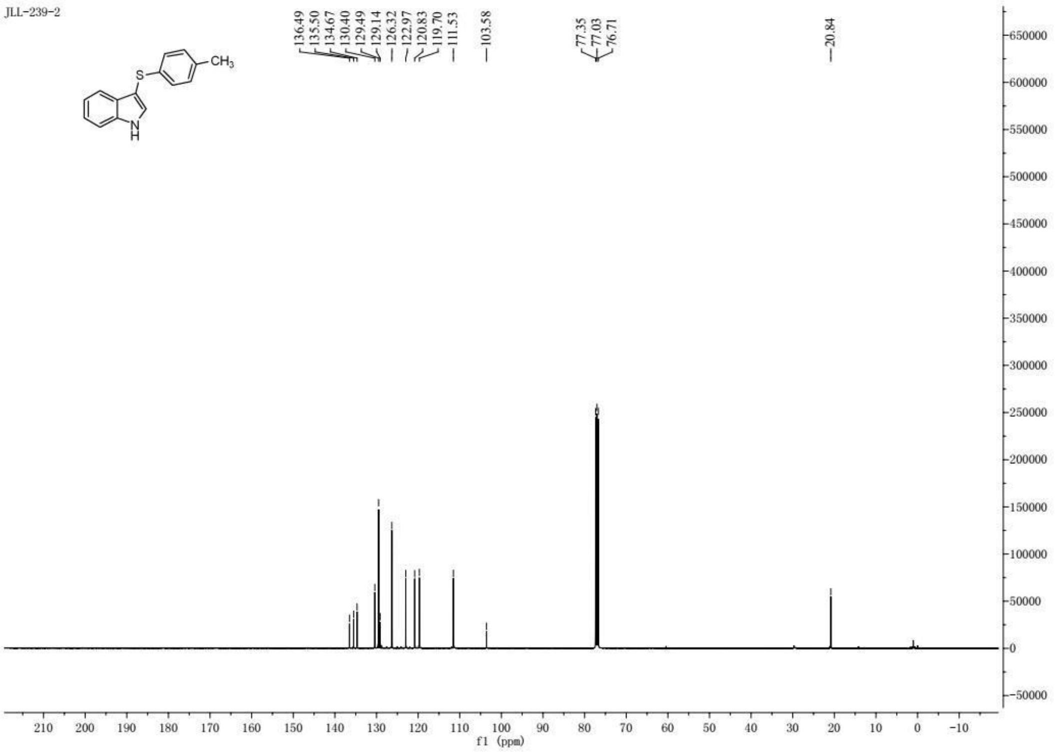

$^{1}H$ and $^{13}C$ NMR Spectra for **6ba**

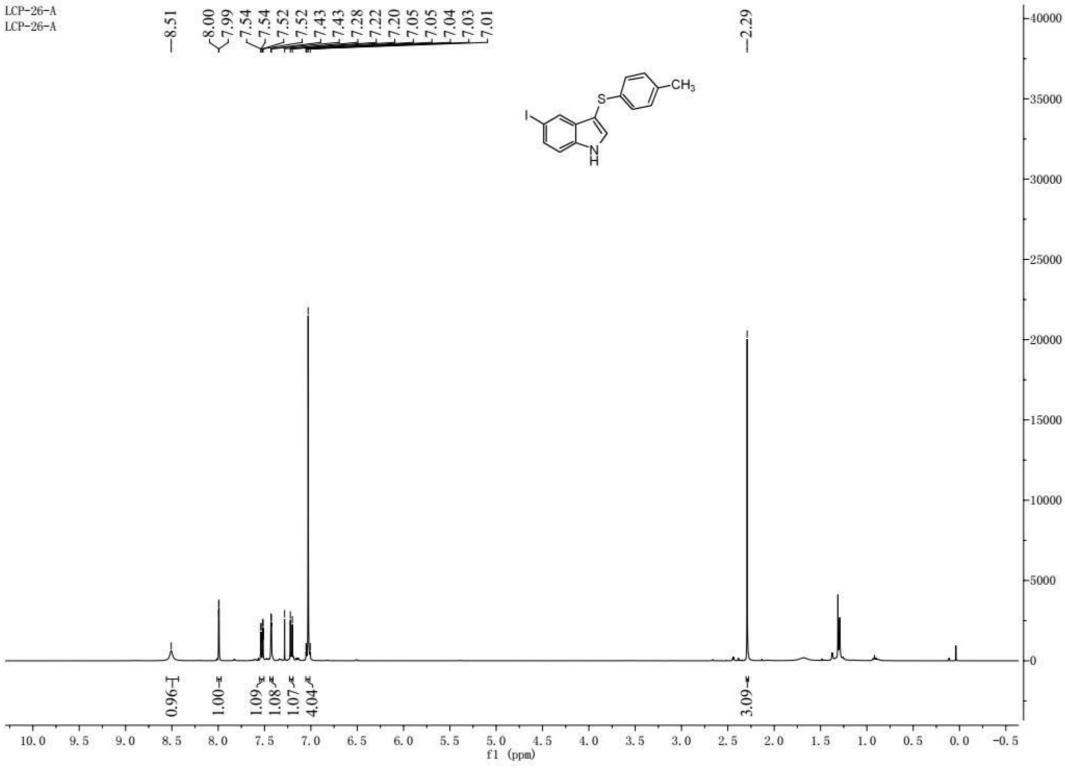


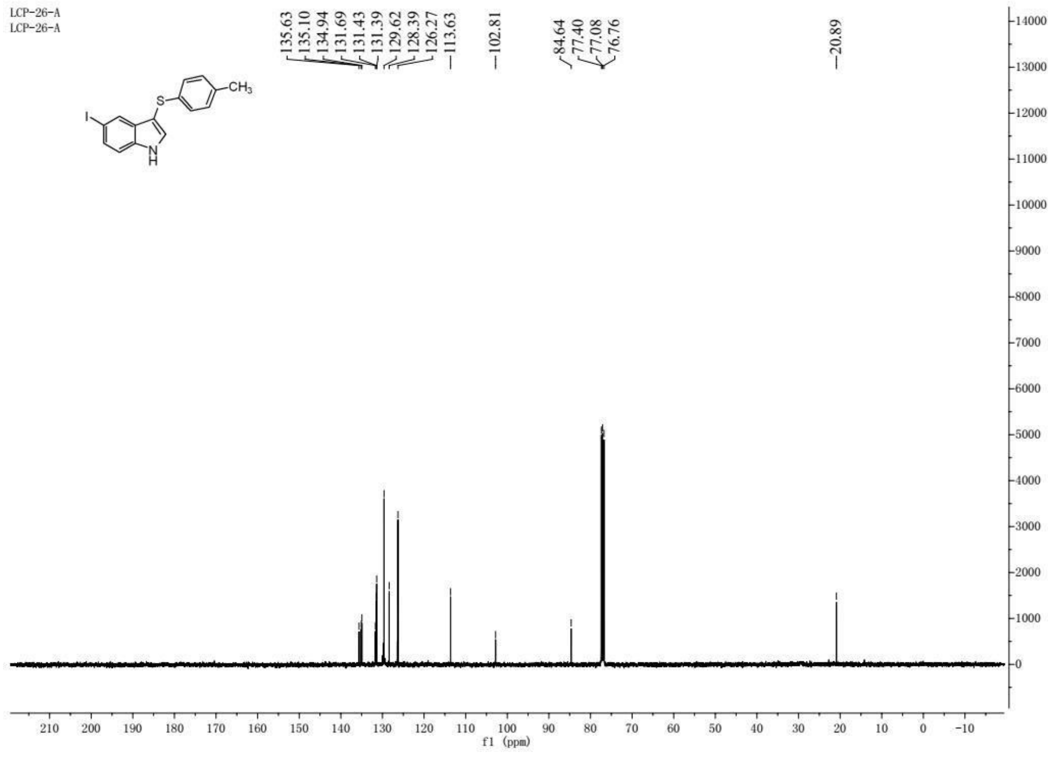

$^{1}$H and $^{13}$C NMR Spectra for **6ca**

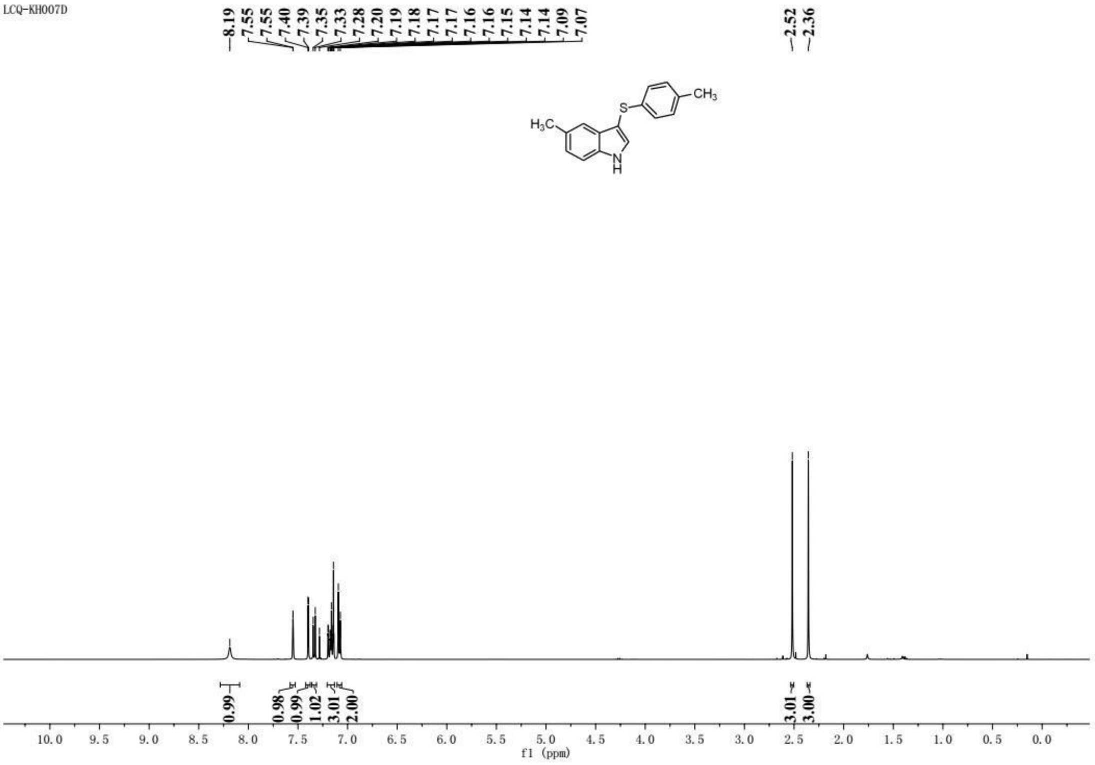


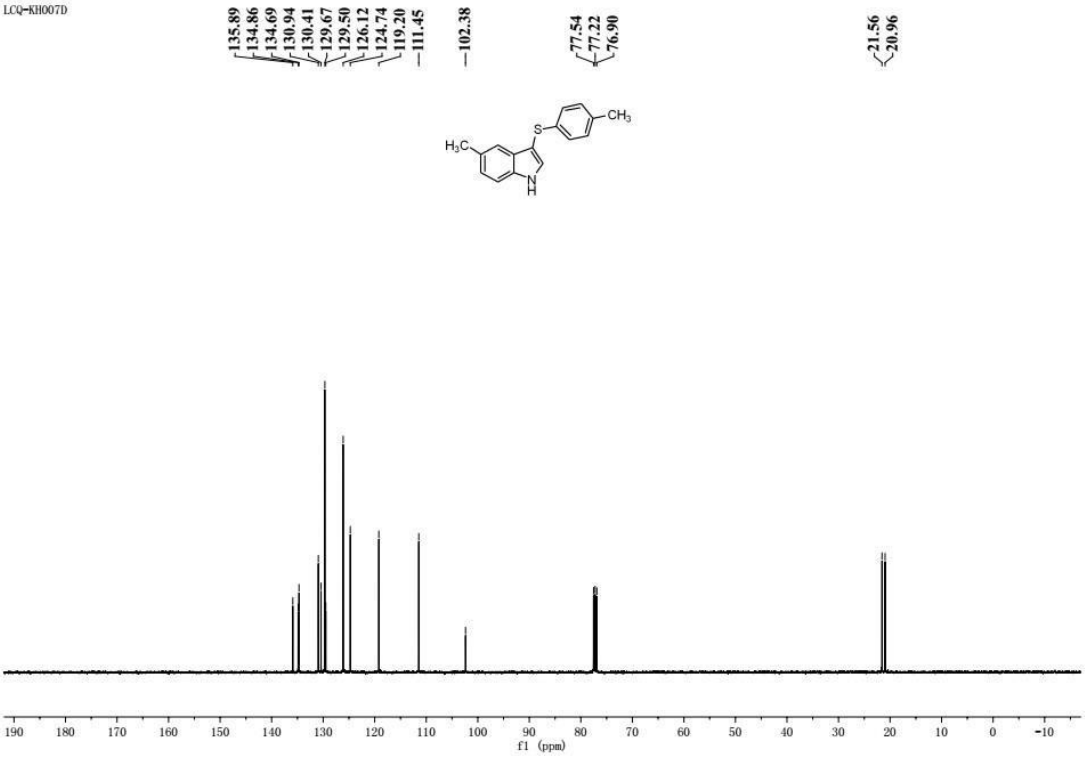

$^{1}$H and $^{13}$C NMR Spectra for **6da**

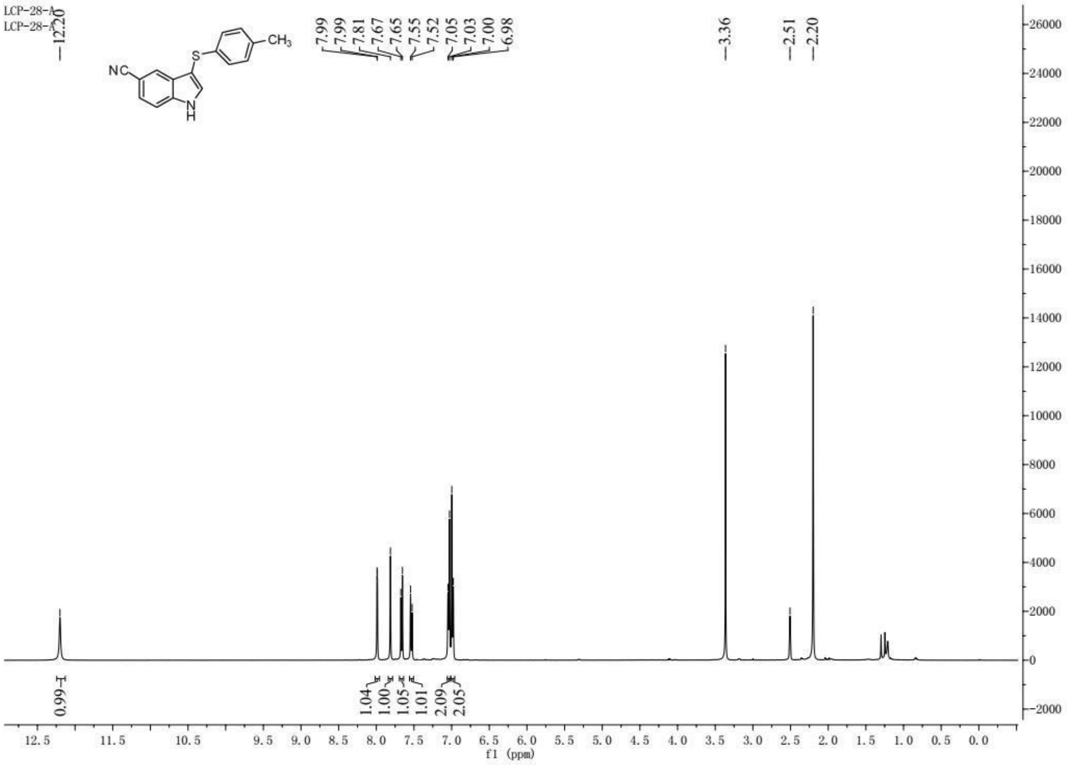


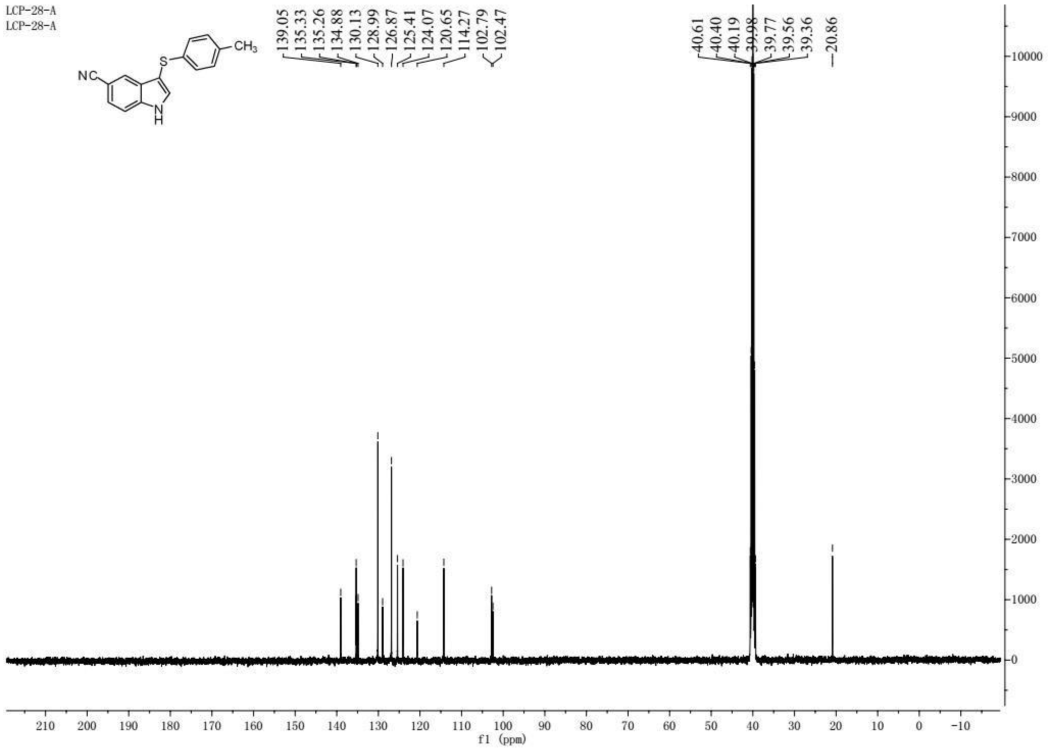

$^{1}H$ and $^{13}C$ NMR Spectra for **6ea**

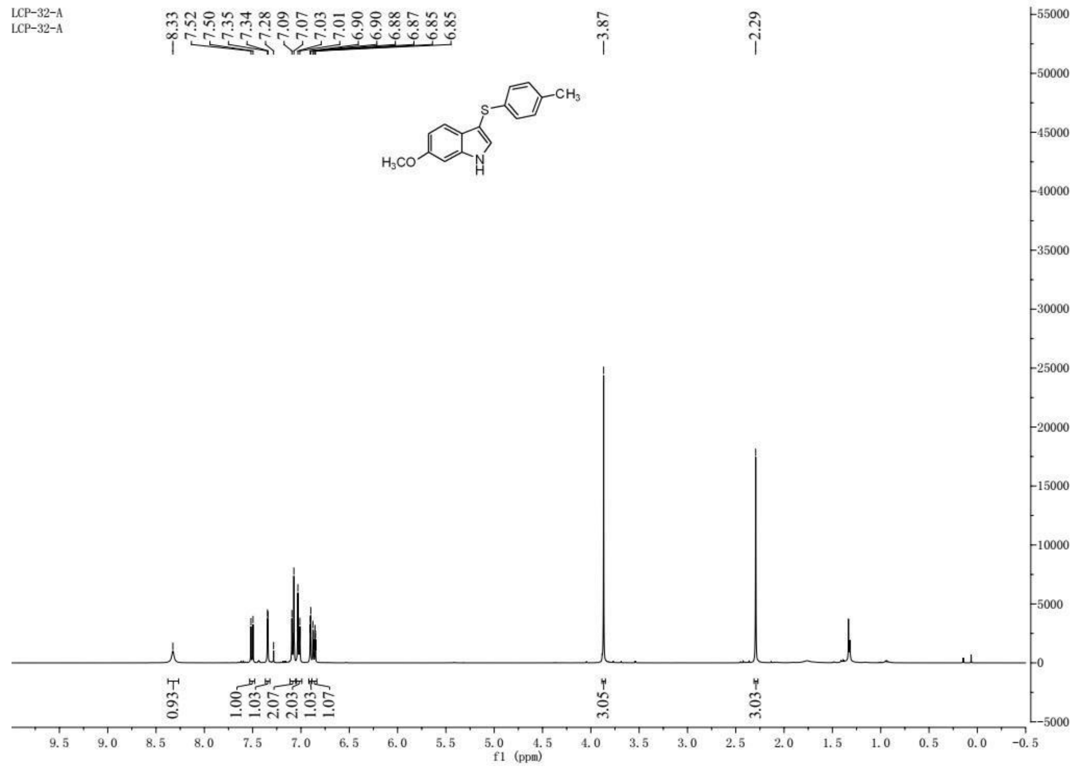

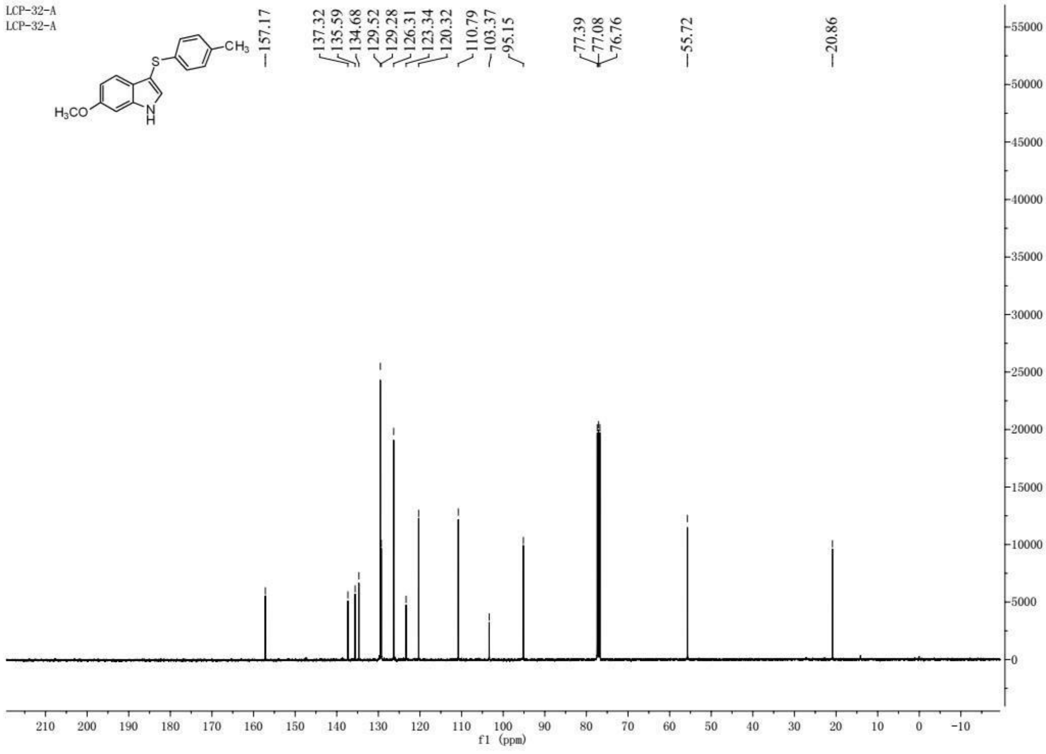

$^{1}$H and $^{13}$C NMR Spectra for **6fa**

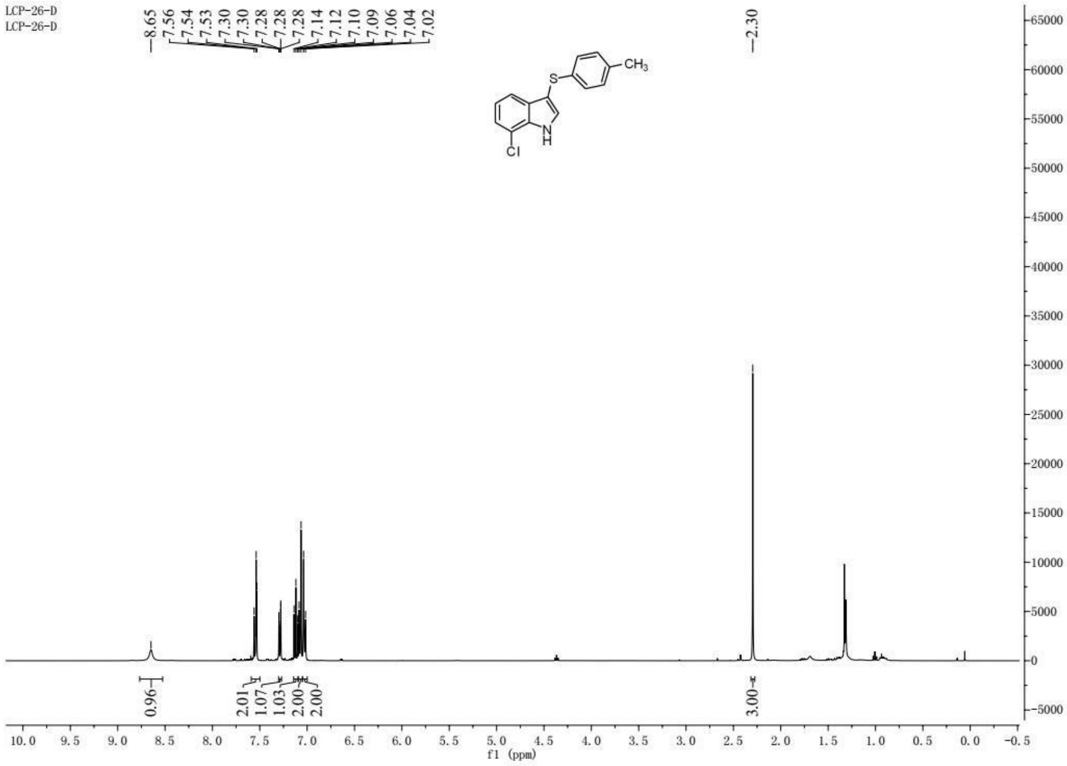


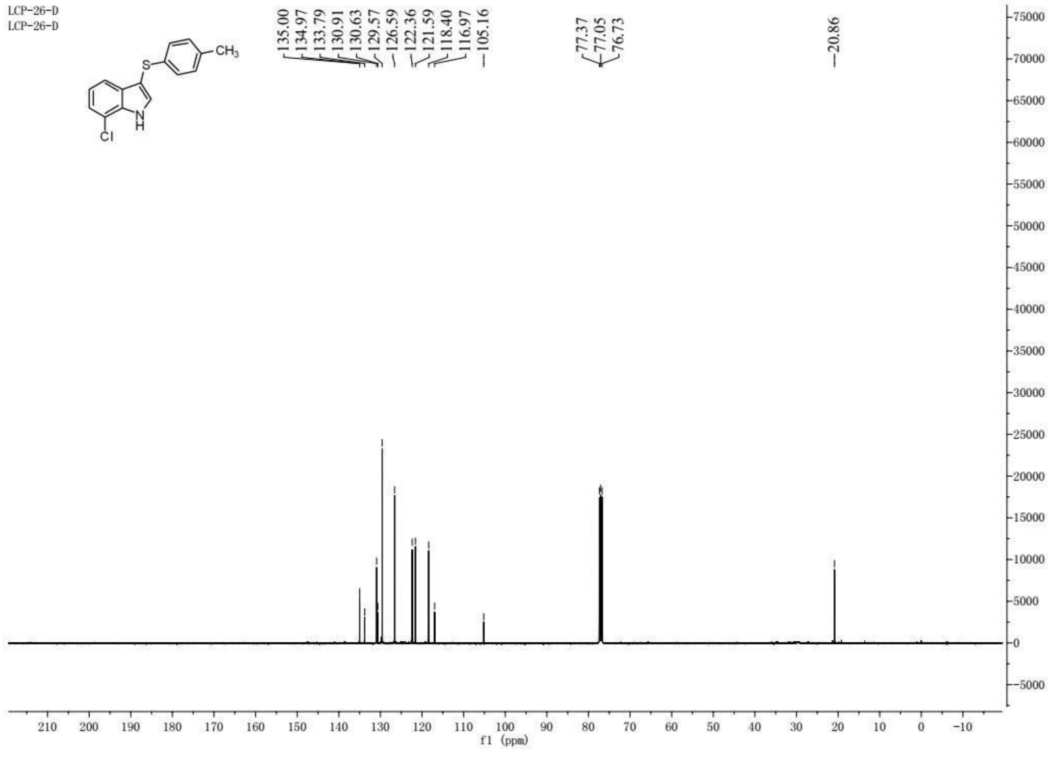

$^{1}H$ and $^{13}C$ NMR Spectra for **6ga**

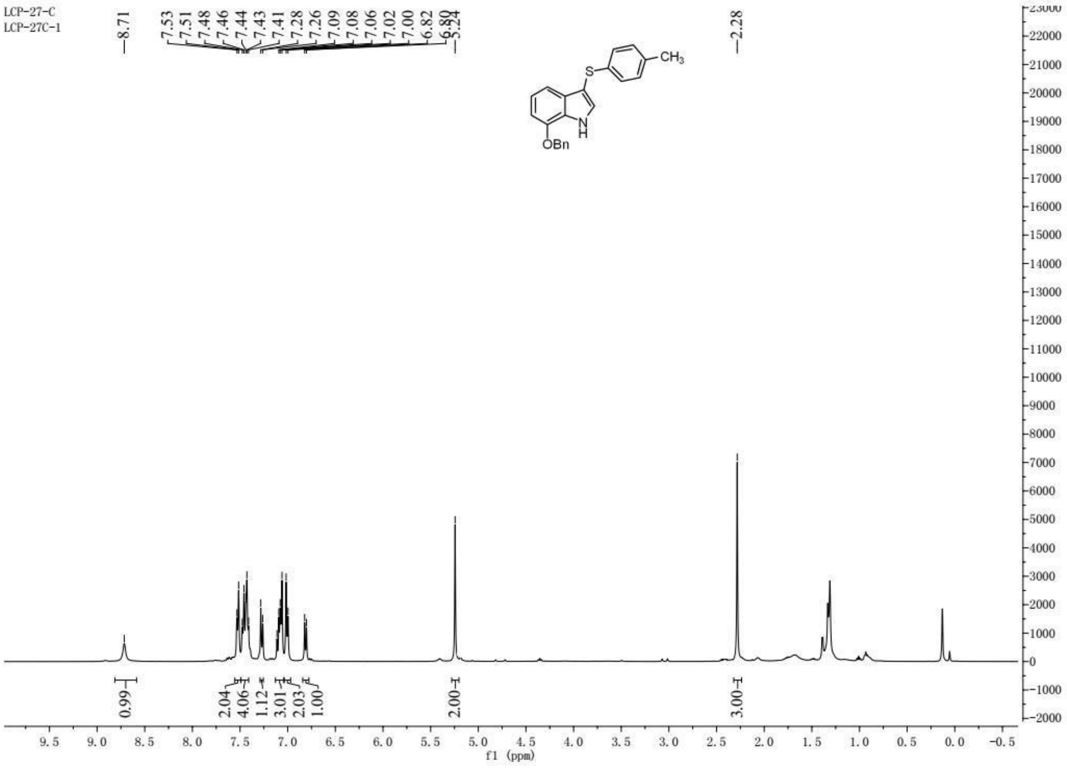


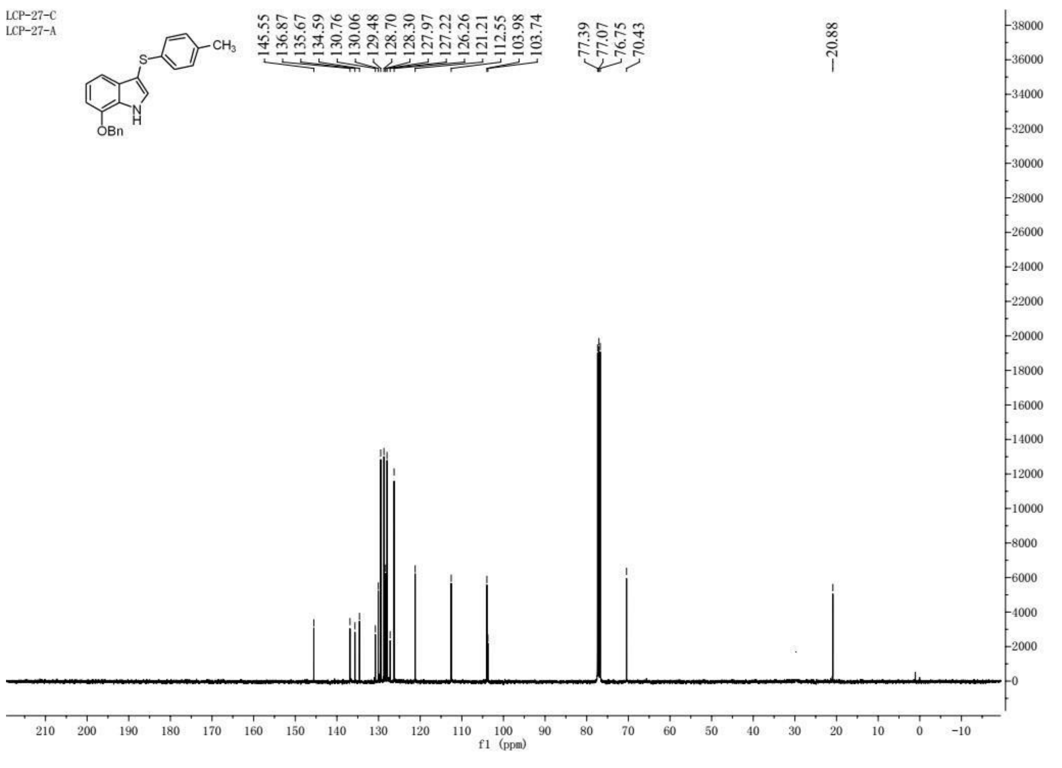

$^{1}H$ and $^{13}C$ NMR Spectra for **6ha**

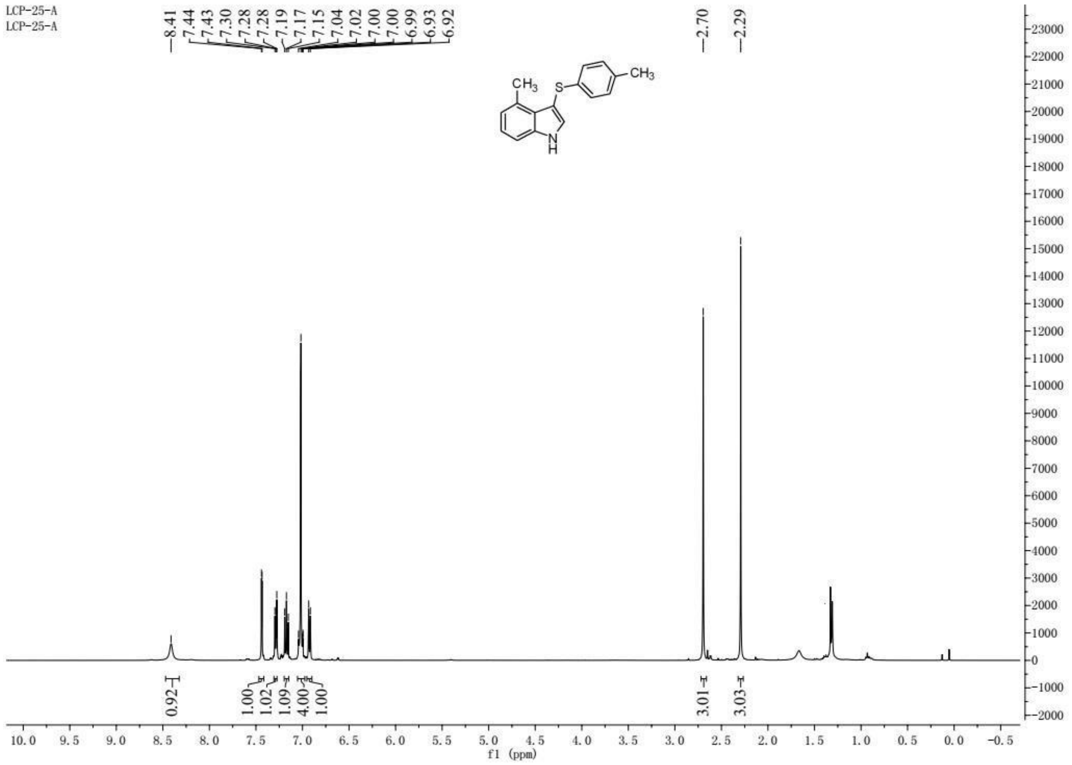

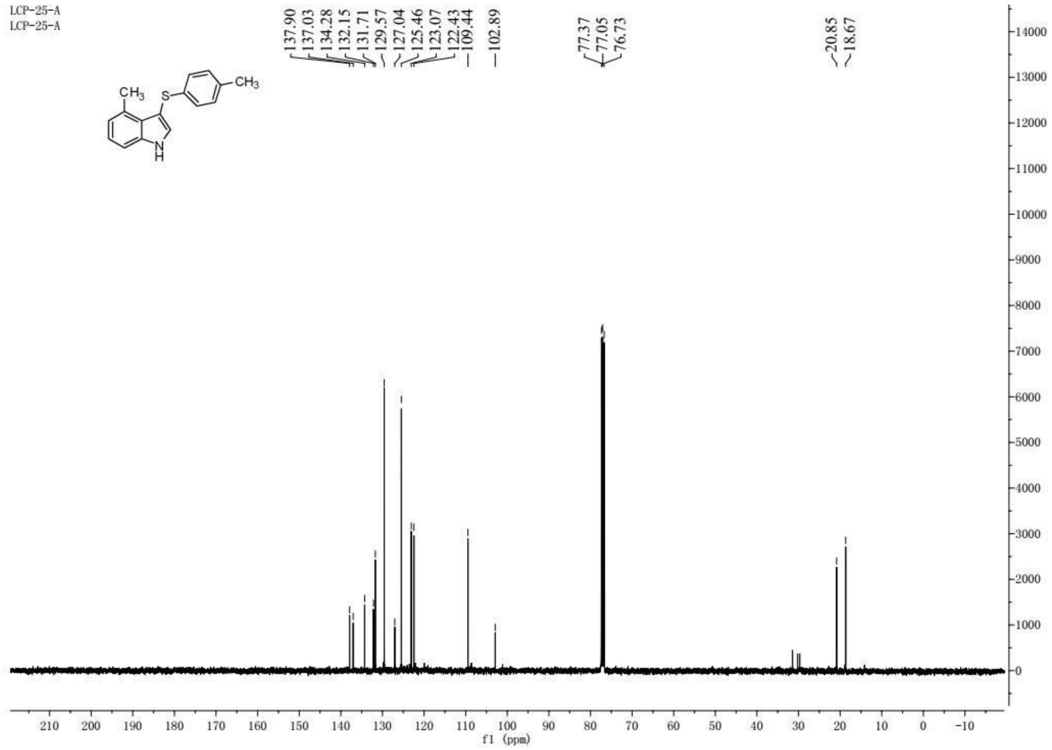

$^{1}$H and $^{13}$C NMR Spectra for **6ia**

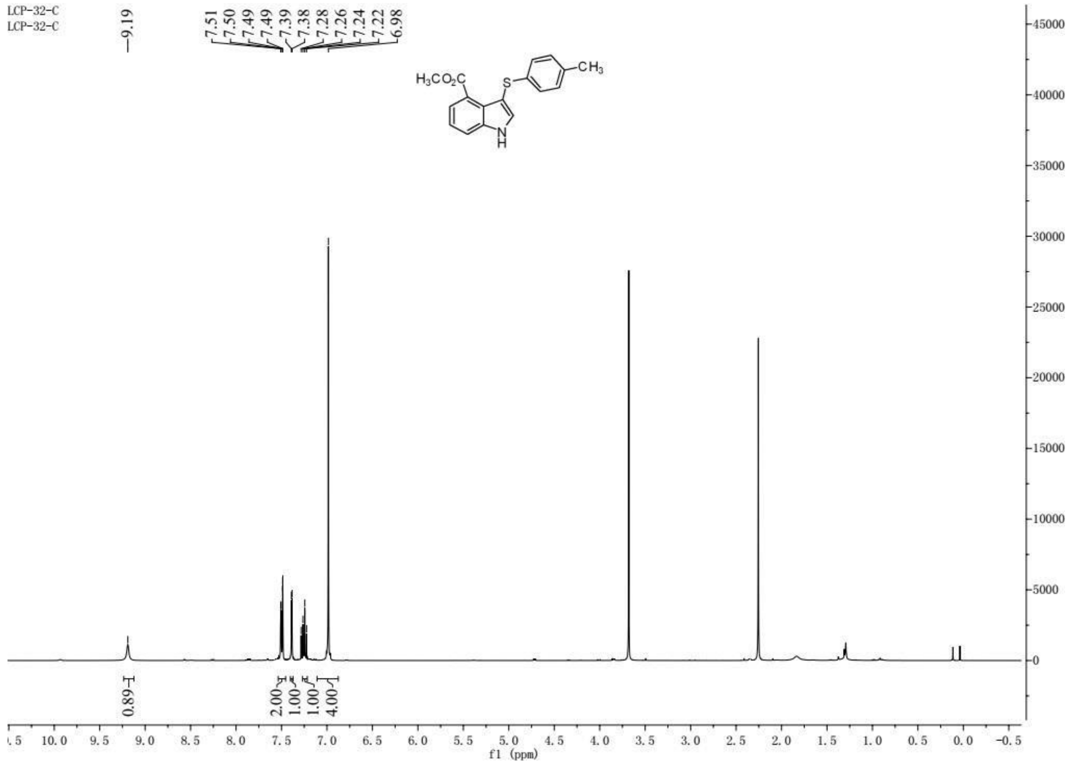


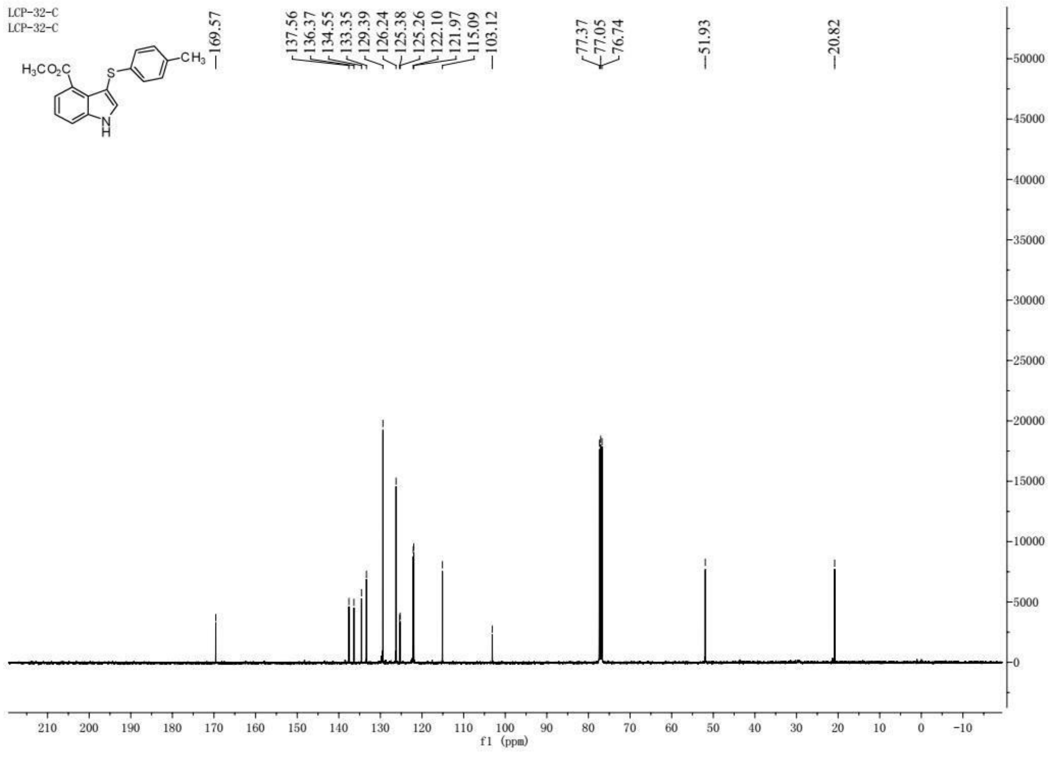

$^{1}$H and $^{13}$C NMR Spectra for **6ja**

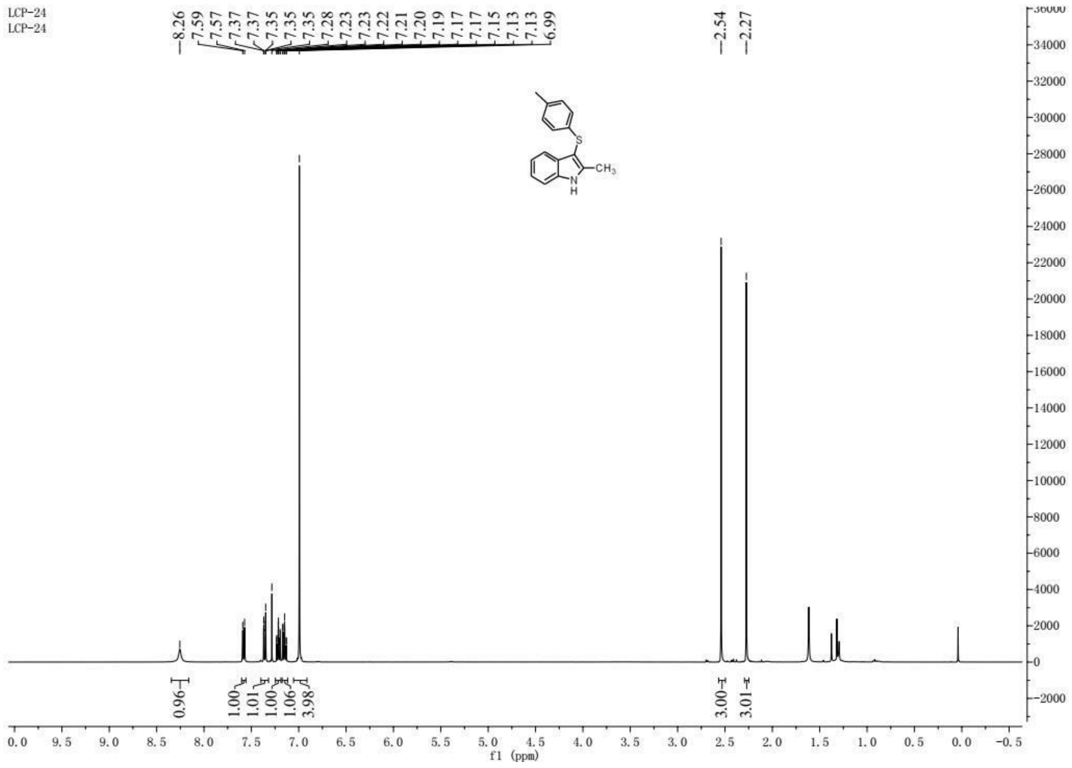


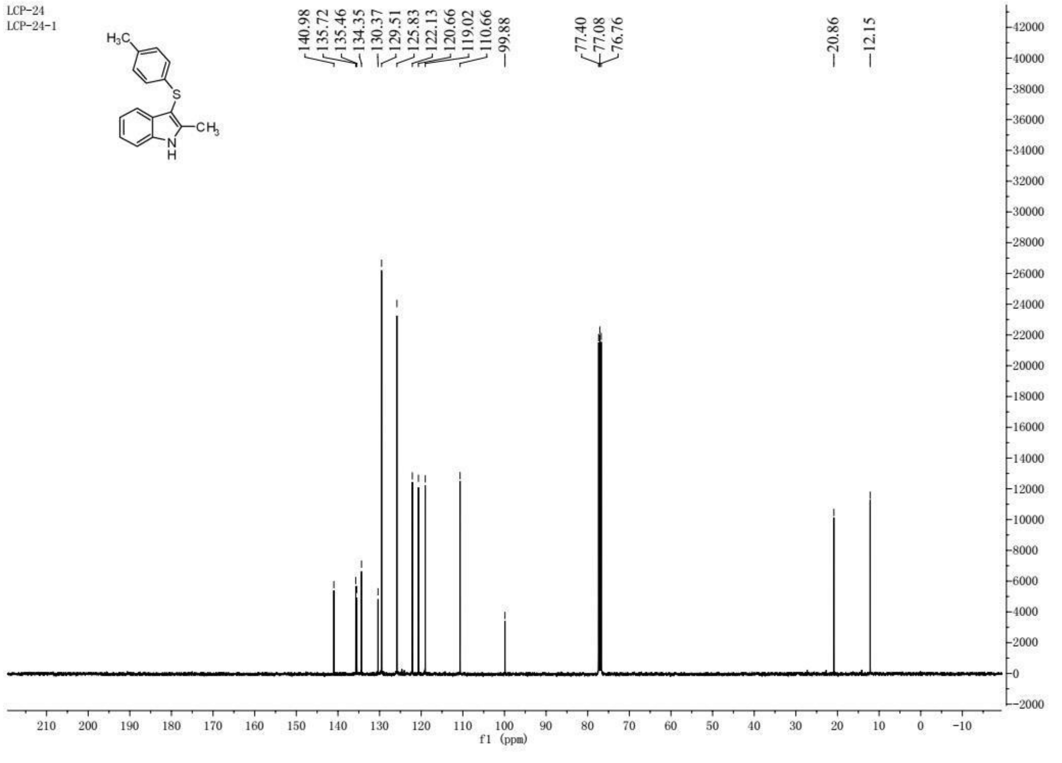

$^{1}H$ and $^{13}C$ NMR Spectra for **6ka**

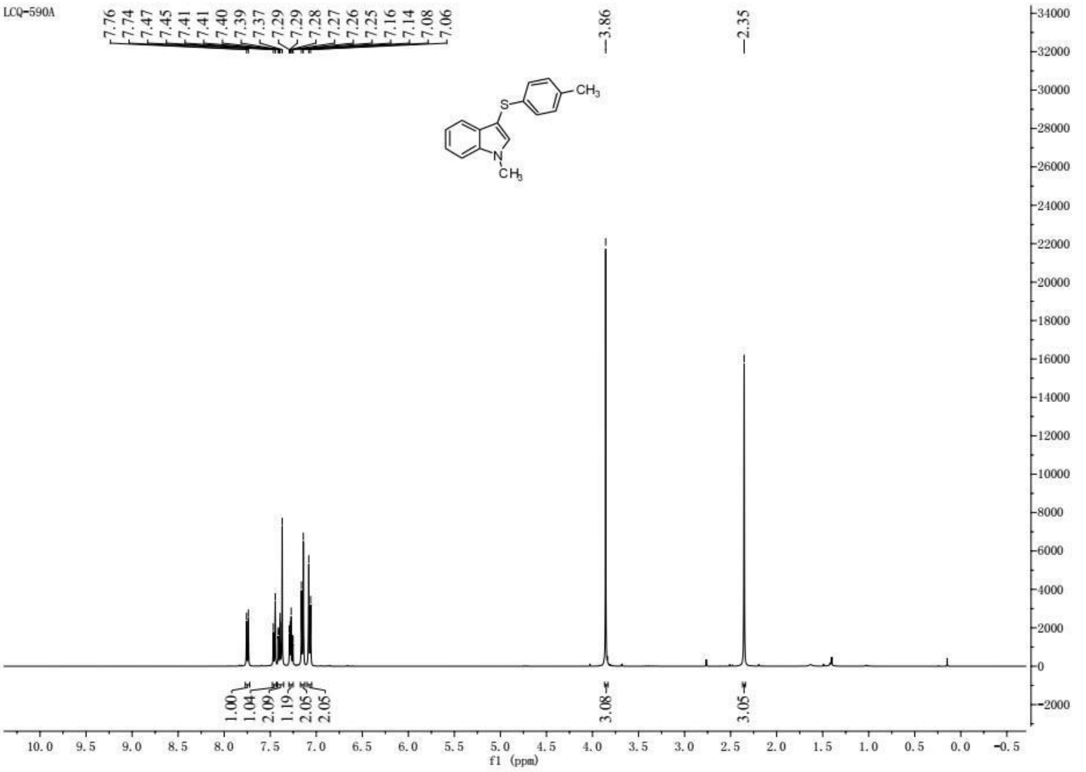


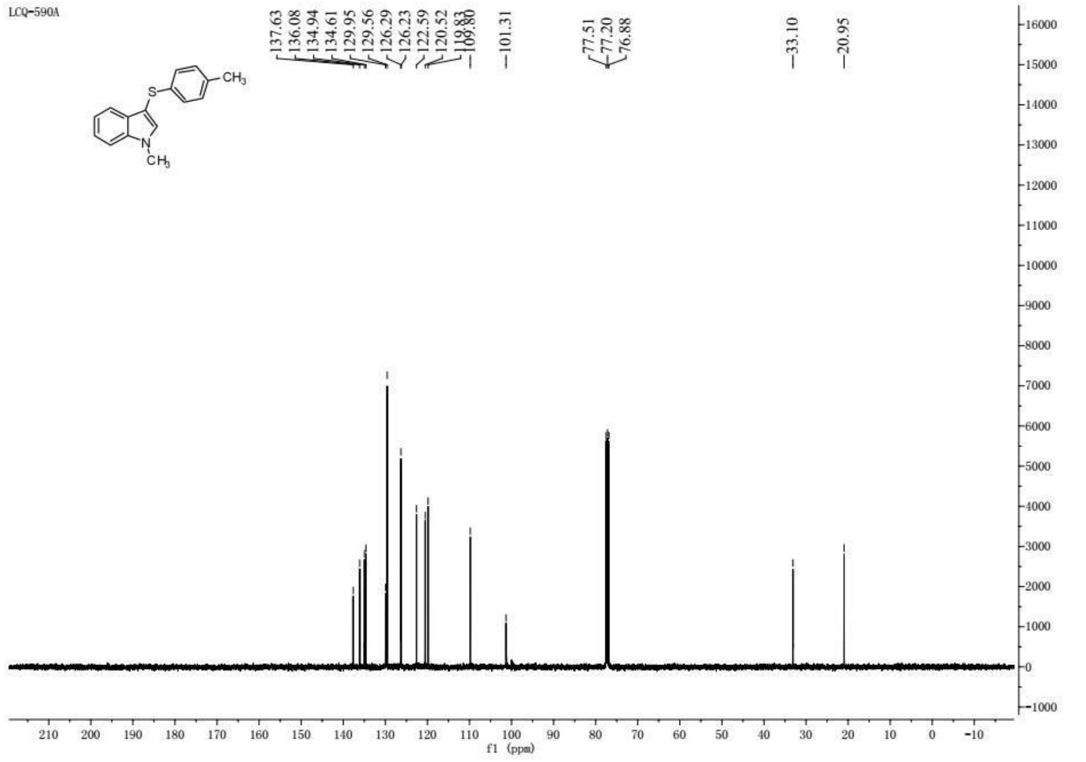

$^{1}H$ and $^{13}C$ NMR Spectra for **6la**

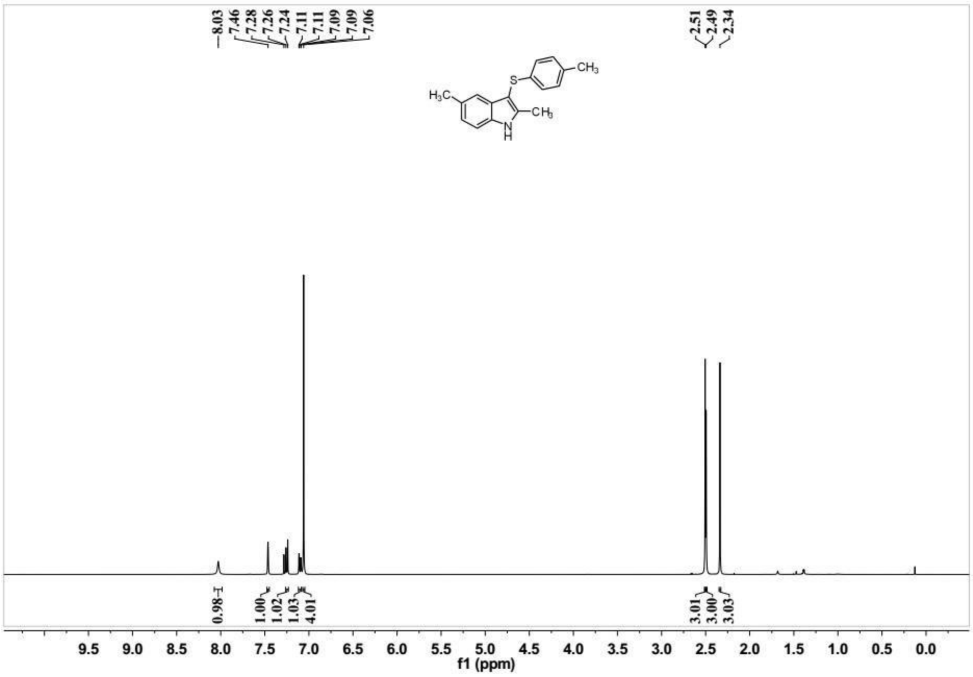


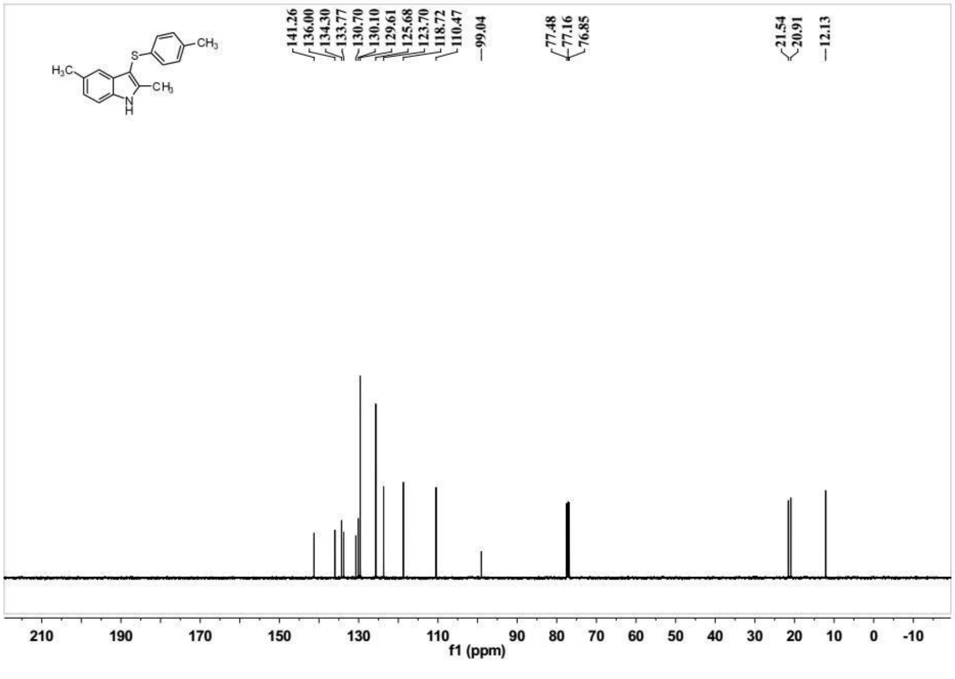

$^{1}$H and $^{13}$C NMR Spectra for **6ma**

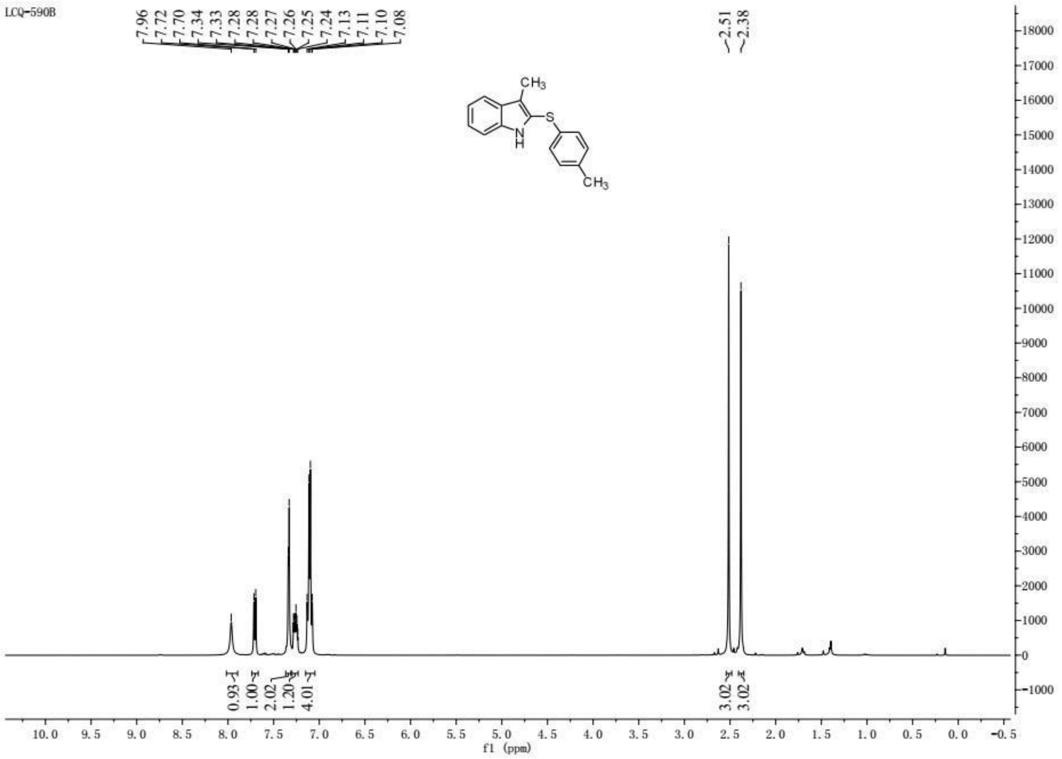


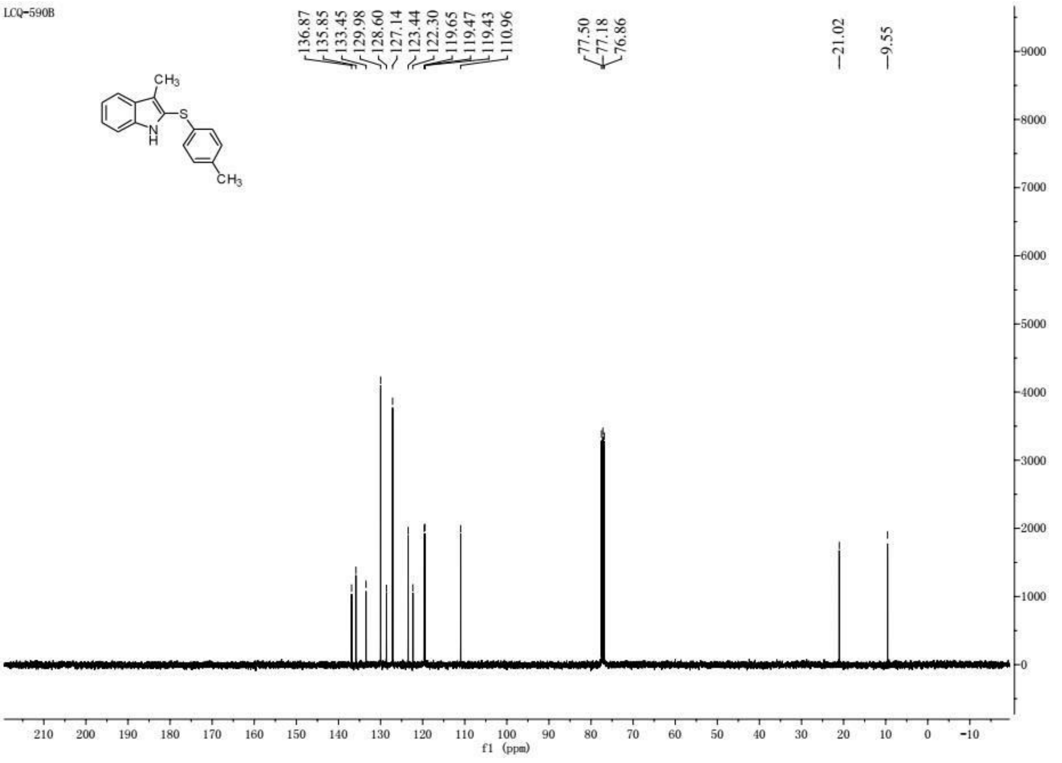

<sup></sup>$^{1}$H and $^{13}$C NMR Spectra for **6ab**

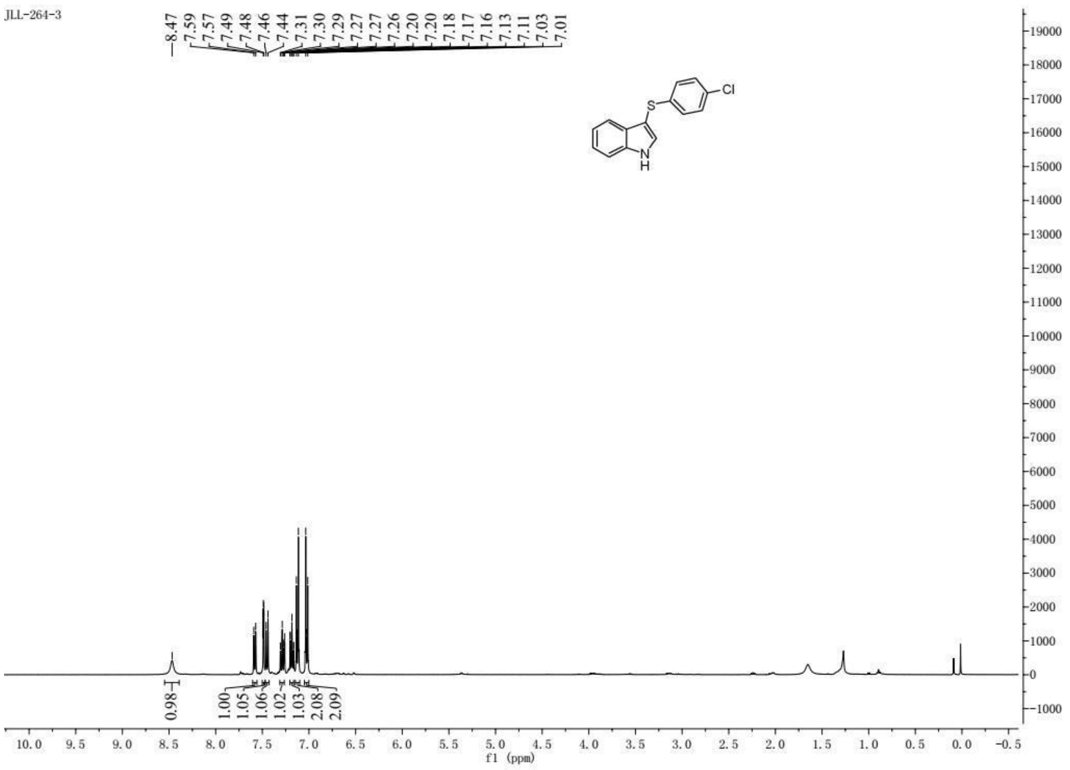


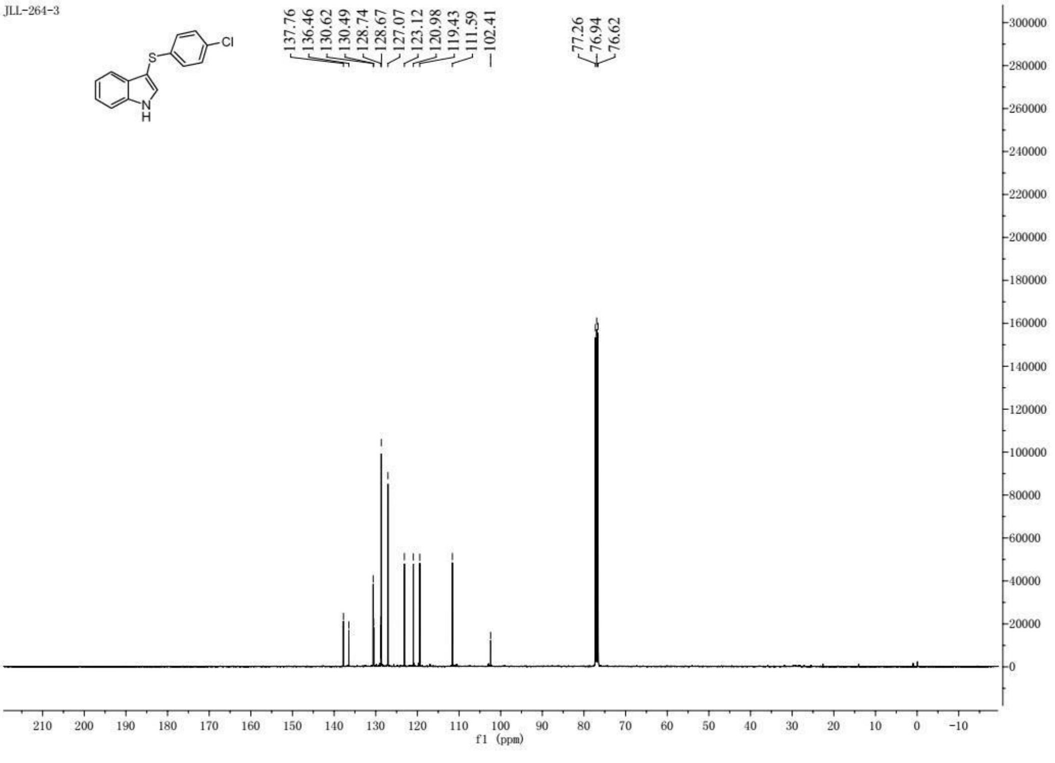

$^{1}H$ and $^{13}C$ NMR Spectra for **6ac**

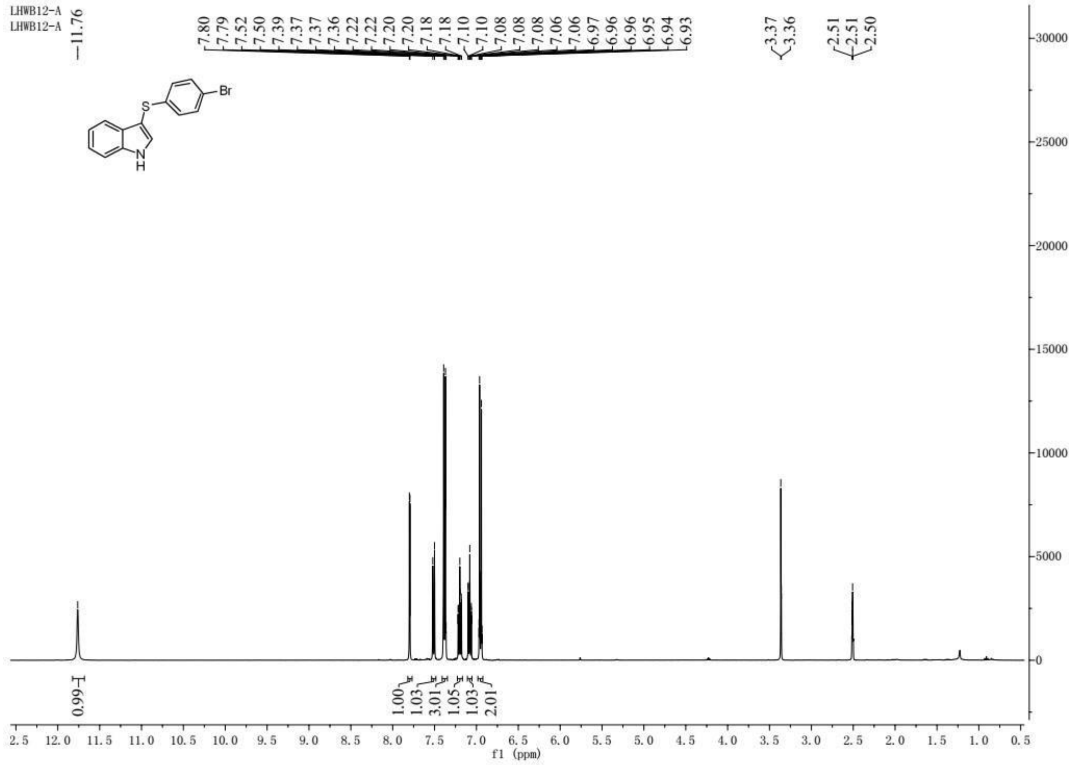


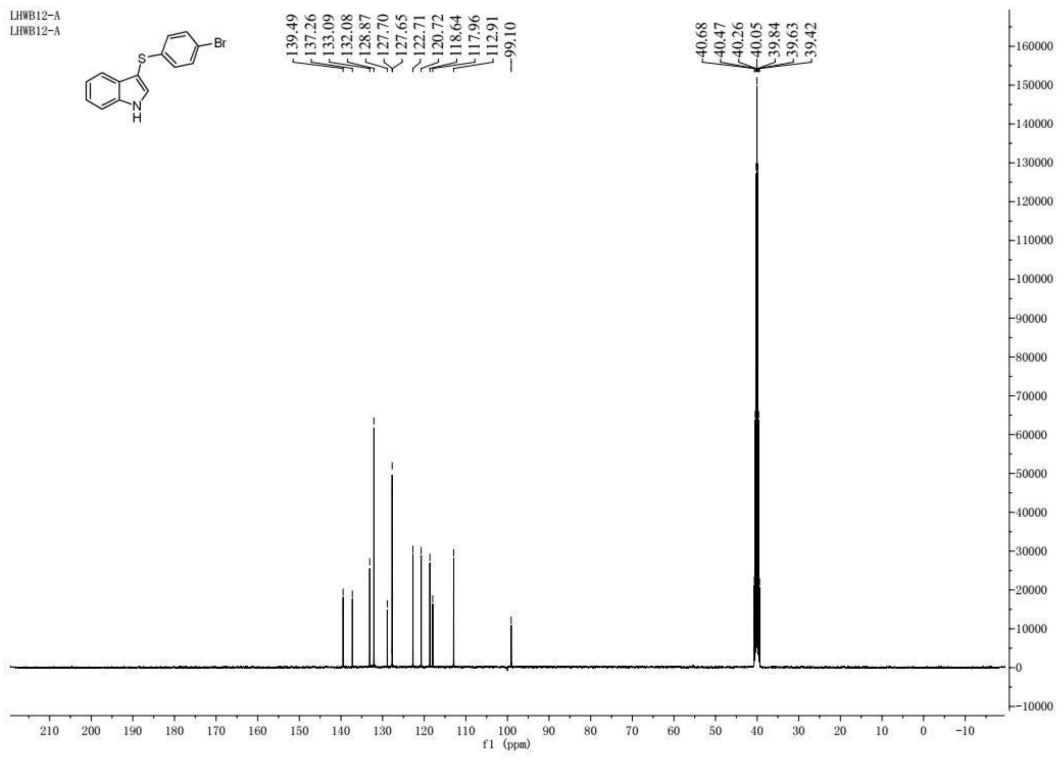

$^{1}H$ and $^{13}C$ NMR Spectra for **6ad**

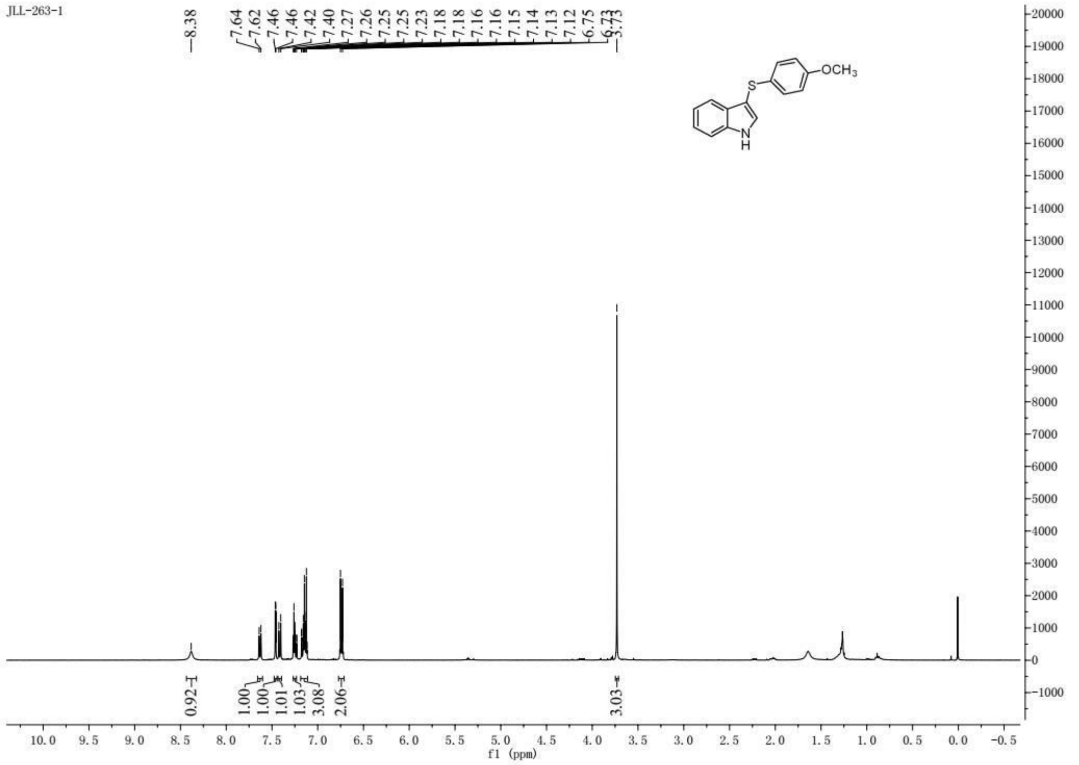


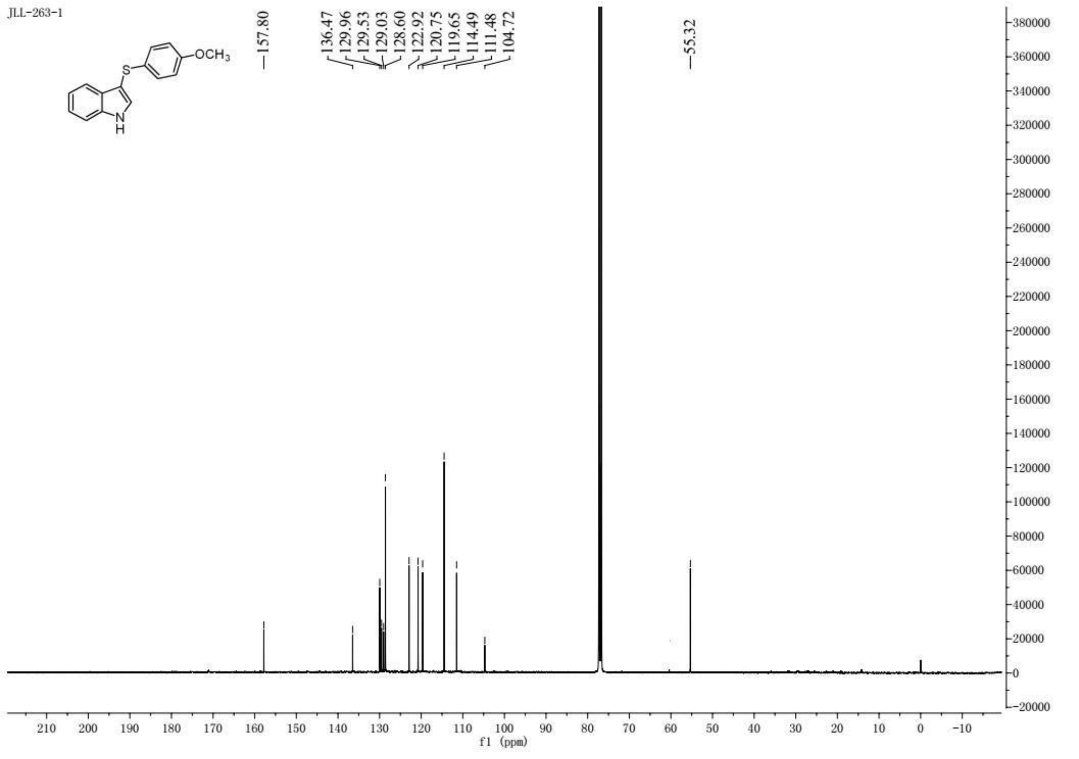

$^{1}$H and $^{13}$C NMR Spectra for **6ae**

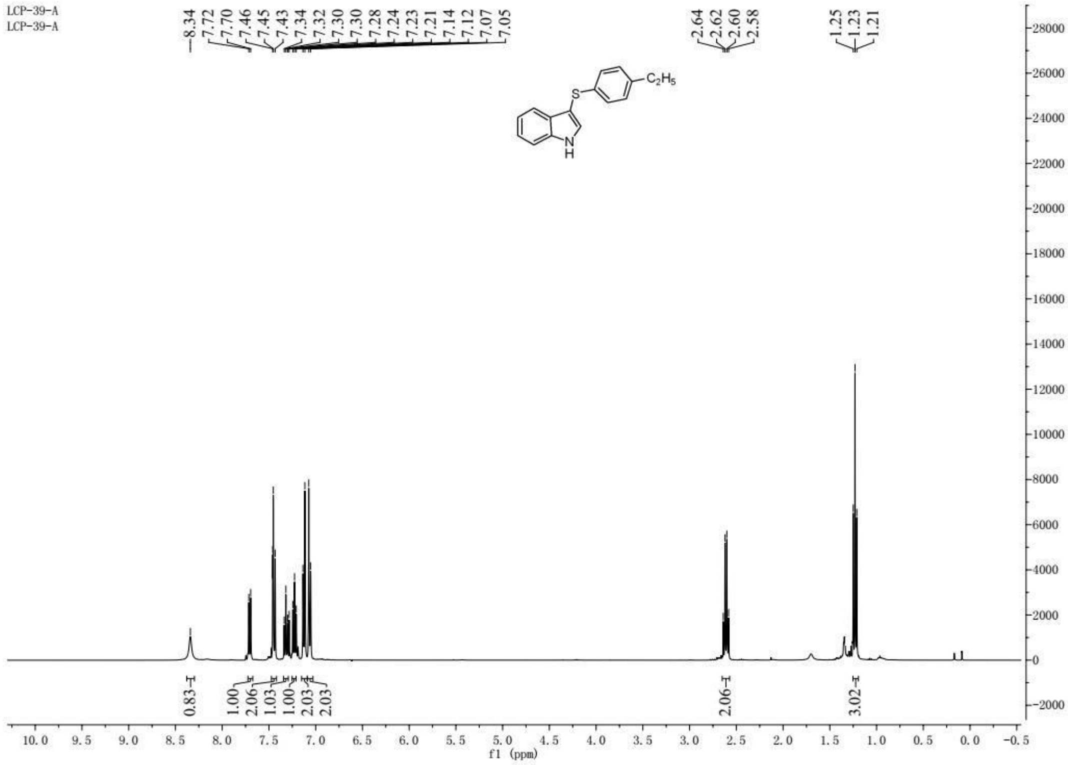


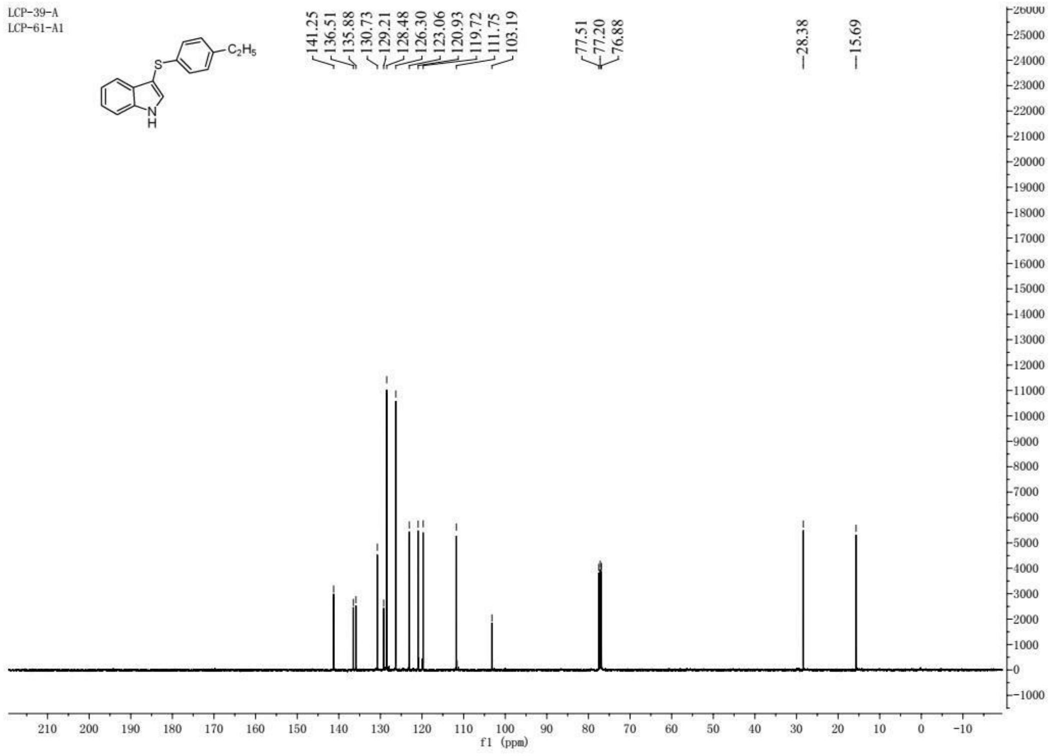

$^{1}$H and $^{13}$C NMR Spectra for **6af**

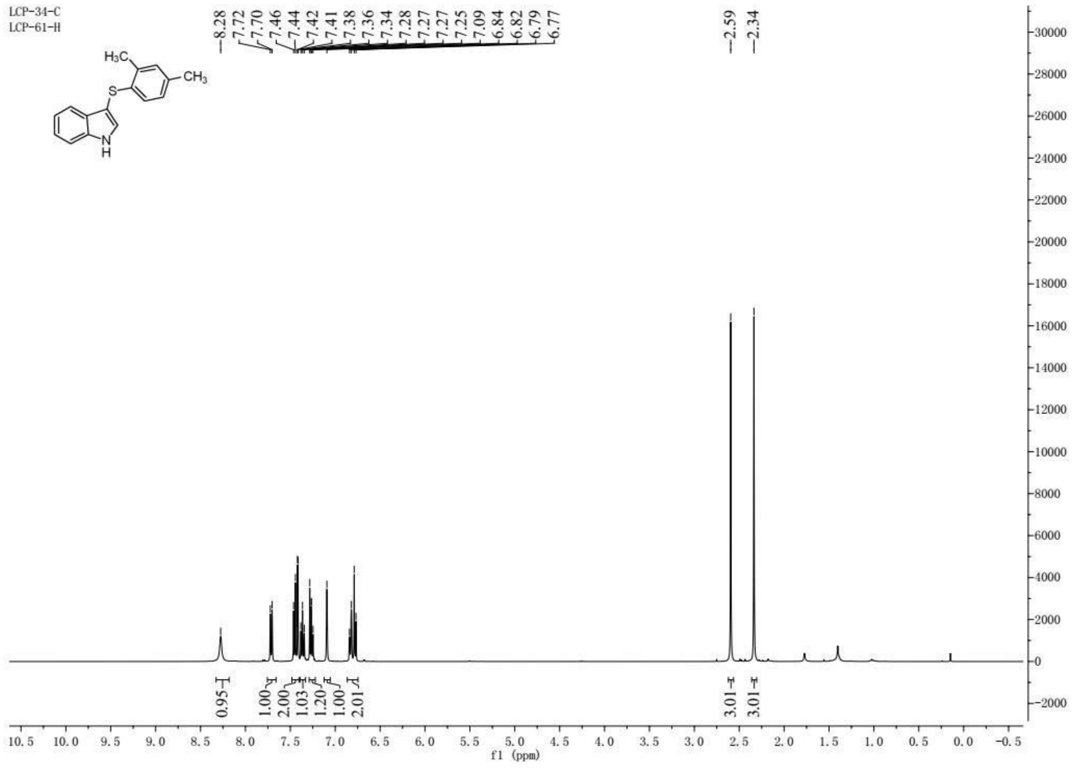


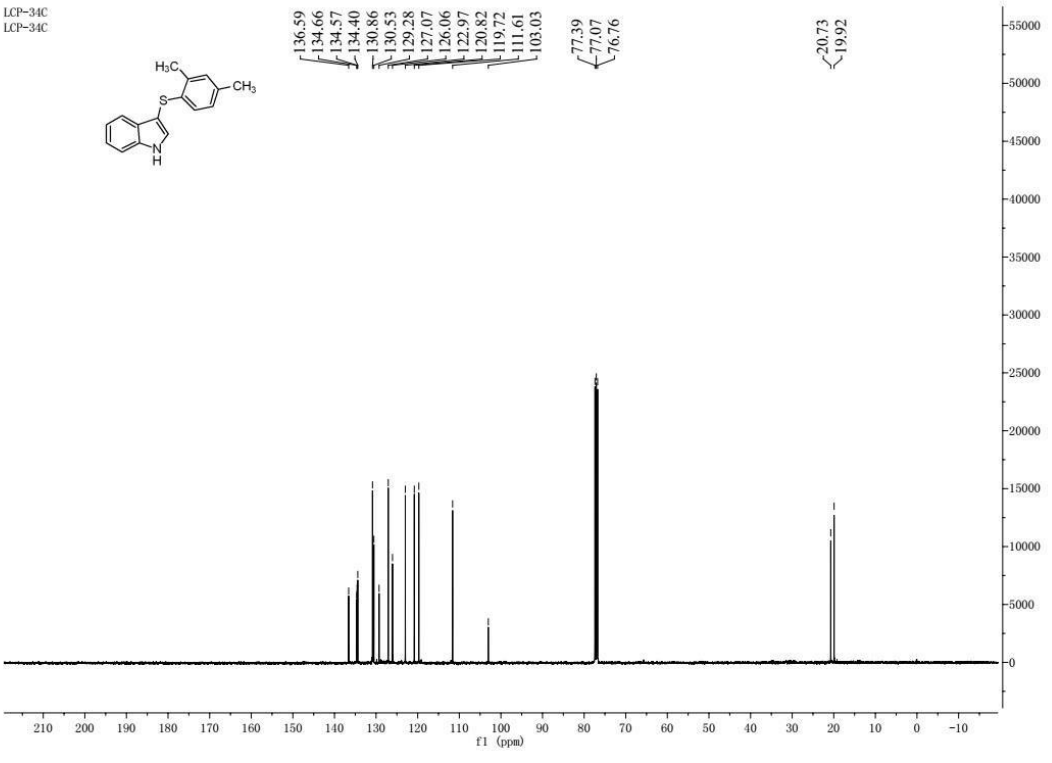

$^{1}$H and $^{13}$C NMR Spectra for **6ag**

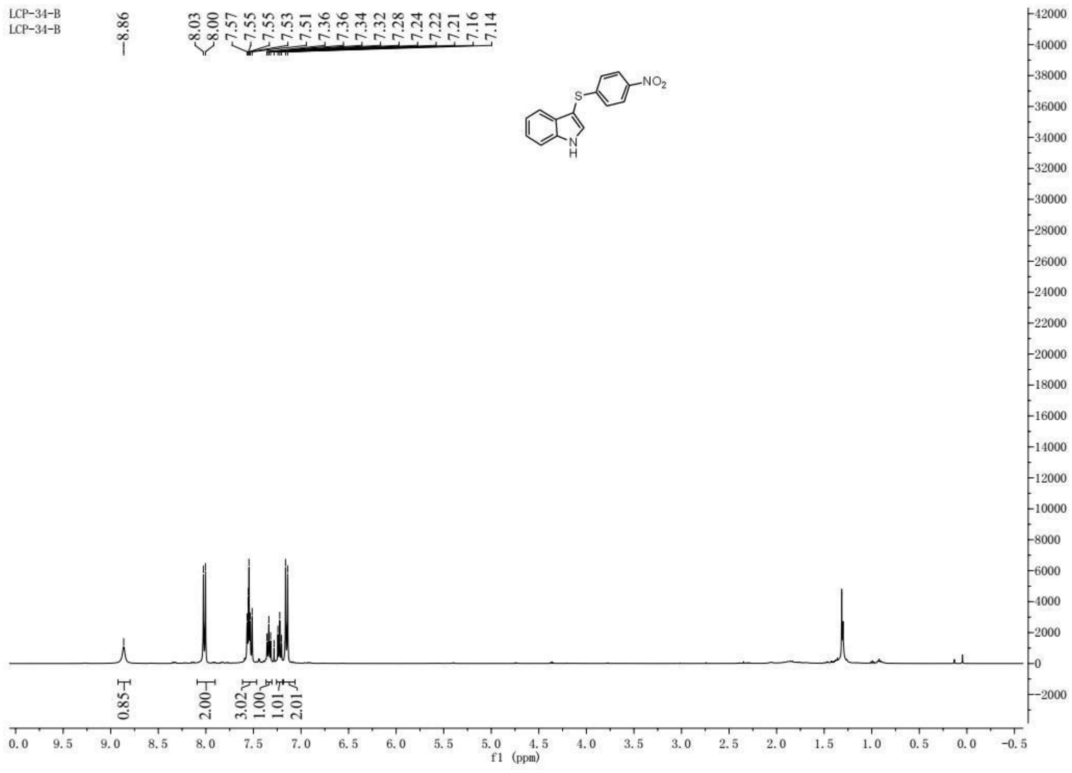


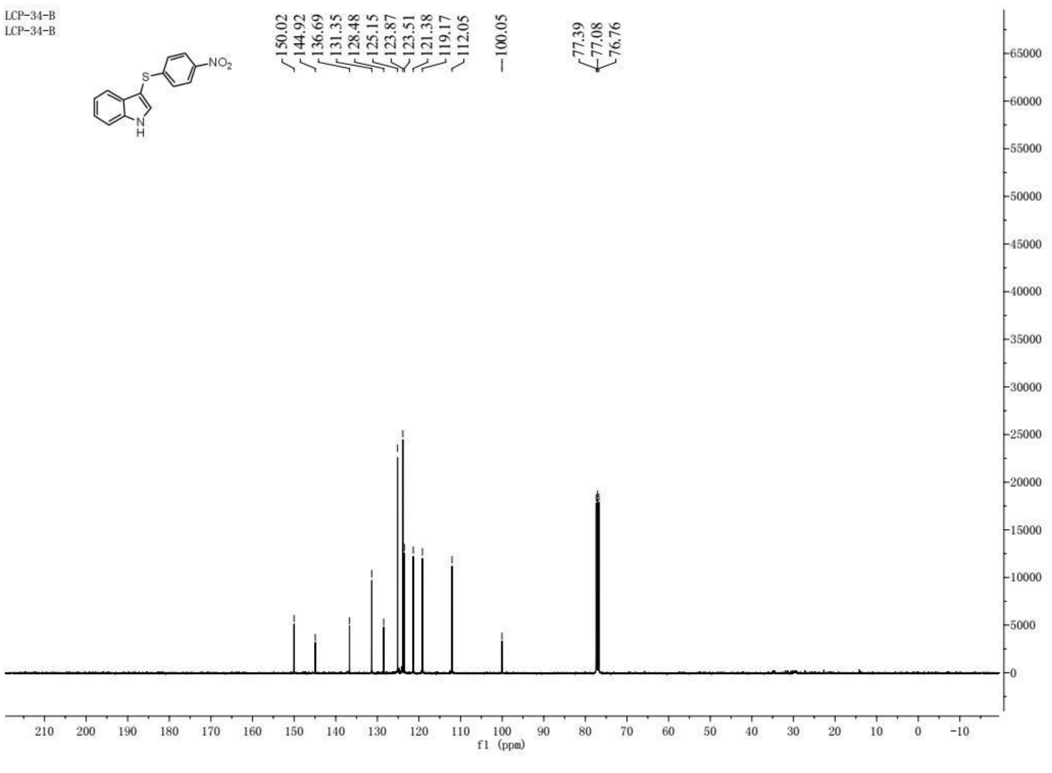

$^{1}H$ and $^{13}C$ NMR Spectra for **6ah**

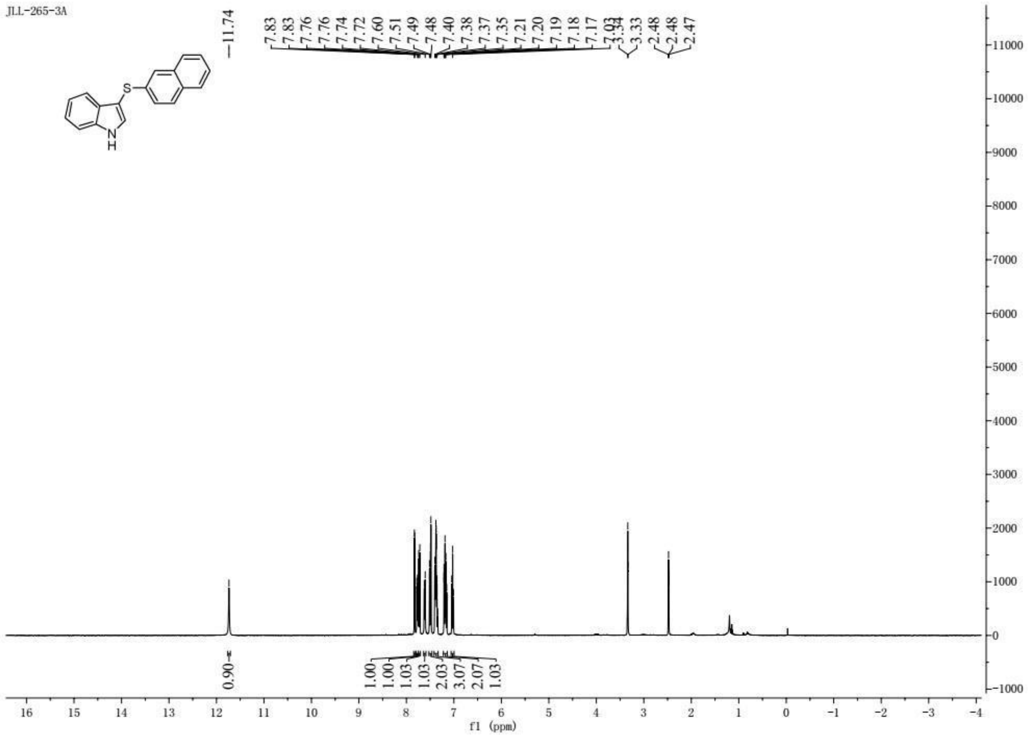


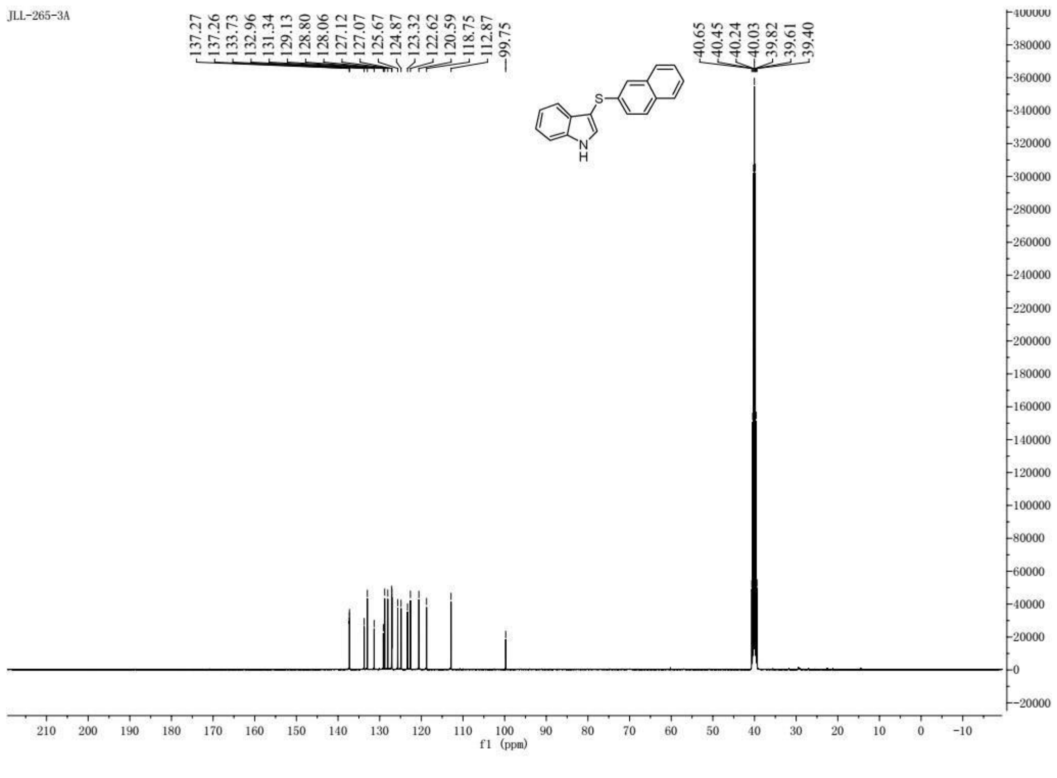

# $^{1}$H and $^{13}$C NMR Spectra for **6ai**

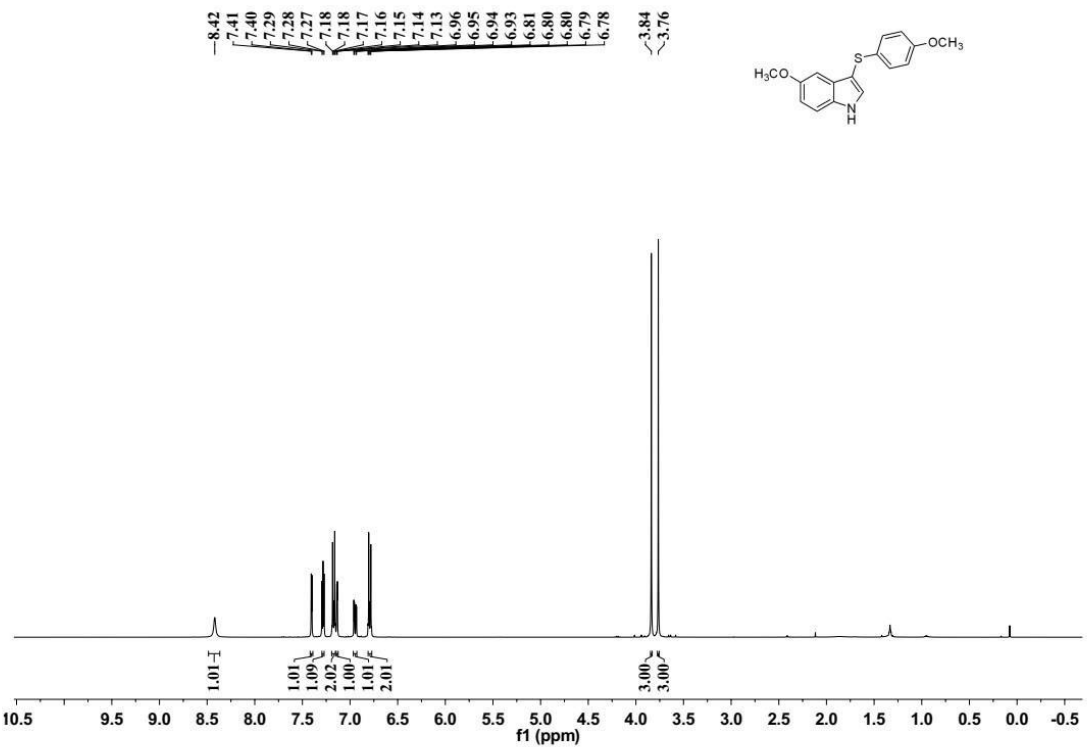


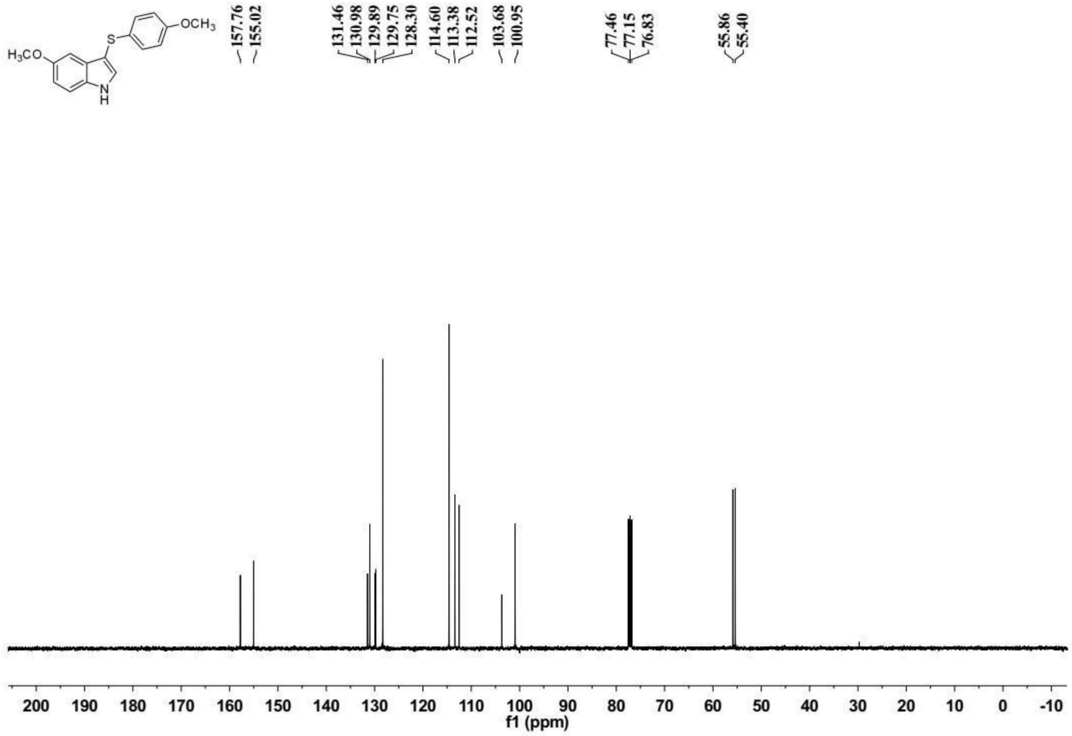

$^{1}$H and $^{13}$C NMR Spectra for **6aj**

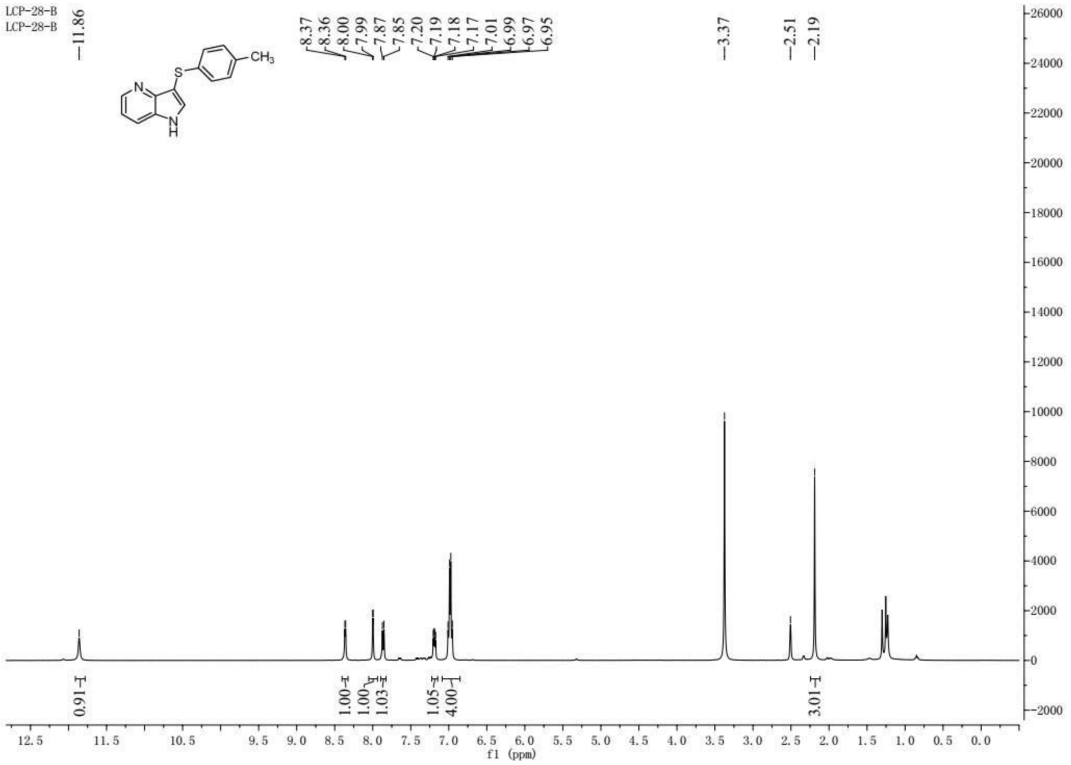


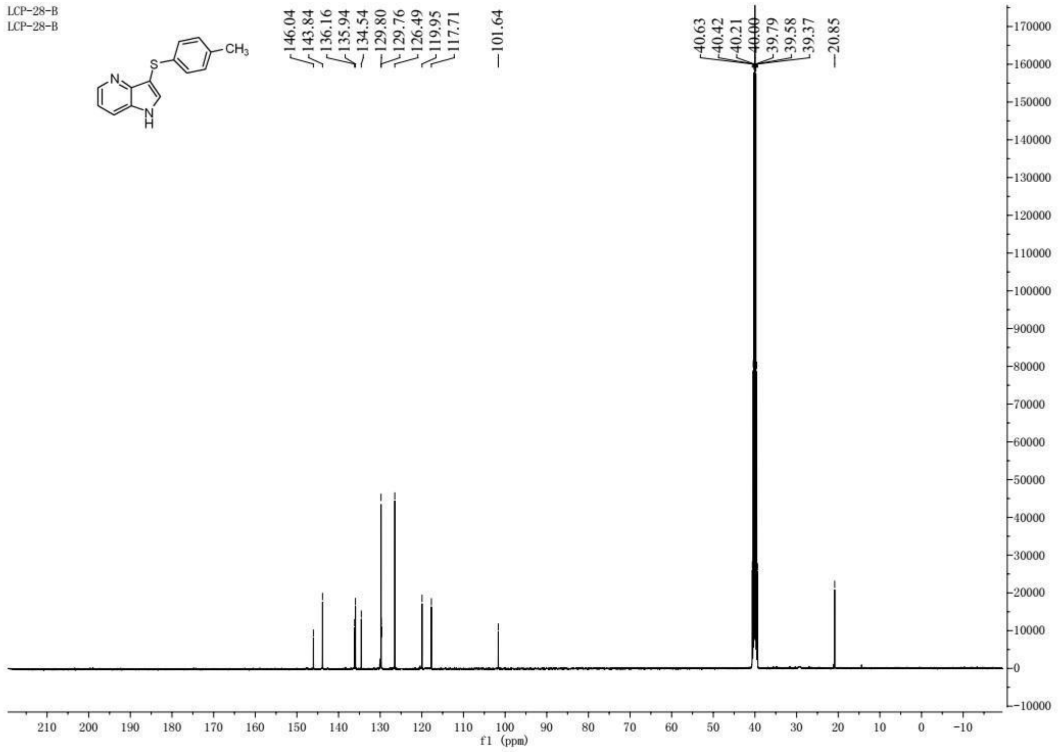

$^{1}H$ and $^{13}C$ NMR Spectra for **6ak**

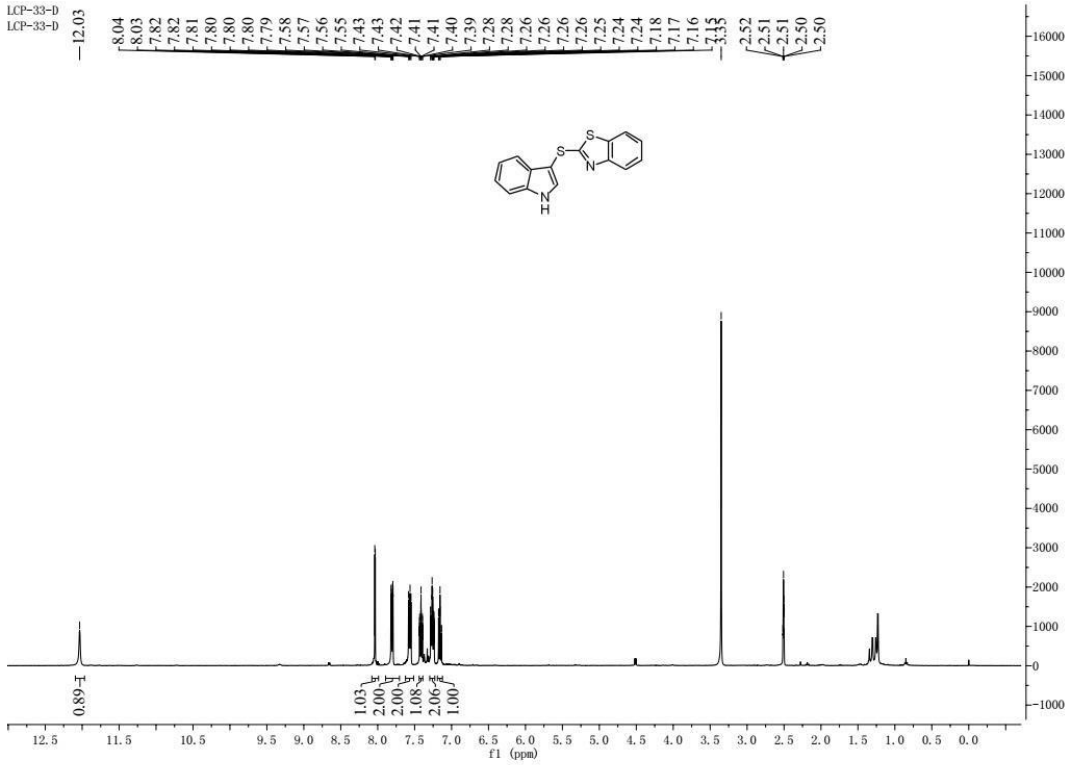

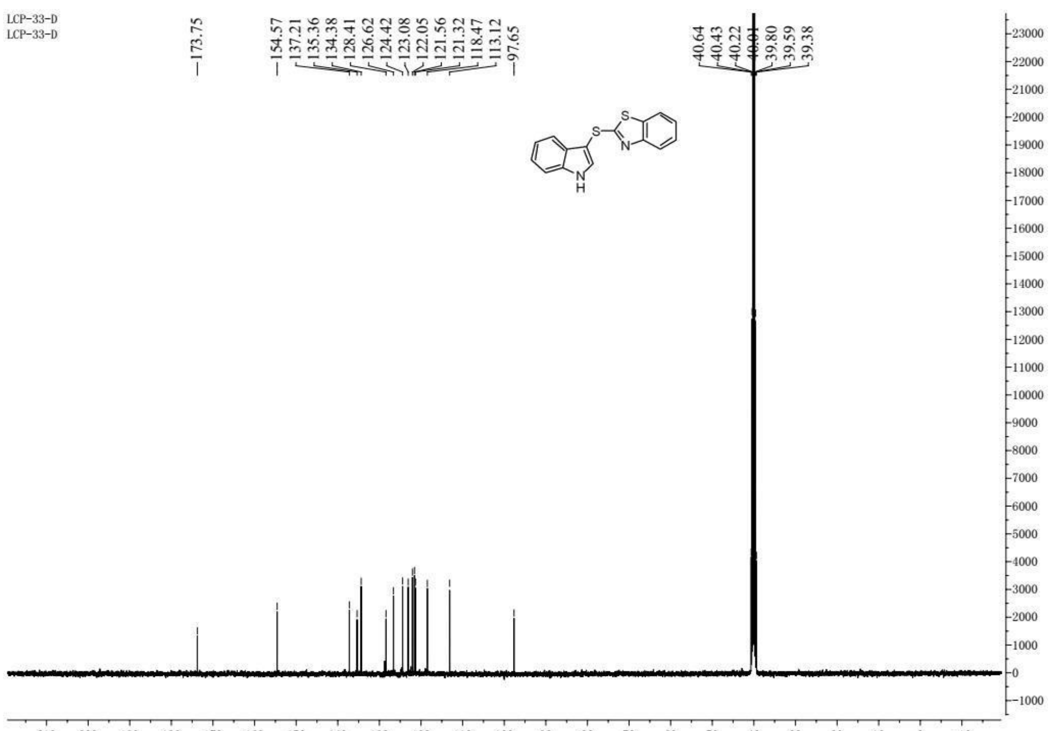

$^{1}$H and $^{13}$C NMR Spectra for **6al**

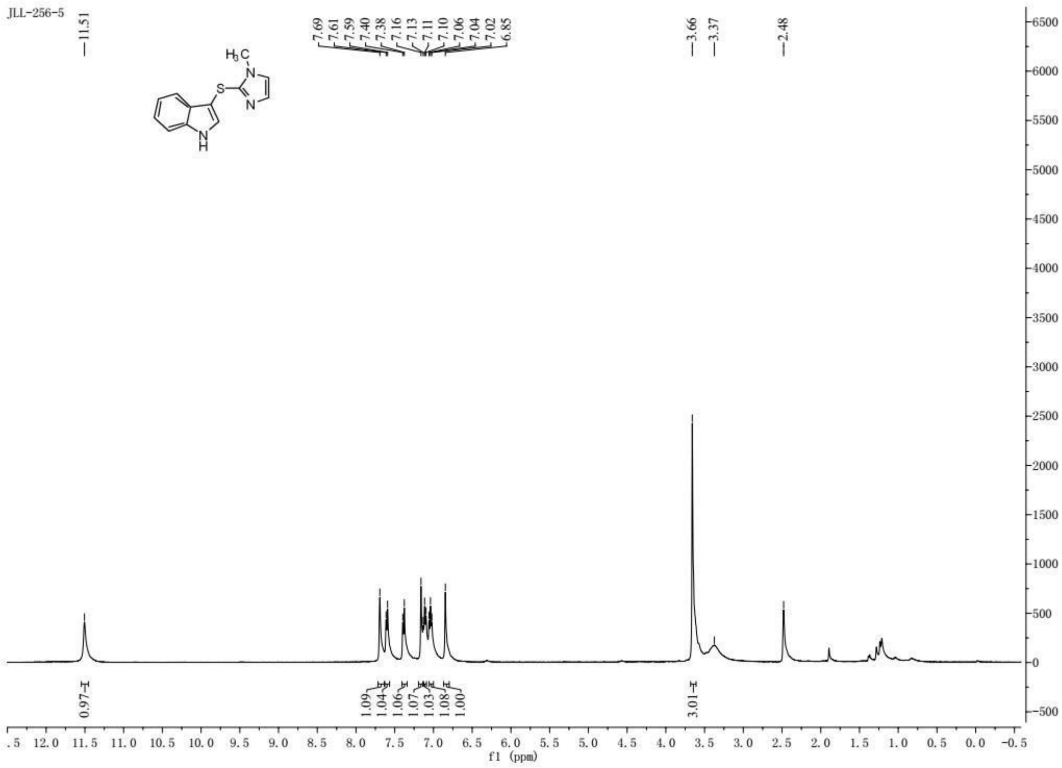


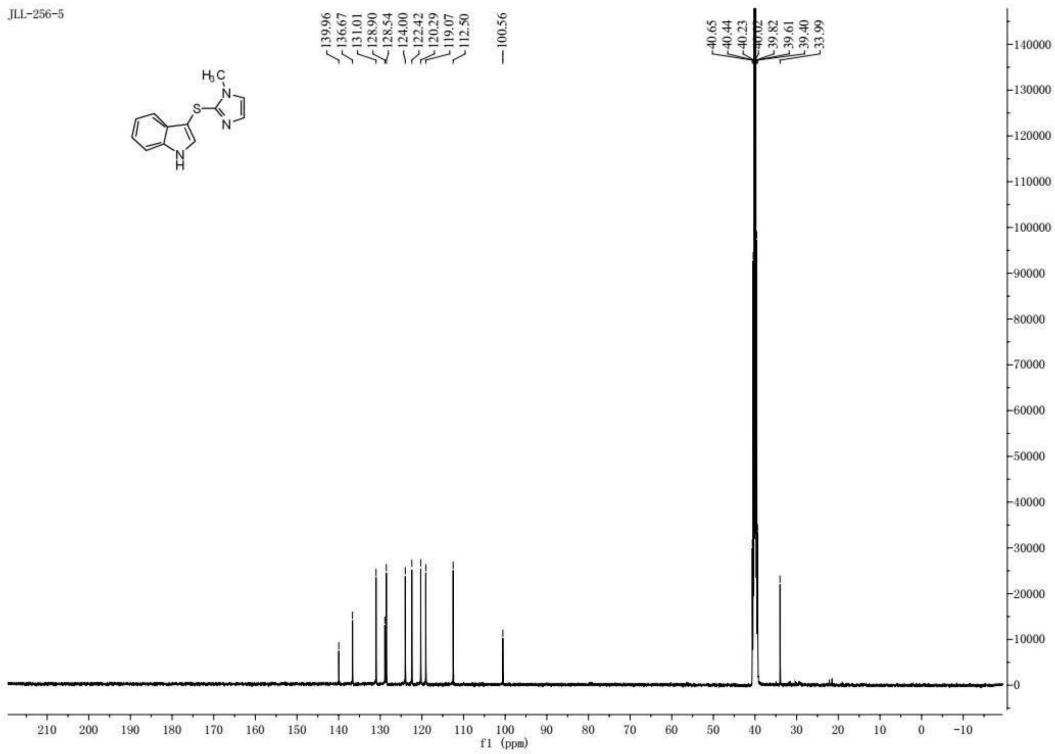

$^{1}H$ and $^{13}C$ NMR Spectra for **6am**

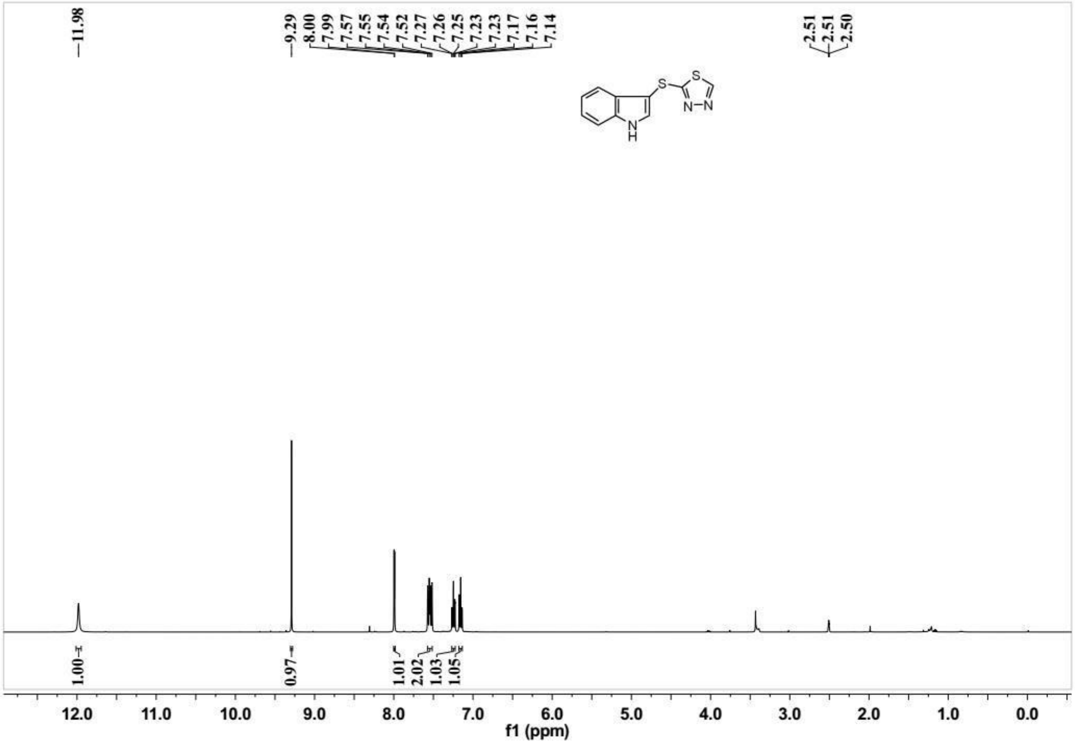


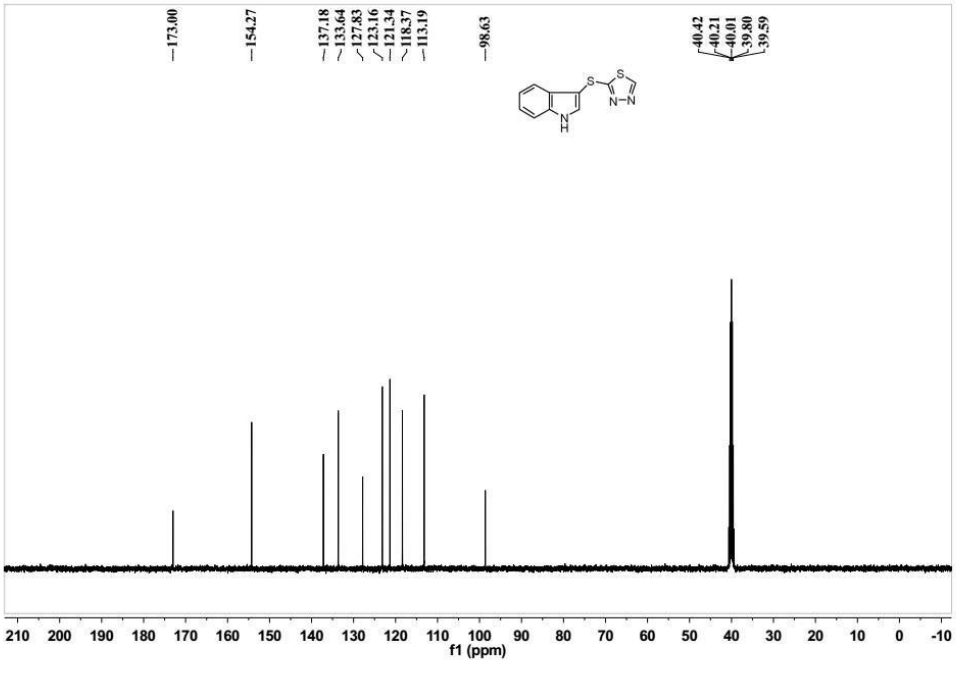

$^{1}$H and $^{13}$C NMR Spectra for **6an**

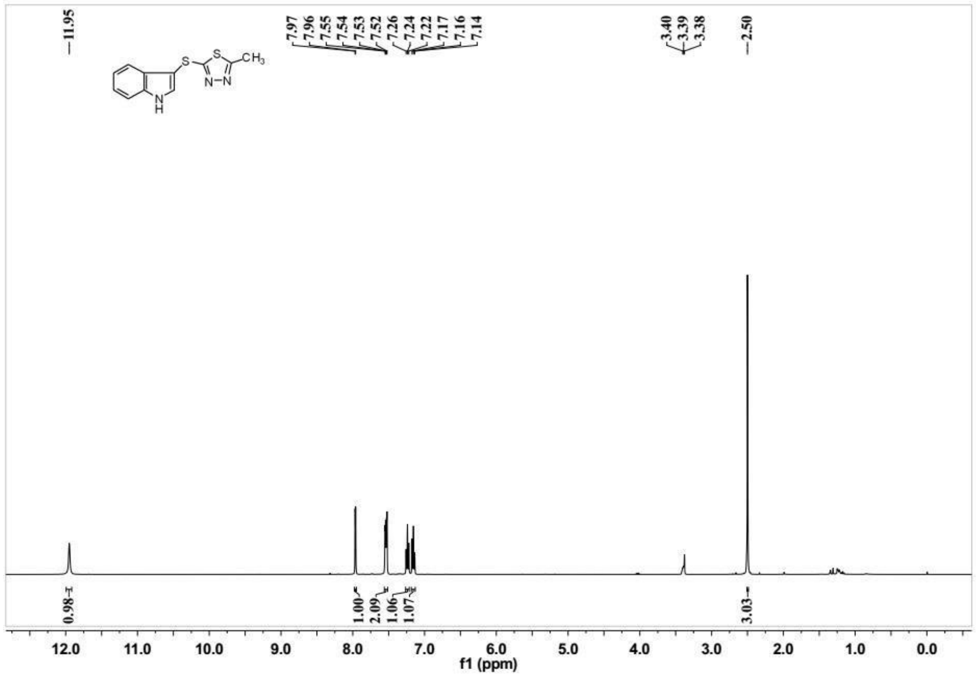

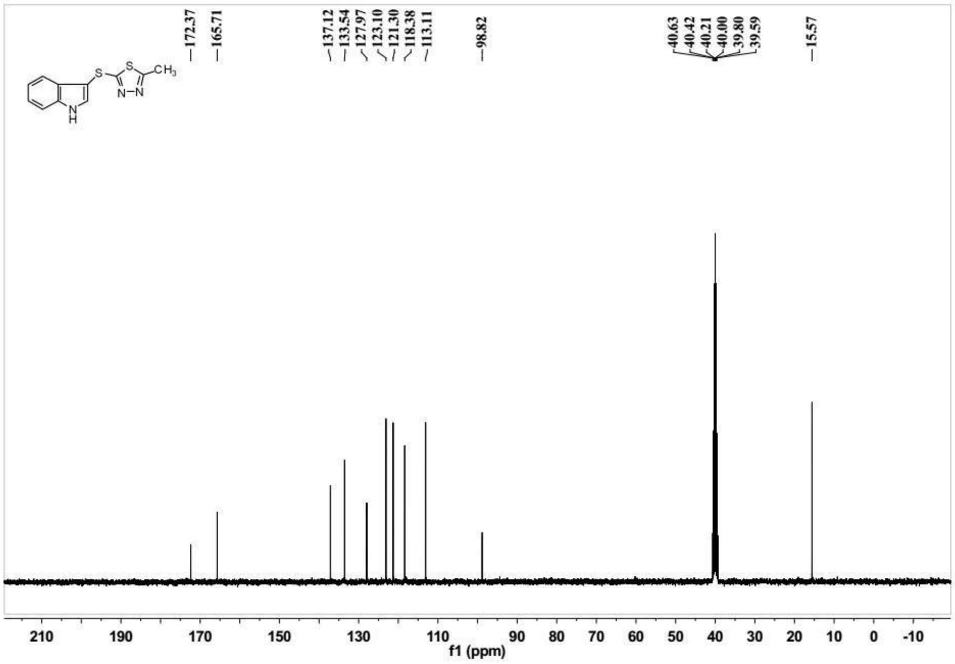

<sup></sup>$^{1}$H and $^{13}$C NMR Spectra for **6ao**

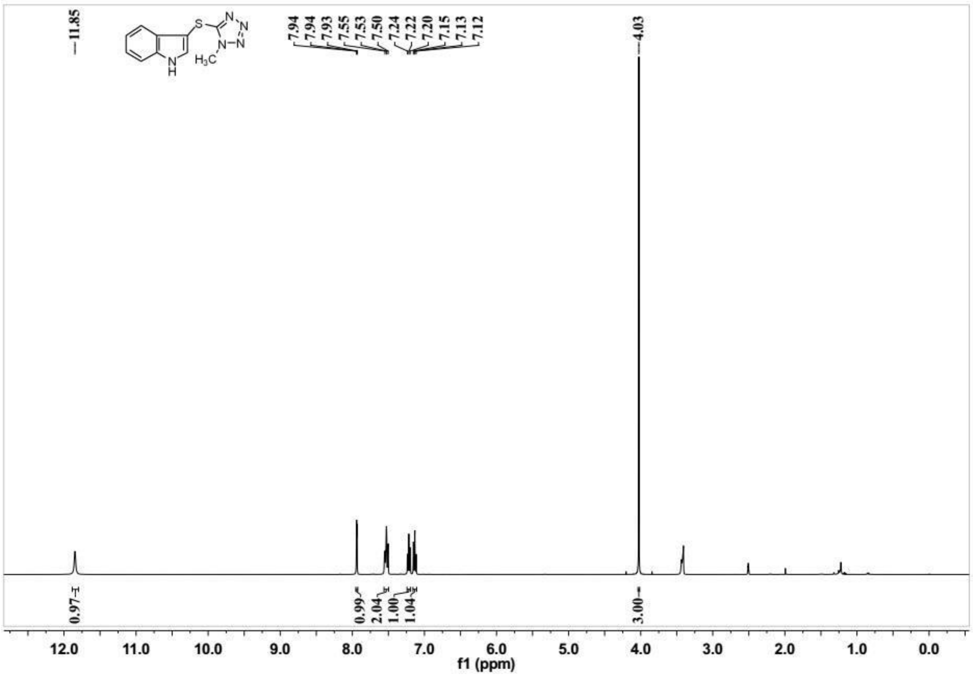


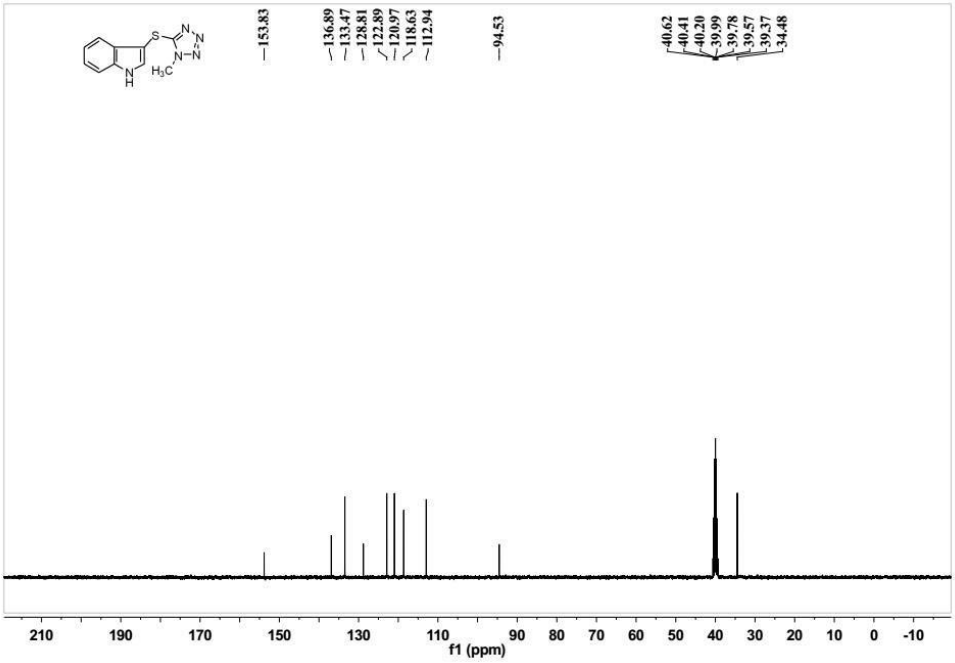

$^{1}H$ and $^{13}C$ NMR Spectra for **7a**

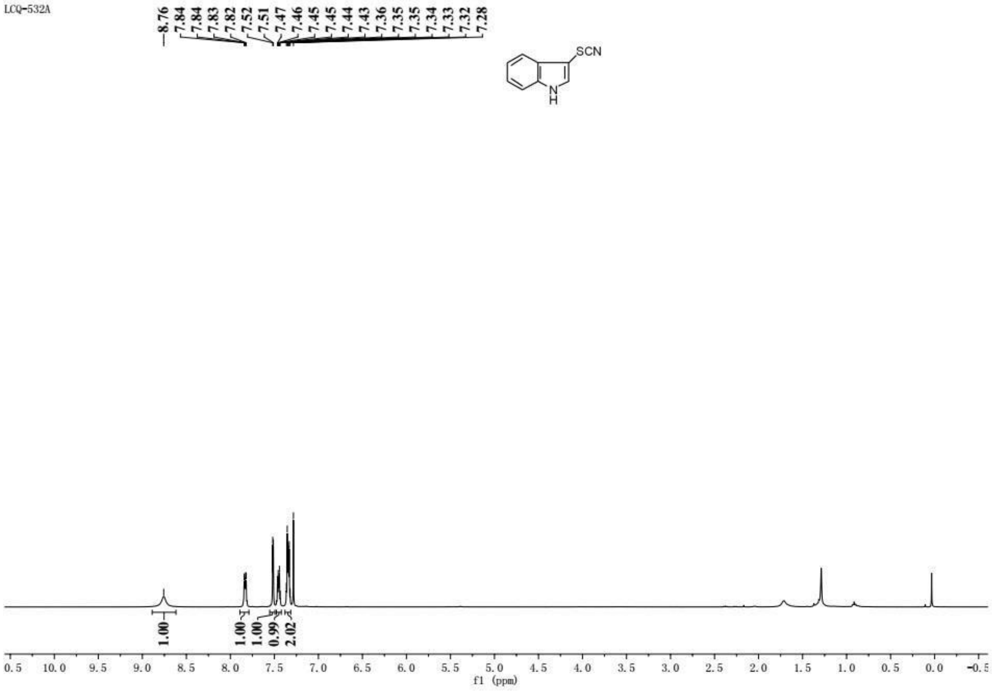


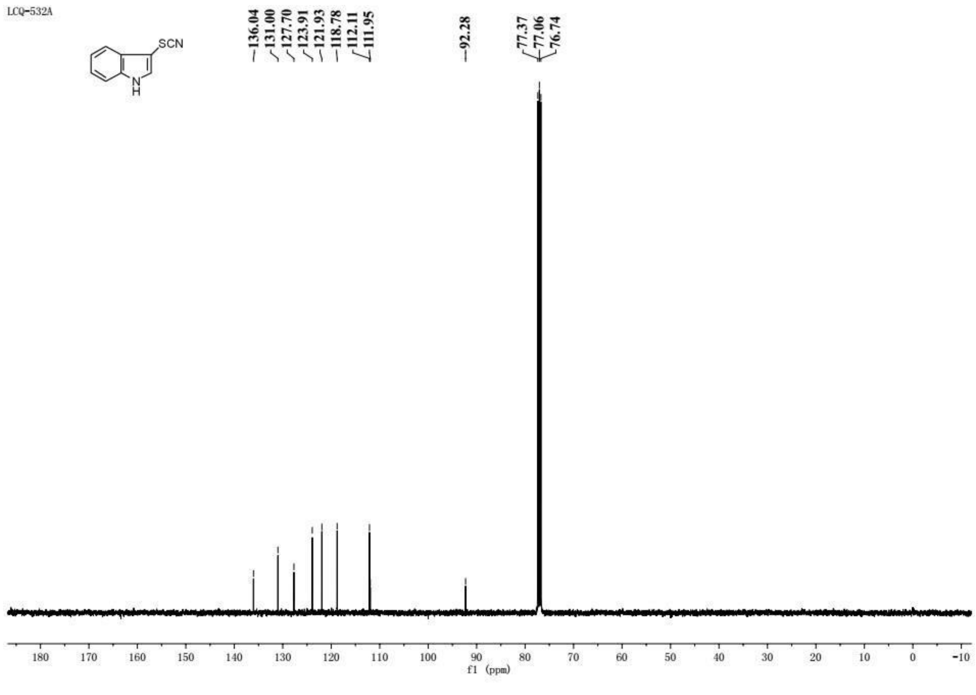

$^{1}$H and $^{13}$C NMR Spectra for **7b**

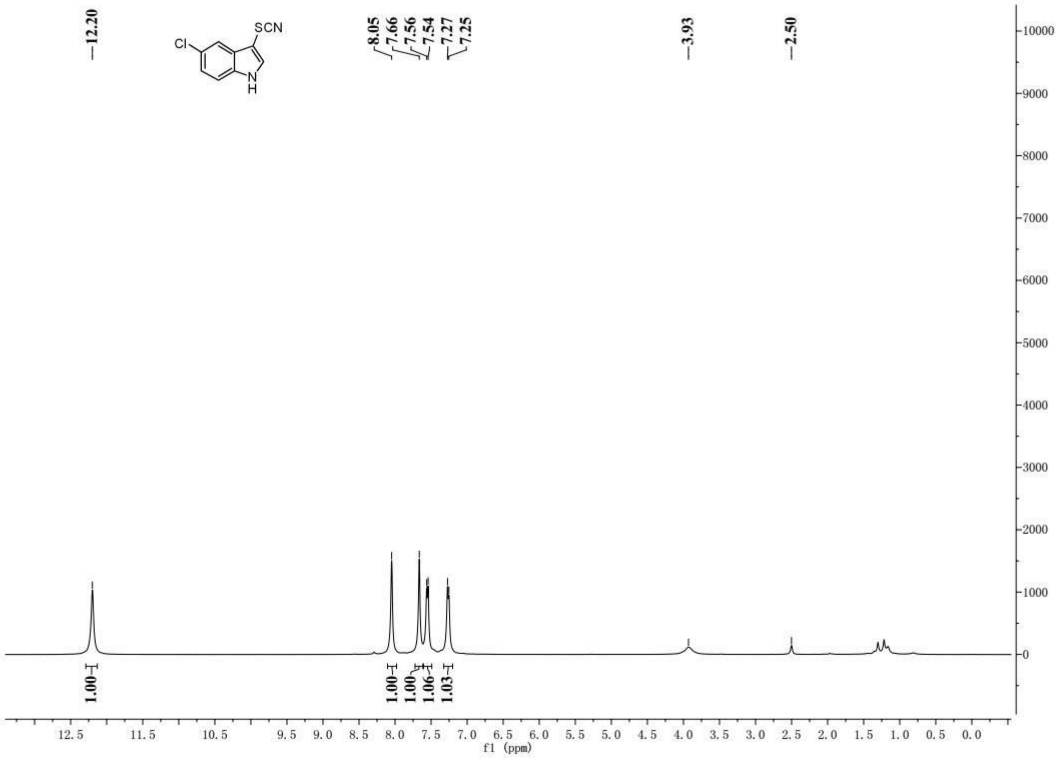


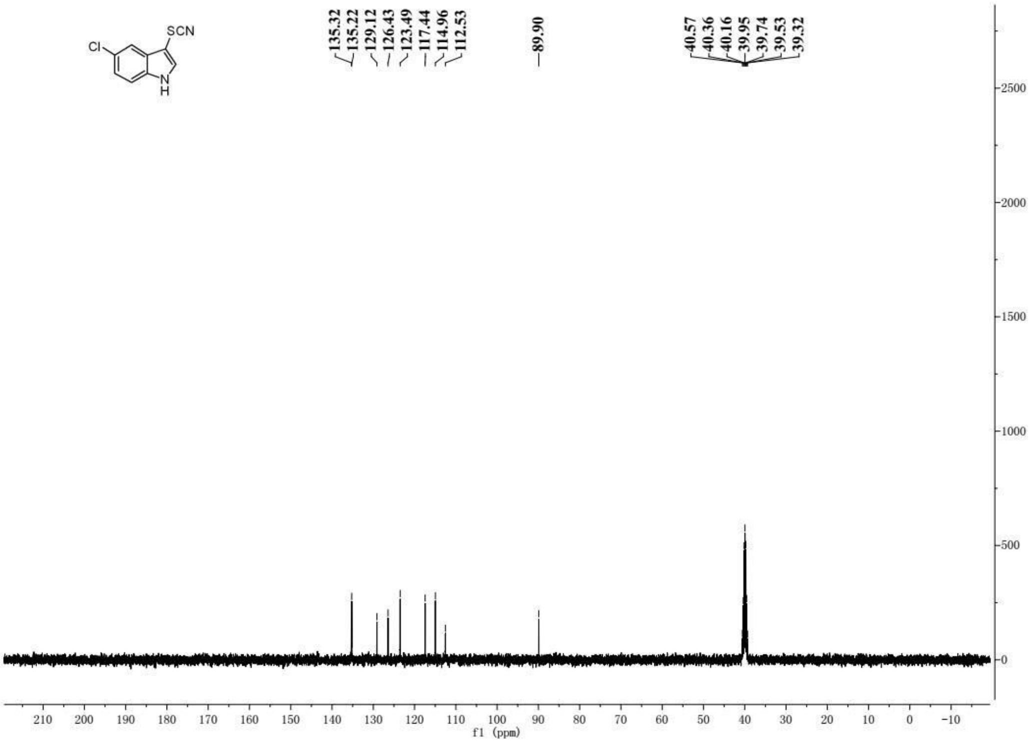

<sup></sup>

$^{1}$H and $^{13}$C NMR Spectra for **7c**

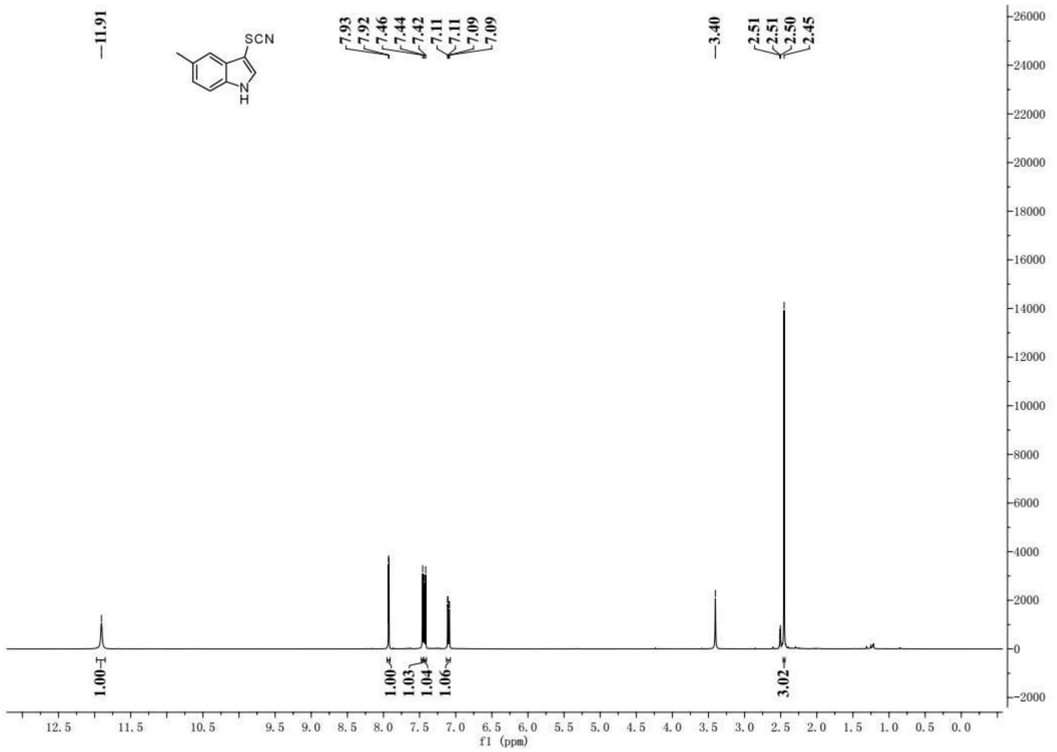


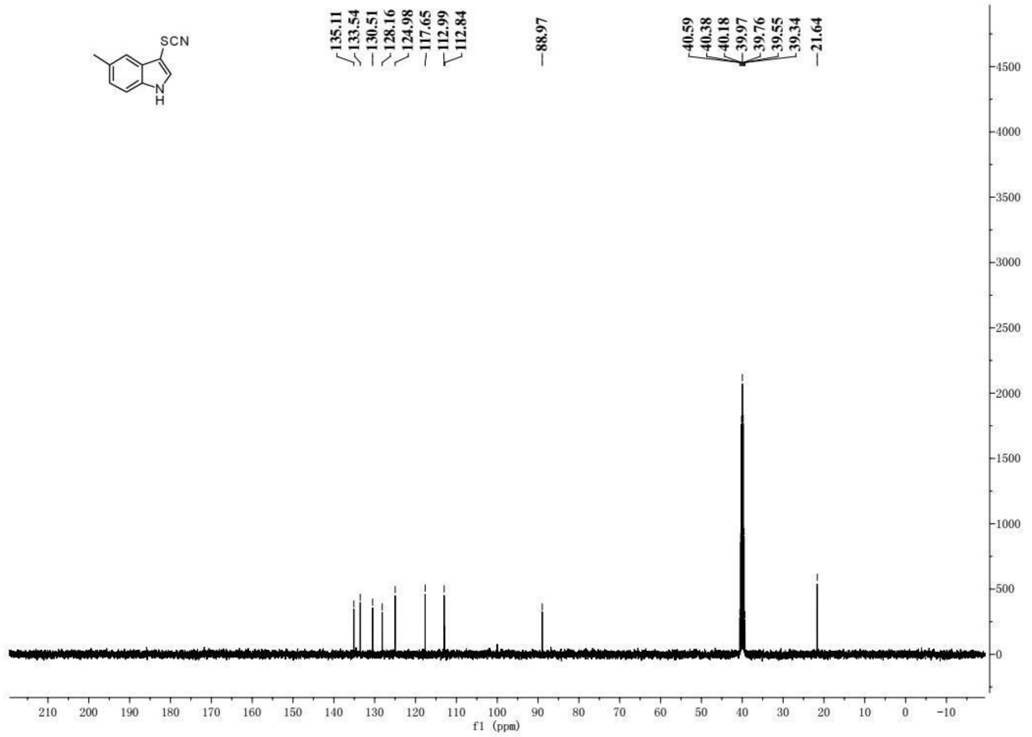

$^{1}H$ and $^{13}C$ NMR Spectra for **7d**

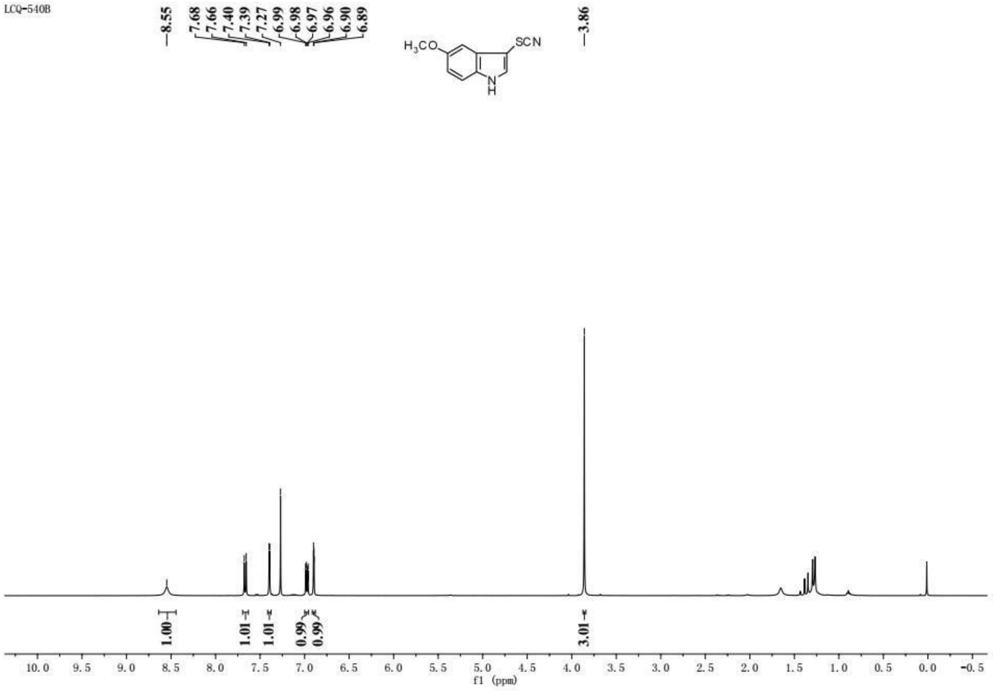


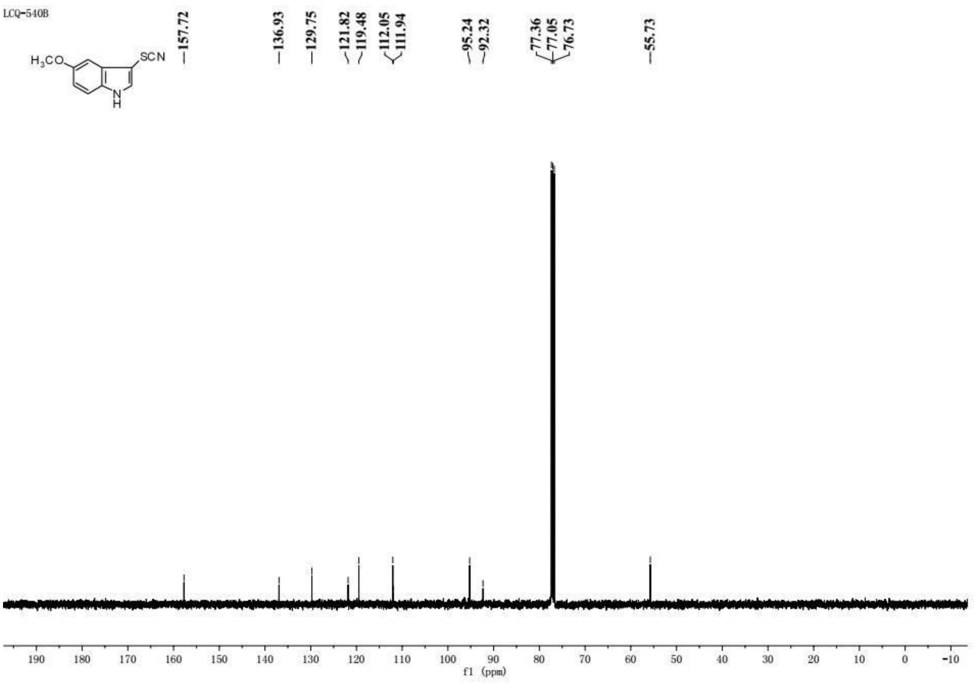

$^{1}H$ and $^{13}C$ NMR Spectra for **7e**

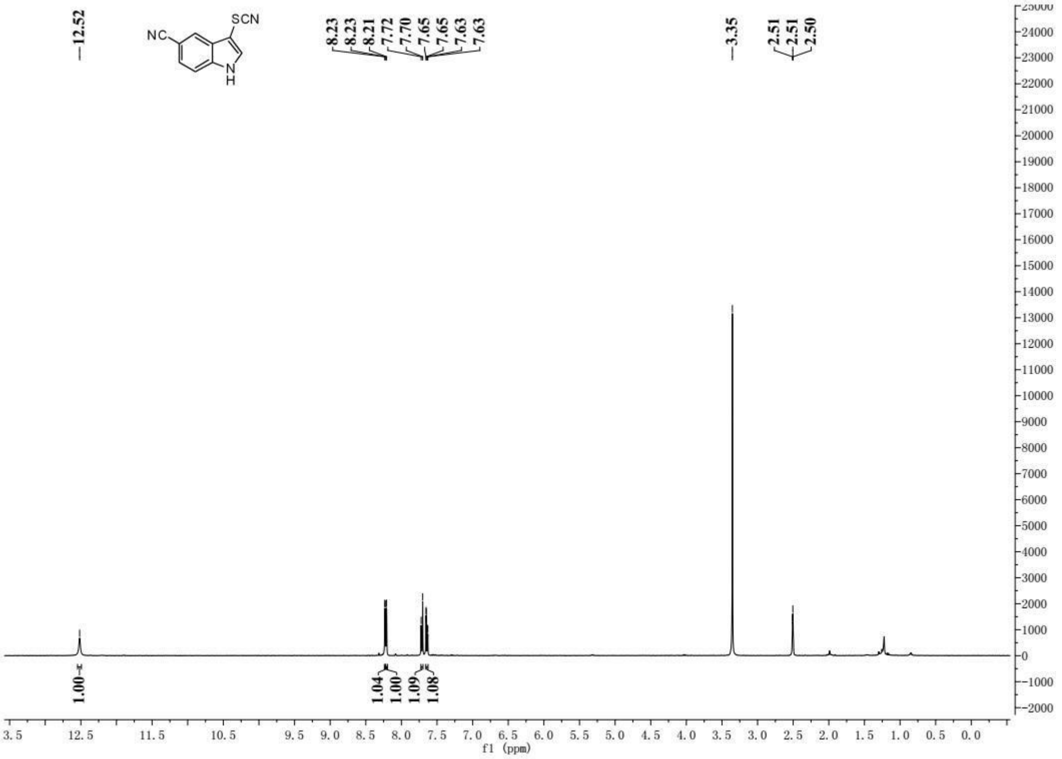


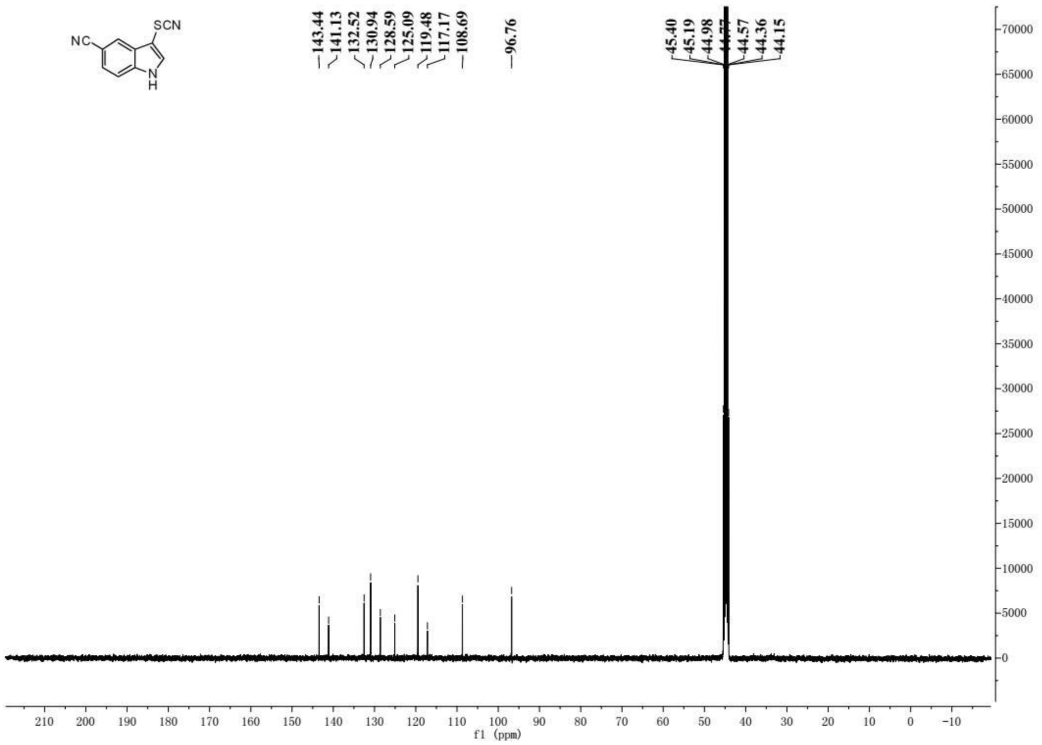

$^{1}H$ and $^{13}C$ NMR Spectra for **7f**

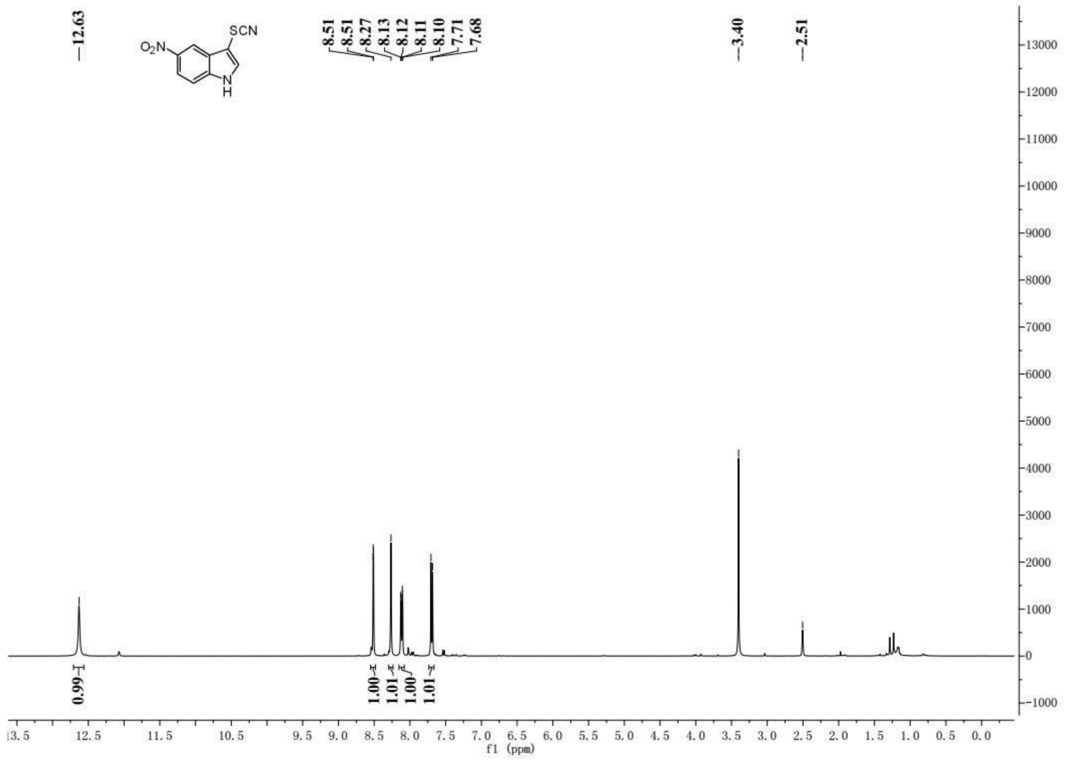

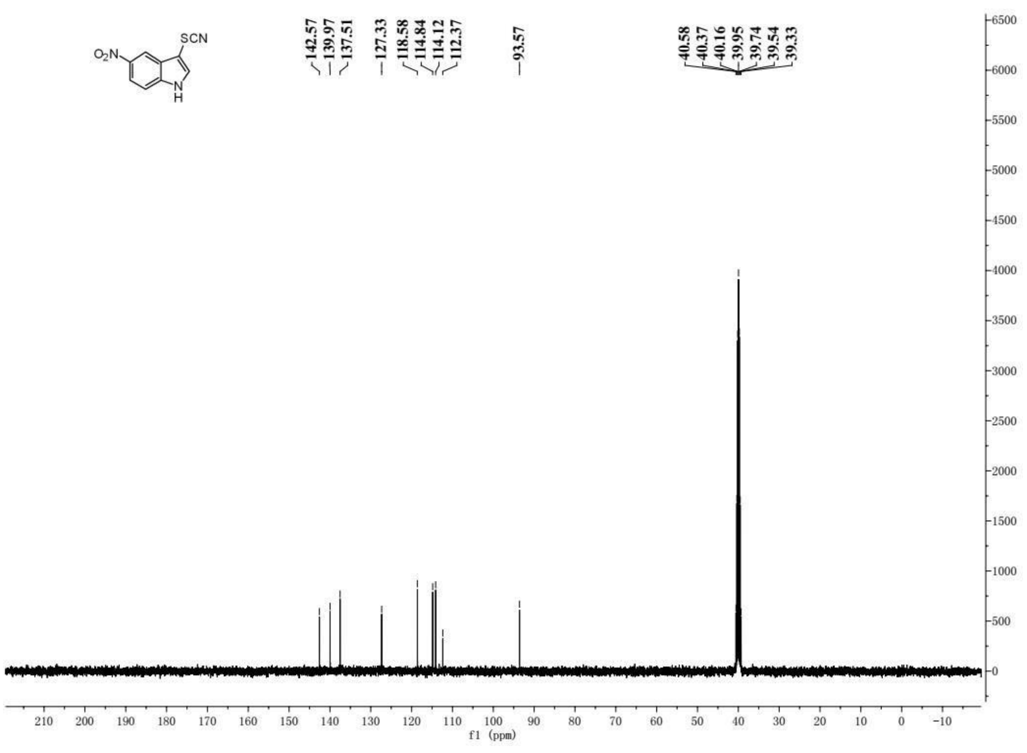

$^{1}$H and $^{13}$C NMR Spectra for **7g**

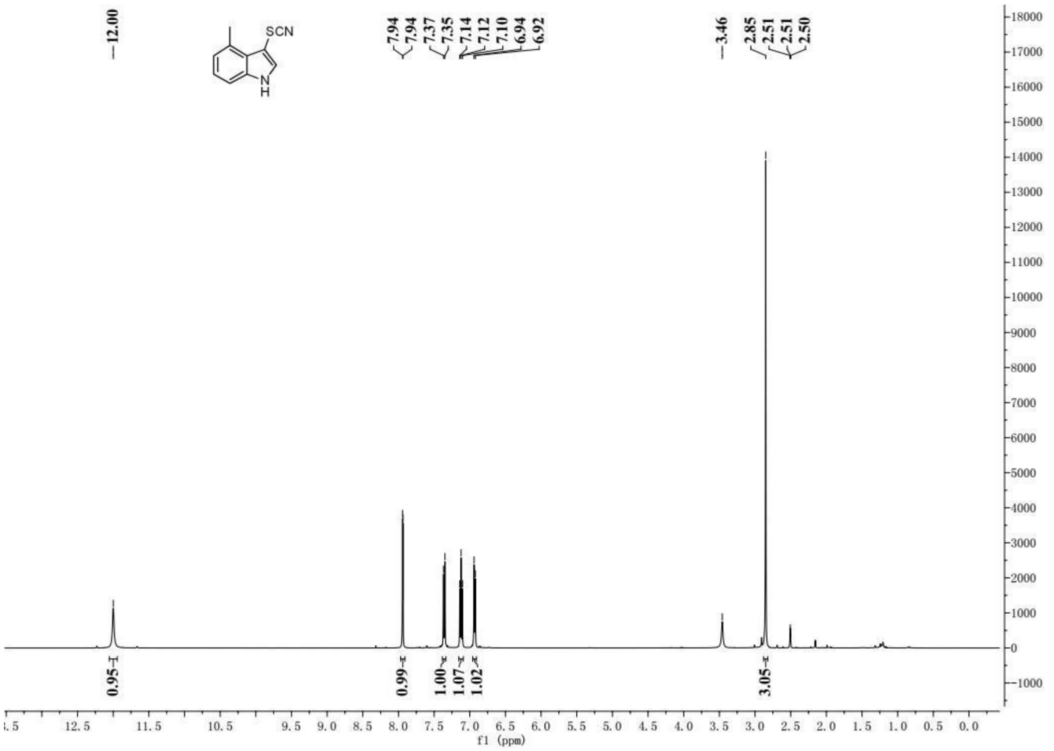

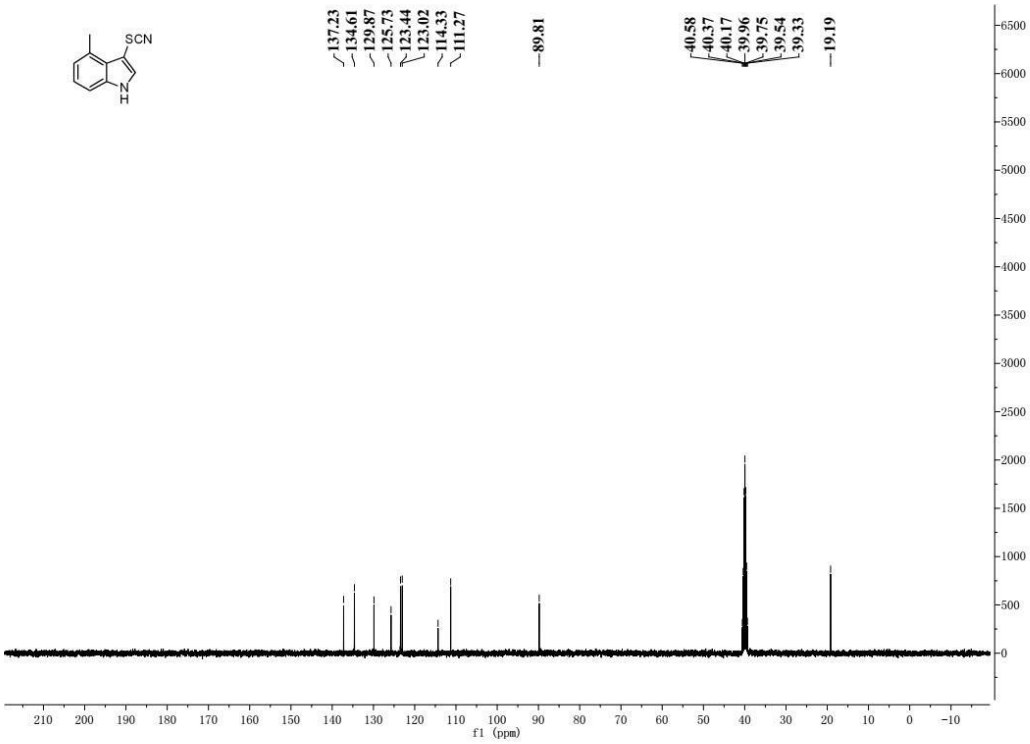

<sup></sup>$^{1}$H and $^{13}$C NMR Spectra for **7h**

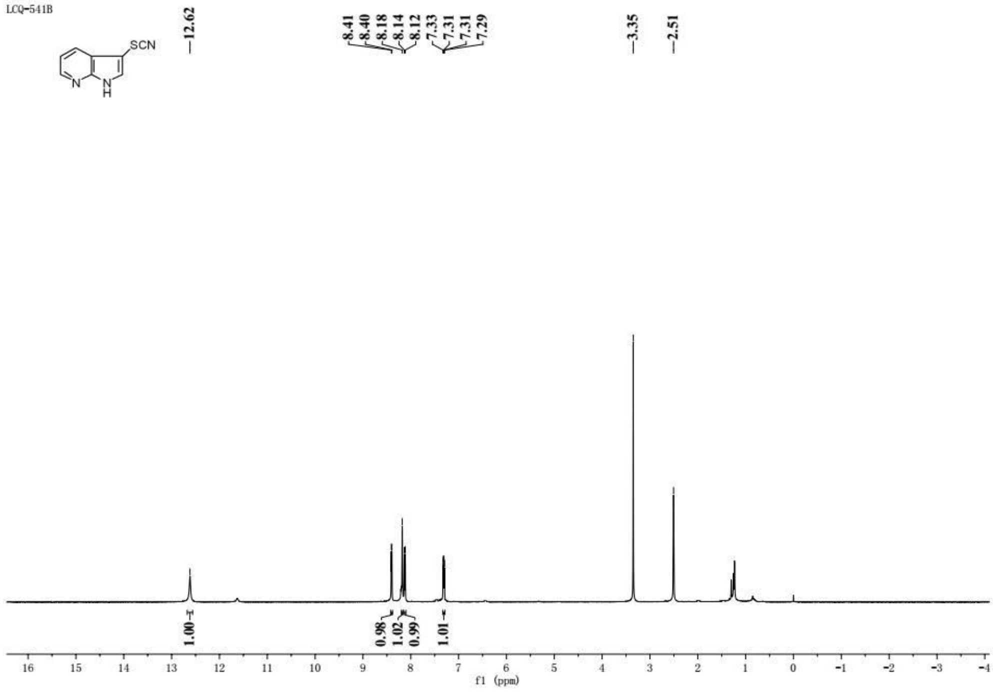


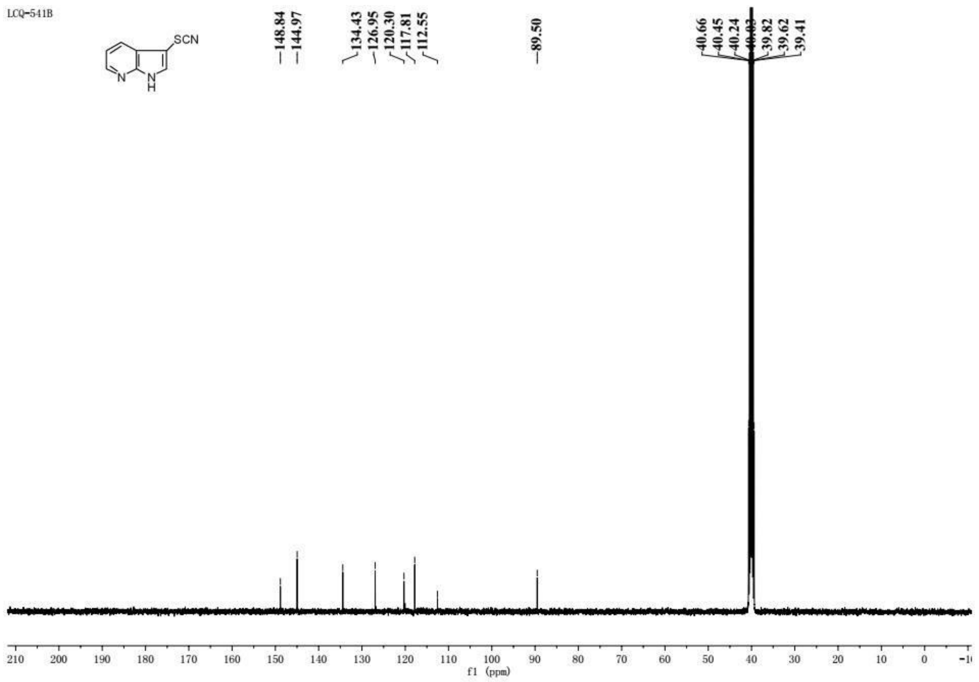

$^{1}$H and $^{13}$C NMR Spectra for **9a**

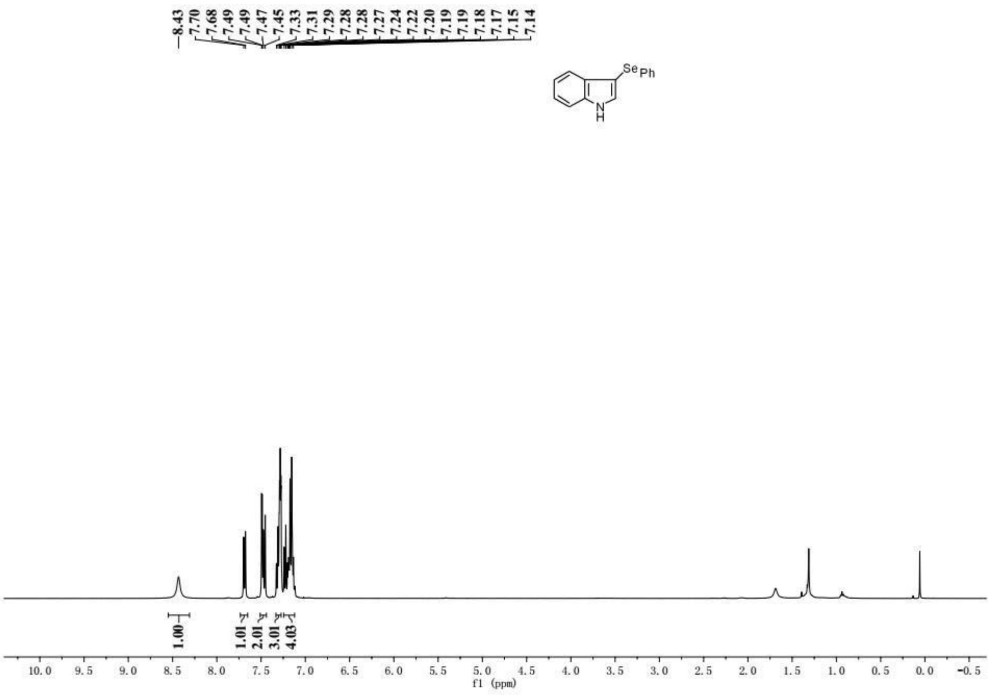


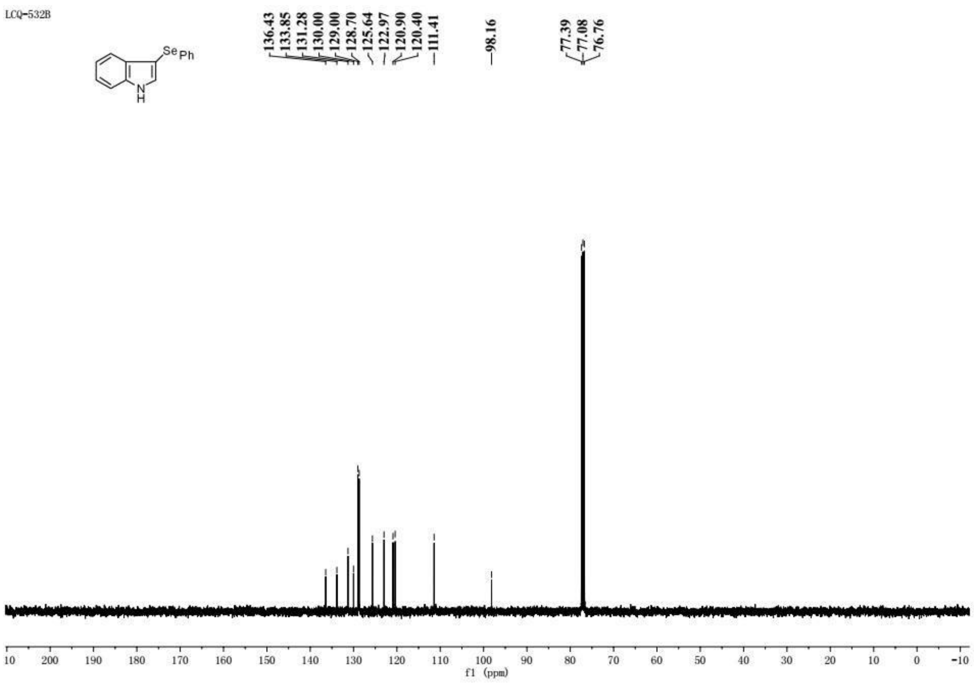

$^{1}H$ and $^{13}C$ NMR Spectra for **9b**

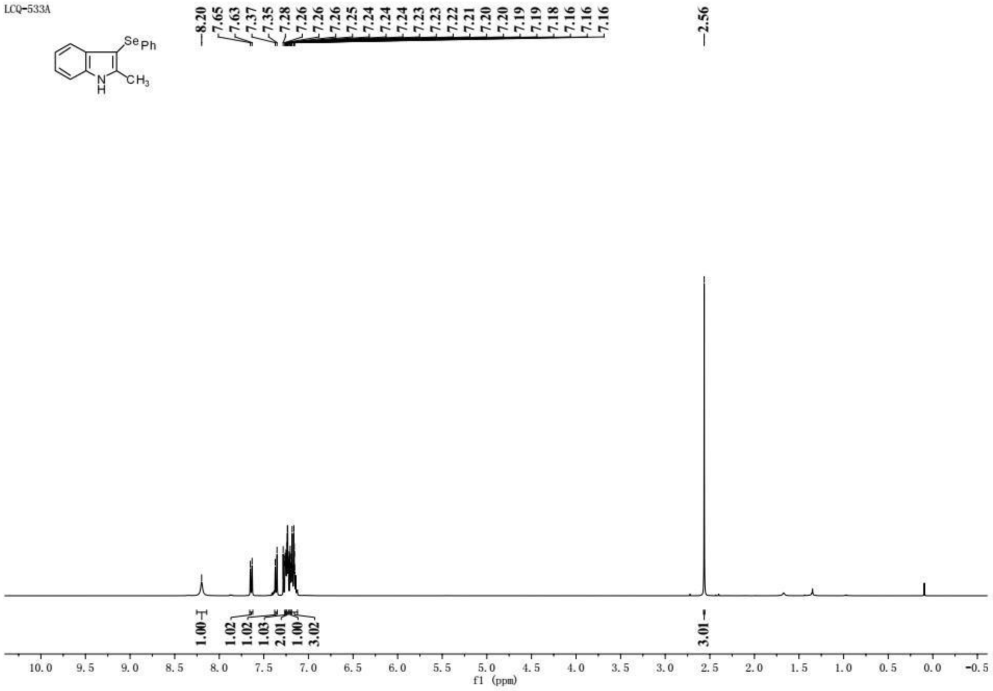


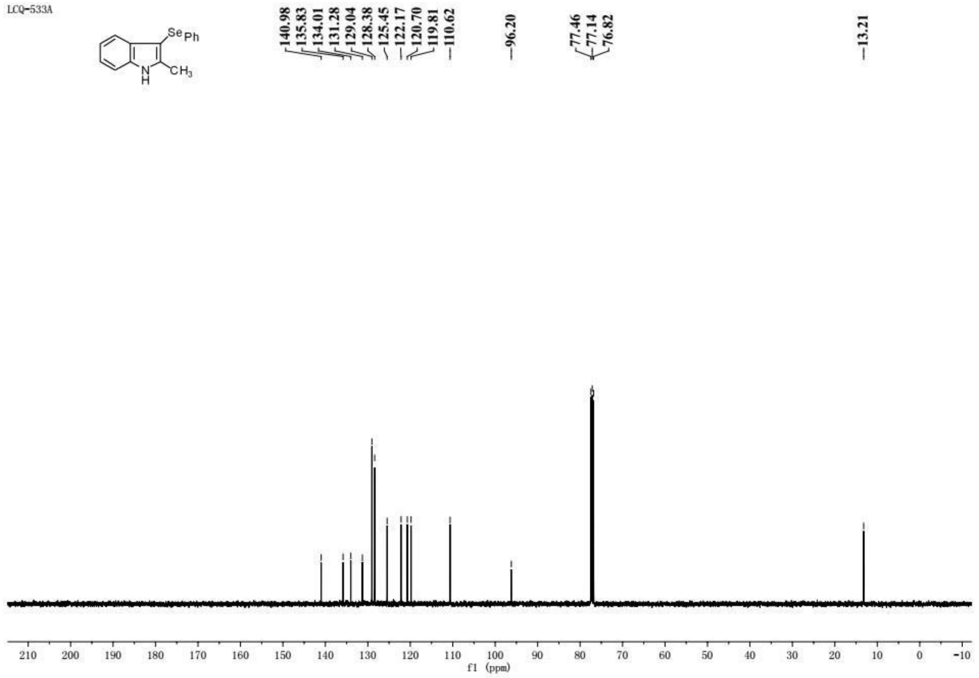

$^1$H and $^{13}$C NMR Spectra for **9c**

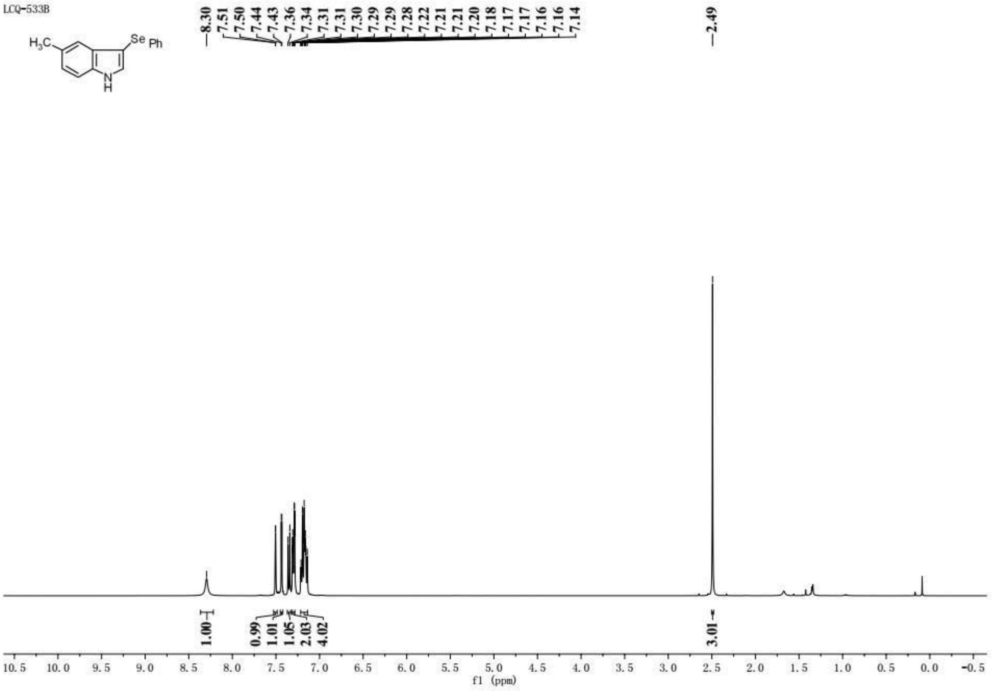


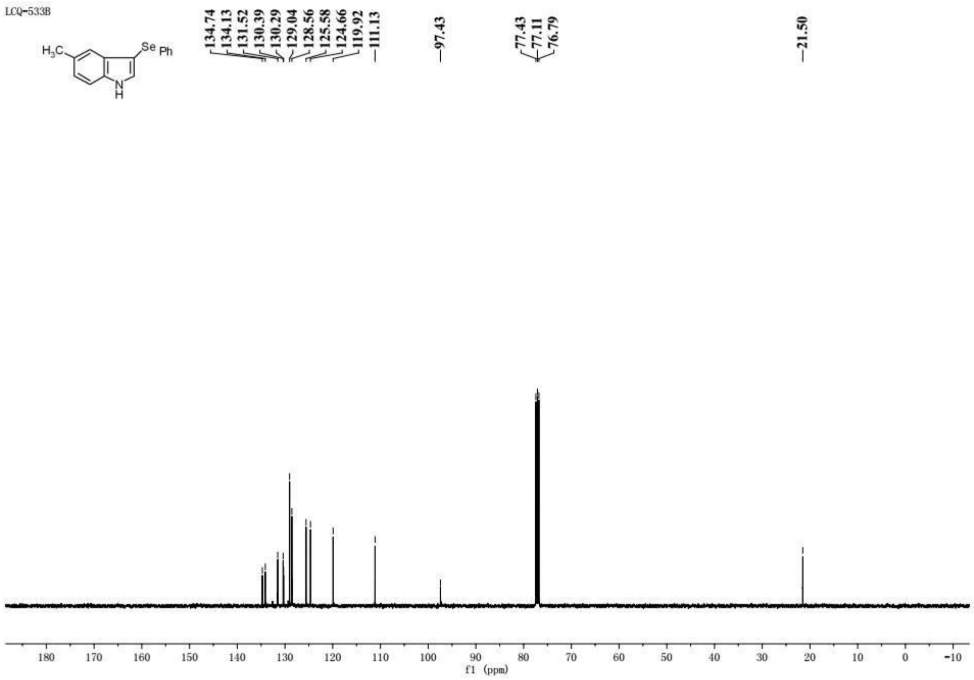

$^{1}H$ and $^{13}C$ NMR Spectra for **9d**

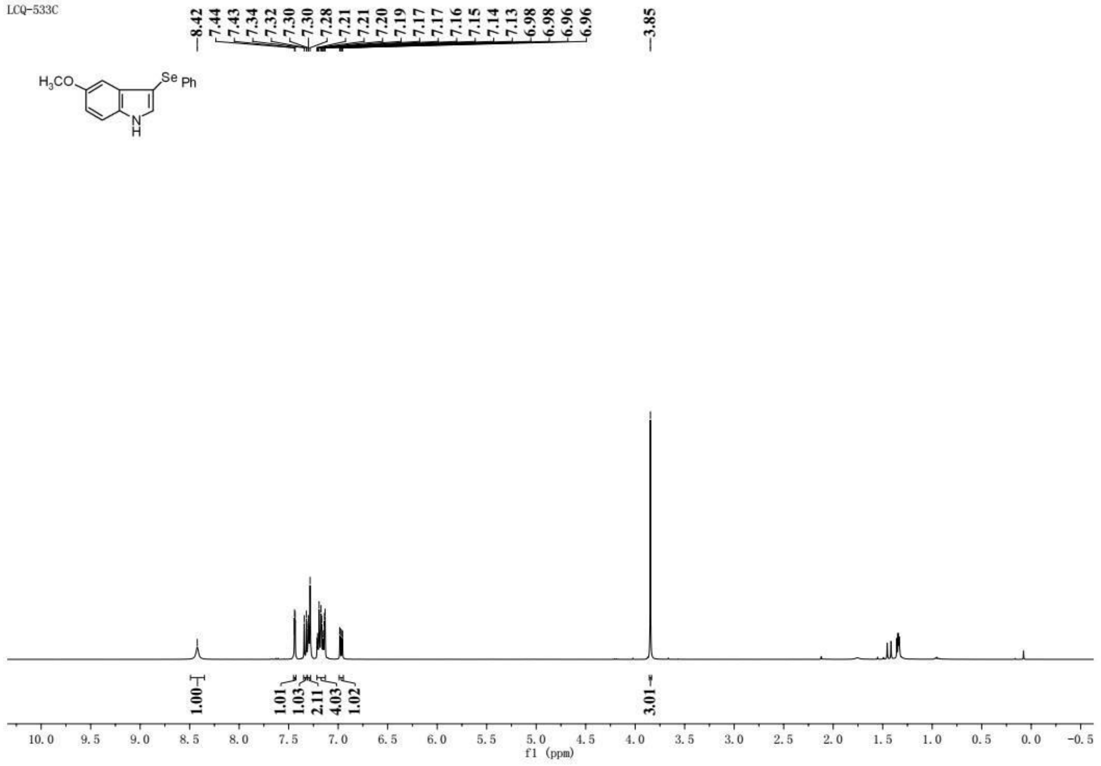


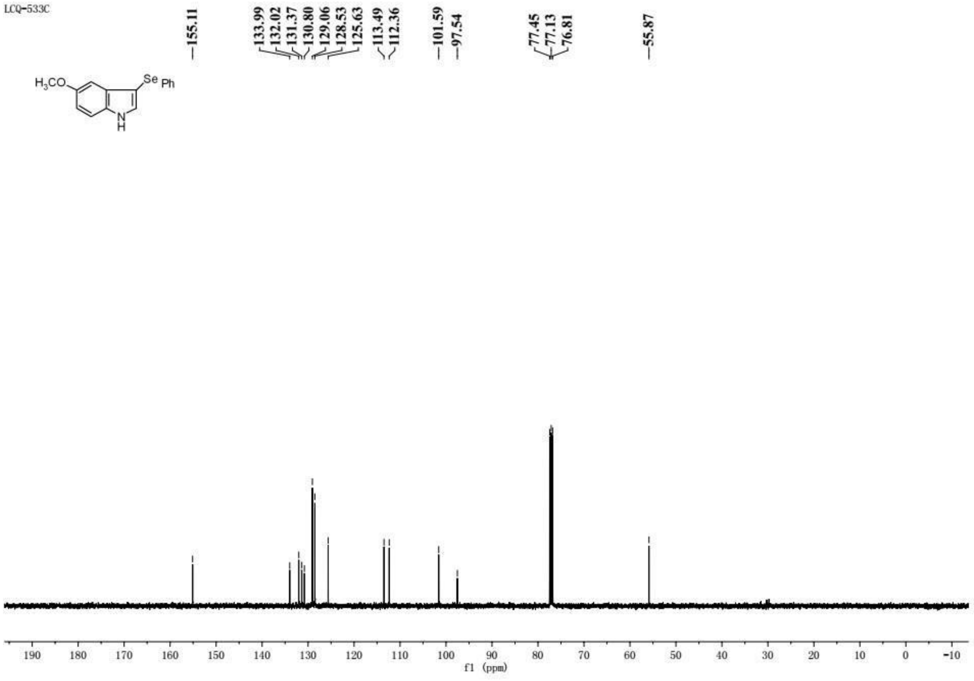

**5. References**


1 C. D. Prasad, S. Kumar, M. Sattar, A. Adhikary and S. Kumar, *Org. Biomol. Chem.*, 2013, **11**, 8036-8040

2 (a) G. Kumaraswamy, R. Rajua and V. Narayanarao, *RSC Adv.*, 2015, **5**, 22718-22723; (b) W. Guo, W. Tan, M. Zhao, K. Tao, L. Y. Zheng, Y. Wu, D. Chen and X. L. Fan, *RSC Adv.*, 2017, **7**, 37739-37742.

3. X. Q. Yang, Y. H. Bao, Z. H. Dai, Q. F. Zhou and F. L. Yang, *Green Chem.*, 2018, **20**, 3727-3731.

4 J. Lin, P. N. Ibrahim, D. R. Artis, C. Zhang, W. R. Wang, S. H. Shi, WO Patent WO2006060535 A2, 2006.

5 X. L. Fang, R. Y. Tang, P. Zhong and J. H. Li, *Synthesis*, 2009, 4183-4189.

6 L. Ackermann, M. Dell'Acqua, S. Fenner, R. Vicente, R. Sandmann. *Org. Lett.*, **2011**, *13*, 2358-2360.

7 Saima, D. Equbal, A. G. Lavekar and A. K. Sinha, *Org. Biomol. Chem.*, 2016, **14**, 6111-6118.

8 X. Zhang, C. G. Wang, H. Jiang and L. H. Sun, *RSC Adv.*, 2018, **8**, 22042-22045.

9 C. Q. Li, P. L. Long, H. P. Wu, H. Q. Yin, F. X. Chen. *Org. Biomol. Chem.*, **2019**, *17*, 7131-7134.

10 H. F. Jiang, W. T. Yu, X. D. Tang, J. X. Li, W. Q. Wu. *J. Org. Chem.*, **2017**, *82*, 9312-9320.

11 D. Wu, J. S. Qiu, P. G. Karmaker, H. Q. Yin and Fu. X. Chen, *J. Org. Chem.*, 2018, **83**, 1576-1583.

12 Y. Cao, J. Liu, F. M. Liu, L. Q. Jiang and W. B. Yi, *Org. Chem. Front.*, 2019, **6**, 825-829.

13 Y. Z. Yu, Y. Zhou, Z. Q. Song and G. Liang, *Org. Biomol. Chem.*, 2018, **16**, 4958-4962.

14 X. Zhang, C. G. Wang, H. Jiang and L. H. Sun, *Chem. Commun.*, 2018, **54**, 8781-8784.
